# Effect of Neutralizing Monoclonal Antibody Treatment on Early Trajectories of Virologic and Immunologic Biomarkers in Patients Hospitalized With COVID-19

**DOI:** 10.1093/infdis/jiad446

**Published:** 2023-11-09

**Authors:** Tomas O Jensen, Greg A Grandits, Mamta K Jain, Thomas A Murray, Birgit Grund, Kathryn Shaw-Saliba, Michael A Matthay, Mahsa Abassi, Magdalena Ardelt, Jason V Baker, Peter Chen, Robin L Dewar, Anna L Goodman, Timothy J Hatlen, Helene C Highbarger, Mark Holodniy, Perrine Lallemand, Sylvain Laverdure, Bradley G Leshnower, David Looney, Charalampos D Moschopoulos, Henry Mugerwa, Daniel D Murray, Eleftherios Mylonakis, Stephanie Nagy-Agren, M Tauseef Rehman, Adam Rupert, Randy A Stevens, Stuart Turville, Amy Weintrob, Katherine Wick, Jens Lundgren, Emily R Ko, for the ACTIV-3/TICO Study Group, David Sahner, John Tierney, Susan E Vogel, Betsey R Herpin, Mary C Smolskis, Laura A McKay, Kelly Cahill, Page Crew, Ratna Sardana, Sharon Segal Raim, Lisa Hensely, Johsua Lorenzo, Rebecca Mock, Judith Zuckerman, Negin Atri, Mark Miller, David Vallee, Lucy Chung, Nayon Kang, Kevin Barrett, Stacey J Adam, Sarah Read, Ruxandra Draghia-Akli, Judy Currier, Eric Hughes, Rachel H Harrigan, Laura Amos, Amy Carlsen, Anita Carter, Gary Collins, Bionca Davis, Eileen Denning, Alain DuChene, Kate Eckroth, Nicole Engen, Alex Frase, Greg Gandits, Birgit Grund, Merrie Harrison, Nancy Hurlbut, Payton Kaiser, Joseph Koopmeiners, Gregg Larson, Sue Meger, Shweta Sharma Mistry, Thomas Murray, Ray Nelson, Kien Quan, Siu Fun Quan, Cavan Reilly, Lianne Siegel, Greg Thompson, David Vock, Jamie Walski, Annetine C Gelijns, Alan J Moskowitz, Emilia Bagiella, Ellen Moquete, Karen O'Sullivan, Mary E Marks, Evan Accardi, Emily Kinzel, Sarah Burris, Gabriela Bedoya, Lola Gupta, Jessica R Overbey, Milerva Santos, Marc A Gillinov, Marissa A Miller, Wendy C Taddei-Peters, Kathleen Fenton, Uriel Sandkovsky, Robert L Gottlieb, Michael Mack, Mezgebe Berhe, Clinton Haley, Emma Dishner, Christopher Bettacchi, Kevin Golden, Erin Duhaime, Madison Ryan, Sarah Burris, Catherine Tallmadge, Lorie Estrada, Felecia Jones, Samatha Villa, Samatha Wang, Raven Robert, Tanquinisha Coleman, Laura Clariday, Rebecca Baker, Mariana Hurutado-Rodriguez, Nazia Iram, Michelle Fresnedo, Allyson Davis, Kiara Leonard, Noelia Ramierez, Jon Thammavong, Krizia Duque, Emma Turner, Tammy Fisher, Dianna Robinson, Desirae Ransom, Nicholas Maldonado, Erica Lusk, Aaron Killian, Adriana Palacious, Edilia Solis, Janet Jerrow, Matthew Watts, Heather Whitacre, Elizabeth Cothran, Peter K Smith, Christina E Barkauskas, Andrew M Vekstein, Emily R Ko, Grace R Dreyer, Neil Stafford, Megan Brooks, Tatyana Der, Marie Witte, Ruwan Gamarallage, John Franzone, Noel Ivey, Rebecca H Lumsden, Nilima Mosaly, Ahmaad Mourad, Thomas L Holland, Mary Motta, Kathleen Lane, Lauren M McGowan, Jennifer Stout, Heather Aloor, Kennesha M Bragg, Barvina Toledo, Beth McLendon-Arvik, Barbara Bussadori, Beth A Hollister, Michelle Griffin, Dana M Giangiacomo, Vicente Rodriguez, Gordon Bokhart, Sharon M Eichman, Patrick E Parrino, Stephen Spindel, Aditya Bansal, Katherine Baumgarten, Johnathan Hand, Derek Vonderhaar, Bobby Nossaman, Sylvia Laudun, DeAnna Ames, Shane Broussard, Nilmo Hernandez, Geralyn Isaac, Huan Dinh, Yiling Zheng, Sonny Tran, Hunter McDaniel, Nicolle Crovetto, Emerson Perin, Briana Costello, Prasad Manian, M Rizwan Sohail, Alexander Postalian, Punit Hinsu, Carolyn Watson, James Chen, Melyssa Fink, Lydia Sturgis, Kim Walker, Kim Mahon, Jennifer Parenti, Casey Kappenman, Aryn Knight, Jeffrey M Sturek, Andrew Barros, Kyle B Enfield, Alexandra Kadl, China J Green, Rachel M Simon, Ashley Fox, Kara Thornton, Amy Adams, Vinay Badhwar, Sunil Sharma, Briana Peppers, Paul McCarthy, Troy Krupica, Arif Sarwari, Rebecca Reece, Lisa Fornaresico, Chad Glaze, Raquel Evans, Fang Di, Shawn Carlson, Tanja Aucremanne, Connie Tennant, Lisa Giblin Sutton, Sabrina Buterbaugh, Roger Williams, Robin Bunner, Jay H Traverse, Frank Rhame, Joshua Huelster, Rajesh Kethireddy, Irena Davies, Julianne Salamanca, Christine Majeski, Paige Skelton, Maria Zarambo, Andrea Sarafolean, Michael E Bowdish, Zea Borok, Noah Wald-Dickler, Douglass Hutcheon, Amytis Towfighi, Mary Lee, Meghan R Lewis, Brad Spellberg, Linda Sher, Aniket Sharma, Anna P Olds, Chris Justino, Edward Loxano, Chris Romero, Janet Leong, Valentina Rodina, Christine Quesada, Luke Hamilton, Jose Escobar, Brad Leshnower, William Bender, Milad Sharifpour, Jeffrey Miller, Woodrow Farrington, Kim T Baio, Mary McBride, Michele Fielding, Sonya Mathewson, Kristina Porte, Missy Maton, Chari Ponder, Elisabeth Haley, Christine Spainhour, Susan Rogers, Derrick Tyler, Ronson J Madathil, Joseph Rabin, Andrea Levine, Kapil Saharia, Ali Tabatabai, Christine Lau, James S Gammie, Maya-Loren Peguero, Kimberly McKernan, Mathew Audette, Emily Fleischmann, Kreshta Akbari, Myounghee Lee, Andrew Chi, Hanna Salehi, Alan Pariser, Phuong Tran Nyguyen, Jessica Moore, Adrienne Gee, Shelika Vincent, Richard A Zuckerman, Alexander Iribarne, Sara Metzler, Samantha Shipman, Haley Johnson, Crystallee Newton, Doug Parr, Leslie Miller, Beth Schelle, Sherry McLean, Howard R Rothbaum, Michael S Alvarez, Shivam P Kalan, Heather H Germann, Jennifer Hendershot, Karen Moroney, Karen Herring, Sharri Cook, Pam Paul, Rebecca Walker-Ignasiak, Crystal North, Cathryn Oldmixon, Nancy Ringwood, Ariela Muzikansky, Richard Morse, Laura Fitzgerald, Haley D Morin, Roy G Brower, Lora A Reineck, Karen Bienstock, Jay H Steingrub, Peter K Hou, Jay S Steingrub, Mark A Tidswell, Lori-Ann Kozikowski, Cynthia Kardos, Leslie DeSouza, Sarah Romain, Sherell Thornton-Thompson, Daniel Talmor, Nathan Shapiro, Konstantinos Andromidas, Valerie Banner-Goodspeed, Michael Bolstad, Katherine L Boyle, Payton Cabrera, Arnaldo deVilla, Joshua C Ellis, Ana Grafals, Sharon Hayes, Conor Higgins, Lisa Kurt, Nicholas Kurtzman, Kimberly Redman, Elinita Rosseto, Douglas Scaffidi, Nathan Shapiro, Michael R Filbin, Kathryn A Hibbert, Blair Parry, Justin Margolin, Brooklynn Hillis, Rhonda Hamer, Kelsey Brait, Caroline Beakes, Brenna McKaig, Eleonore Kugener, Alan E Jones, James Galbraith, Utsav Nandi, Rebekah Peacock, Gregory Hendey, Kirsten Kangelaris, Kimia Ashktorab, Rachel Gropper, Anika Agrawal, Kimberley J Yee, Alejandra E Jauregui, Hanjing Zhuo, Eyad Almasri, Mohamed Fayed, Kinsley A Hubel, Alyssa R Hughes, Rebekah L Garcia, George W Lim, Steven Y Chang, Gregory Hendey, Michael Y Lin, Julia Vargas, Hena Sihota, Rebecca Beutler, Trisha Agarwal, Jennifer G Wilson, Rosemary Vojnik, Cynthia Perez, Jordan H McDowell, Jonasel Roque, Henry Wang, Ryan M Huebinger, Bela Patel, Elizabeth Vidales, Timothy Albertson, Erin Hardy, Richart Harper, Marc A Moss, Amiran Baduashvili, Lakshmi Chauhan, David J Douin, Flora Martinez, Lani L Finck, Jill Bastman, Michelle Howell, Carrie Higgins, Jeffrey McKeehan, Jay Finigan, Peter Stubenrauch, William J Janssen, Christine Griesmer, Olivia VerBurg, Robert C Hyzy, Pauline K Park, Kristine Nelson, Jake I McSparron, Ivan N Co, Bonnie R Wang, Jose Jimenez, Norman Olbrich, Kelli McDonough, Shijing Jia, Sinan Hanna, Michelle N Gong, Lynne D Richardson, Rahul Nair, Brenda Lopez, Omowunmi Amosu, Obiageli Offor, Hiwet Tzehaie, William Nkemdirim, Sabah Boujid, Jarrod M Mosier, Cameron Hypes, Elizabeth Salvagio Campbell, Billie Bixby, Boris Gilson, Anitza Lopez, Christian Bime, Sairam Parthasarathy, Ariana M Cano, R Duncan Hite, Thomas E Terndrup, Herbert P Wiedemann, Kristin Hudock, Hammad Tanzeem, Harshada More, Jamie Martinkovic, Susan Sellers, Judy Houston, Mary Burns, Simra Kiran, Tammy Roads, Sarah Kennedy, Abhijit Duggal, Nirosshan Thiruchelvam, Kiran Ashok, Alexander H King, Omar Mehkri, Siddharth Dugar, Debasis Sahoo, Donald M Yealy, Derek C Angus, Alexandra J Weissman, Tina M Vita, Emily Berryman, Catherine L Hough, Akram Khan, Olivia F Krol, Emmanuel Mills, Mistry Kinjal, Genesis Briceno, Raju Reddy, Kinsley Hubel, Milad K Jouzestani, Madeline McDougal, Rupali Deshmukh, Nicholas J Johnston, Bryce H Robinson, Staphanie J Gundel, Sarah C Katsandres, Peter Chen, Sam S Torbati, Tanyalak Parimon, Antonina Caudill, Brittany Mattison, Susan E Jackman, Po-En Chen, Emad Bayoumi, Cristabelle Ojukwu, Devin Fine, Gwendolyn Weissberg, Katherine Isip, Yunhee Choi-Kuaea, Shaunt Mehdikhani, Tahir B Dar, Nsole Biteghe Fleury Augustin, Dana Tran, Jennifer Emilow Dukov, Yuri Matusov, June Choe, Niree A Hindoyan, Timothy Wynter, Ethan Pascual, Gregg J Clapham, Lisa Herrera, Antonia Caudill, D Shane O'Mahony, Sonam T Nyatsatsang, David M Wilson, Julie A Wallick, Alexandria M Duven, Dakota D Fletcher, Chadwick Miller, D Clark Files, Kevin W Gibbs, Lori S Flores, Mary E LaRose, Leigha D Landreth, D Rafael Palacios, Lisa Parks, Madeline Hicks, Andrew J Goodwin, Edward F Kilb, Caitlan T Lematty, Kerilyn Patti, Abigail Grady, April Rasberry, Peter E Morris, Jamie L Sturgill, Evan P Cassity, Sanjay Dhar, Ashley A Montgomery-Yates, Sarah N Pasha, Kirby P Mayer, Brittany Bissel, Terren Trott, Shahnaz Rehman, Marjolein de Wit, Jessica Mason, Joseph Bledsoe, Kirk U Knowlton, Samuel Brown, Michael Lanspa, Lindsey Leither, Ithan Pelton, Brent P Armbruster, Quinn Montgomery, Naresh Kumar, Melissa Fergus, Karah Imel, Ghazal Palmer, Brandon Webb, Carolyn Klippel, Hannah Jensen, Sarah Duckworth, Andrew Gray, Tyler Burke, Dan Knox, Jenna Lumpkin, Valerie T Aston, Darrin Applegate, Erna Serezlic, Katie Brown, Mardee Merril, Estelle S Harris, Elizabeth A Middleton, Macy A G Barrios, Jorden Greer, Amber D Schmidt, Melissa K Webb, Roert Paine, Sean J Callahan, Lindsey J Waddoups, Misty B Yamane, Wesley H Self, Todd W Rice, Jonathan D Casey, Jakea Johnson, Christopher Gray, Margaret Hays, Megan Roth, Vidya Menon, Moiz Kasubhai, Anjana Pillai, Jean Daniel, Daniel Sittler, Balavenkatesh Kanna, Nargis Jilani, Francisco Amaro, Jessica Santana, Aleksandr Lyakovestsky, Issa Madhoun, Louis Marie Desroches, Nicole Amadon, Alaa Bahr, Imaan Ezzat, Maryanne Guerrero, Joane Padilla, Jessie Fullmer, Inderpreet Singh, Syed Hamad Ali Shah, Rajeev Narang, Polly Mock, Melissa Shadle, Brenda Hernandez, Kevin Welch, Andrea Payne, Gabriela Ertl, Daniel Canario, Isabel Barrientos, Danielle Goss, Mattie DeVries, Ibidolapo Folowosele, Dorothy Garner, Mariana Gomez, Justin Price, Ekta Bansal, Jim Wong, Jason Faulhaber, Tasaduq Fazili, Brian Yeary, Ruth Ndolo, Christina Bryant, Bridgette Smigeil, Philip Robinson, Rana Najjar, Patrice Jones, Julie Nguyen, Christina Chin, Hassan Taha, Salah Najm, Christopher Smith, Jason Moore, Talal Nassar, Nick Gallinger, Amy Christian, D'Amber Mauer, Ashley Phipps, Michael Waters, Karla Zepeda, Jordan Coslet, Rosalynn Landazuri, Jacob Pineda, Nicole Uribe, Jose Ruiz Garcia, Cecilia Barbabosa, Kaitlyn Sandler, J Scott Overcash, Adrienna Marquez, Hanh Chu, Kia Lee, Kimberly Quillin, Andrea Garcia, Pauline Lew, Ralph Rogers, Fadi Shehadeh, Evangelia K Mylona, Matthew Kaczynski, Quynh-Lam Tran, Gregorio Benitez, Biswajit Mishra, Lewis Oscar Felix, Maria Tsikala Vafea, Eleftheria Atalla, Robin Davies, Salma Hedili, Maria Andrea Monkeberg, Sandra Tabler, Britt Harrington, Sreenath Meegada, VenkataSandeep Koripalli, Prithvi Muddana, Lakshay Jain, Chaitanya Undavalli, Parasa Kavya, Mofoluwaso Ibiwoye, Hameed Akilo, Bryce D Lovette, Jamie-Crystal Wylie, Diana M Smith, Kenneth Poon, Paula Eckardt, Rubio-Gomez Heysu, Nithya Sundararaman, Doris Alaby, Candice Sareli, Adriana Sánchez, Laura Popielski, Amy Kambo, Kimberley Viens, Melissa Turner, Michael J Vjecha, Amy Weintrob, Indira Brar, Norman Markowitz, Erika Pastor, Roweena Corpuz, George Alangaden, John McKinnon, Mayur Ramesh, Erica Herc, Nicholas Yared, Odaliz Abreu Lanfranco, Emanuel Rivers, Jennifer Swiderek, Ariella Hodari Gupta, Pardeep Pabla, Sonia Eliya, Jehan Jazrawi, Jeremy Delor, Mona Desai, Aaron Cook, Anja Kathrina Jaehne, Jasreen Kaur Gill, Sheri Renaud, Siva Sarveswaran, Edward Gardner, James Scott, Monica Bianchini, Casey Melvin, Gina Kim, David Wyles, Kevin Kamis, Rachel Miller, Ivor Douglas, Jason Haukoos, Carrie Hicks, Susana Lazarte, Rubria Marines-Price, Alice Osuji, Barbine Tchamba Agbor Agbor, Tianna Petersen, Dena Kamel, Laura Hansen, Angie Garcia, Christine Cha, Azadeh Mozaffari, Rosa Hernandez, James Cutrell, Barbine Tchamba Agbor Agbor, Mina Kim, Natalie DellaValle, Sonia Gonzales, Charurut Somboonwit, Asa Oxner, Lucy Guerra, Michael Hayes, Thi Nguyen, Thanh Tran, Avenette Pinto, Timothy Hatlen, Betty Anderson, Ana Zepeda-Gutierrez, Dannae Martin, Cindi Temblador, Avon Cuenca, Roxanne Tanoviceanu, Martha Prieto, Mario Guerrero, Dannae Martin, Eric Daar, Ramiro Correa, Gabe Hartnell, Glenn Wortmann, Saumil Doshi, Theresa Moriarty, Melissa Gonzales, Kristin Garman, Jason V Baker, Anne Frosch, Rachael Goldsmith, Brian Driver, Christine Frank, Tzivia Leviton, Matthew Prekker, Hodan Jibrell, Melanie Lo, Jonathan Klaphake, Shari Mackedanz, Linh Ngo, Kelly Garcia-Myers, Ken M Kunisaki, Chris Wendt, Anne Melzer, Erin Wetherbee, Dimitri Drekonja, Alexa Pragman, Aimee Hamel, Abbie Thielen, Ken M Kunisaki, Miranda Hassler, Mary Walquist, Michael Augenbraun, Jensen George, Lynette Demeo, Motria Mishko, Lorraine Thomas, Luis Tatem, Jack Dehovitz, Mahsa Abassi, Anne-Marie Leuck, Via Rao, Matthew Pullen, Darlette Luke, Derek LaBar, Theresa Christiansen, Diondra Howard, Kousick Biswas, Cristin Harrington, Amanda Garcia, Tammy Bremer, Tara Burke, Brittany Koker, Anne Davis-Karim, David Pittman, Shikha S Vasudeva, Jaylynn R Johnstone, Kate Agnetti, Ruby Davis, Barbara Trautner, Casey Hines-Munson, John Van, Laura Dillon, Yiqun Wang, Stephanie Nagy-Agren, Shikha Vasudeva, Tracy Ochalek, Erin Caldwell, Edward Humerickhouse, David Boone, William McGraw, David J Looney, Sanjay R Mehta, Scott Thompson Johns, Melissa St John, Jacqueline Raceles, Emily Sear, Stephen Funk, Rosa Cesarini, Michelle Fang, Keith Nicalo, Wonder Drake, Beatrice Jones, Teresa Holtman, Hien H Nguyen, Archana Maniar, Eric A Johnson, Lam Nguyen, Michelle T Tran, Thomas W Barrett, Tera Johnston, John T Huggins, Tatsiana Y Beiko, Heather Y Hughes, William C McManigle, Nichole T Tanner, Ronald G Washburn, Magdalena Ardelt, Patricia A Tuohy, Jennifer L Mixson, Charles G Hinton, Nicola Thornley, Heather Allen, Shannon Elam, Barry Boatman, Brittany J Baber, Rudell Ryant, Brentin Roller, Chinh Nguyen, Amani Morgan Mikail, Marivic Hansen, Paola Lichtenberger, Gio Baracco, Carol Ramos, Lauren Bjork, Melyssa Sueiro, Phyllis Tien, Heather Freasier, Theresa Buck, Hafida Nekach, Mark Holodniy, Aarthi Chary, Kan Lu, Theresa Peters, Jessica Lopez, Susanna Yu Tan, Robert H Lee, Aliya Asghar, Tasadduq Karim Karyn Isip, Katherine Le, Thao Nguyen, Shinn Wong, Dorthe Raben, Daniel D Murray, Tomas O Jensen, Lars Peters, Bitten Aagaard, Charlotte B Nielsen, Katharina Krapp, Bente Rosdahl Nykjær, Christina Olsson, Katja Lisa Kanne, Anne Louise Grevsen, Zillah Maria Joensen, Tina Bruun, Ane Bojesen, Frederik Woldbye, Nick E Normand, Frederik V L Esman, Thomas Benfield, Clara Lundetoft Clausen, Nichlas Hovmand, Simone Bastrup Israelsen, Katrine Iversen, Caecilie Leding, Karen Brorup Pedersen, Louise Thorlacius-Ussing, Michaela Tinggaard, Sandra Tingsgard, Louise Krohn-Dehli, Dorthe Pedersen, Signe Villadsen, Jens-Ulrik Staehr Jensen, Rikke Overgaard, Ema Rastoder, Christian Heerfordt, Caroline Hedsund, Christian Phillip Ronn, Peter Thobias Kamstrup, Dorthe Sandbaek Hogsberg, Christina Bergsoe, Christian Søborg, Nuria M S Hissabu, Bodil C Arp, Lars Ostergaard, Nina Breinholt Staerke, Yordanos Yehdego, Ane Sondergaard, Isik S Johansen, Andreas Arnholdt Pedersen, Fredrikke C Knudtzen, Lykke Larsen, Mathias A Hertz, Thilde Fabricius, Inge K Holden, Susan O Lindvig, Marie Helleberg, Jan Gerstoft, Ole Kirk, Tina Bruun, Tomas Ostergaard Jensen, Birgitte Lindegaard Madsen, Thomas Ingemann Pedersen, Zitta Barrella Harboe, Birgit Thorup Roge, Thomas Michael Hansen, Matilde Kanstrup Glesner, Sandra Valborg Lofberg, Ariella Denize Nielsen, Sebastian Leicht von Huth, Henrik Nielsen, Rikke Krog Thisted, Kristine Toft Petersen, Maria Ruwald Juhl, Daria Podlekareva, Stine Johnsen, Helle Frost Andreassen, Lars Pedersen, Cecilia Ebba Clara Ellinor Lindnér, Lothar Wiese, Lene Surland Knudsen, Nikolaj Julian Skrøder Nytofte, Signe Ravn Havmøller, Maria Expósito, José Badillo, Ana Martínez, Elena Abad, Ana Chamorro, Ariadna Figuerola, Lourdes Mateu, Sergio España, Maria Constanza Lucero, José Ramón Santos, Gemma Lladós, Cristina Lopez, Lydia Carabias, Daniel Molina-Morant, Cora Loste, Carmen Bracke, Adrian Siles, Eduardo Fernández-Cruz, Marisa Di Natale, Sergiu Padure, Jimena Gomez, Cristina Ausin, Eva Cervilla, Héctor Balastegui, Carmen Rodríguez Sainz, Paco Lopez, Javier Carbone, Mariam Escobar, Leire Balerdi, Almudena Legarda, Montserrat Roldan, Laura Letona, José Muñoz, Daniel Camprubí, Jose R Arribas, Rocio Montejano Sánchez, Beatriz Díaz-Pollán, Stefan Mark Stewart, Irene Garcia, Alberto Borobia, Marta Mora-Rillo, Vicente Estrada, Noemi Cabello, M J Nuñez-Orantos, I Sagastagoitia, J R Homen, E Orviz, Adrián Sánchez Montalvá, Juan Espinosa-Pereiro, Pau Bosch-Nicolau, Fernando Salvador, Joaquin Burgos, Jose Luis Morales-Rull, Anna Maria Moreno Pena, Cristina Acosta, Cristina Solé-Felip, Juan P Horcajada, Elena Sendra, Silvia Castañeda, Inmaculada López-Montesinos, Joan Gómez-Junyent, Carlota Gudiol Gonzáles, Guilermo Cuervo, Miquel Pujol, Jordi Carratalà, Sebastià Videla, Huldrych Günthard, Dominique L Braun, Emily West, Khadija M'Rabeth-Bensalah, Mareile L Eichinger, Manuela Grüttner-Durmaz, Christina Grube, Veronika Zink, Josefine Goes, Gerd Fätkenheuer, Jakob J Malin, Tengiz Tsertsvadze, Akaki Abutidze, Nikoloz Chkhartishvili, Revaz Metchurtchlishvili, Marina Endeladze, Marcin Paciorek, Dominik Bursa, Dominika Krogulec, Piotr Pulik, Anna Ignatowska, Andrzej Horban, Elzbieta Bakowska, Justyna Kowaska, Agnieszka Bednarska, Natalia Jurek, Agata Skrzat-Klapaczynska, Carlo Bienkowski, Malgorzata Hackiewicz, Michal Makowiecki, Antoni Platowski, Roman Fishchuk, Olena Kobrynska, Khrystyna Levandovska, Ivanna Kirieieva, Mykhailo Kuziuk, Pontus Naucler, Emma Perlhamre, Lotta Mazouch, Anthony Kelleher, Mark Polizzotto, Catherine Carey, Christina C Chang, Sally Hough, Sophie Virachit, Sarah Davidson, Daniel J Bice, Katherine Ognenovska, Gesalit Cabrera, Ruth Flynn, Barnaby E Young, Po Ying Chia, Tau Hong Lee, Ray J Lin, David C Lye, Sean W X Ong, Ser Hon Puah, Tsin Wen Yeo, Shiau Hui Diong, Juwinda Ongko, He Ping Yeo, Nnakelu Eriobu, Vivian Kwaghe, Habib Zaiyad, Godwin Idoko, Blessing Uche, Poongulali Selvamuthu, Nagalingeswaran Kumarasamy, Faith Ester Beulah, Narayan Govindarajan, Kowsalya Mariyappan, Marcelo H Losso, Cecilia Abela, Renzo Moretto, Carlos G Belloc, Jael Ludueña, Josefina Amar, Marcelo H Losso, Javier Toibaro, Laura Moreno Macias, Lucia Fernandez, Pablo S Frare, Sebastian R Chaio, Valeria Pachioli, Stella M Timpano, Marisa del Lujan Sanchez, Mariana de Paz Sierra, Vanina Stanek, Waldo Belloso, Flavia L Cilenti, Ricardo N Valentini, Martin E Stryjewski, Nicolas Locatelli, Maria C Soler Riera, Clara Salgado, Ines M Baeck, Valentina Di Castelnuovo, Stella M Zarza, Fleur Hudson, Mahesh K B Parmar, Anna L Goodman, Jonathan Badrock, Adam Gregory, Katharine Goodall, Nicola Harris, James Wyncoll, S Bhagani, A Rodger, A Luntiel, C Patterson, J Morales, E Witele, A M Preston, A Nandani, D A Price, Aiden Hanrath, Jeremy Nell, Bijal Patel, Carole Hays, Geraldine Jones, Jade Davidson, Anna L Goodman, T Bawa, M Mathews, A Mazzella, K Bisnauthsing, L Aguilar-Jimenez, F Borchini, S Hammett, Giota Touloumi, Nikos Pantazis, Vicky Gioukari, Tania Souliou, A Antoniadou, K Protopapas, D Kavatha, S Grigoropoulou, R N Tziolos, C Oikonomopoulo, C Moschopoulos, N G Koulouris, K Tzimopoulos, A Koromilias, K Argyraki, P Lourida, P Bakakos, I Kalomenidis, V Vlachakos, Z Barmparessou, E Balis, S Zakynthinos, I Sigala, N Gianniou, E Dima, S Magkouta, E Synolaki, S Konstanta, M Vlachou, P Stathopoulou, P Panagopoulos, V Petrakis, D Papazoglou, E Tompaidou, E Isaakidou, G Poulakou, V Rapti, K Leontis, T Nitsotolis, K Athanasiou, K Syrigos, K Argyraki, M D Myrodia, K Kyriakoulis, I Trontzas, M Arfara-Melanini, V Kolonis, Cissy Kityo, Henry Mugerwa, Francis Kiweewa, Ivan Kimuli, Joseph Lukaakome, Christoher Nsereko, Gloria Lubega, Moses Kibirige, William Nakahima, Deus Wangi, Evelyne Aguti, Lilian Generous, Rosemary Massa, Margaret Nalaki, Felix Magala, Phiona Kaweesi Nabaggala, Robert Kidega, Cissy Kityo, Henry Mugerwa, Oryem Daizy Faith, Apio Florence, Ocung Emmanuel, Mugoonyi Paul Beacham, Amone Geoffrey, Dridah Nakiboneka, Paska Apiyo, Francis Kiweewa, Bruce Kirenga, Ivan Kimuli, Angella Atukunda, Winters Muttamba, Kyeyume Remmy, Ivan Segawa, Nsubuga Pheona, David Kigere, Queen Lailah Mbabazi, Ledra Boersalino, Grace Nyakoolo, Francis Kiweewa, Aniongo Fred, Alice Alupo, Doryn Ebong, Edson Monday, Ritah Norah Nalubwama, Milton Kainja, Munu Ambrose, Vanon Kwehayo, Mary Grace Nalubega, Augustine Ongoli, Stephen Obbo, Nicholus Sebudde, Jeniffer Alaba, Geoffrey Magombe, Harriet Tino, Emmanuel Obonya EE, Joseph Lutaakome, Jonathan Kitonsa, Martin Onyango, Tukamwesiga Naboth, Hadijah Naluyinda, Regina Nanyunja, Muttiibwa Irene, Biira Jane, Kyobejja Wimfred, Ssemazzi Leonard, Tkiinomuhisha Deus, Namasaba Babra, Paul Taire, Joseph Lutaakome, Evelyn Nabankema, Joseph Ogavu, Oscar Mugerwa, Ivan Okoth, Raymond Mwebaze, Timothy Mugabi, Anthony Makhoba, Phiona Arikiriza, Nabuuma Theresa, Hope Nakayima, Kisuule Frank, Patrícia Ramgi, Kássia Pereira, Anu Osinusi, Huyen Cao, Paul Klekotka, Karen Price, Ajay Nirula, Suzette Osei, Craig Tipple, Angela Wills, Amanda Peppercorn, Helen Watson, Rajesh Gupta, Elizabeth Alexander, Erik Mogalian, Leo Lin, Xiao Ding, David Margolis, Li Yan, Jean-Luc Girardet, Ji Ma, Zhi Hong, Quing Zhu, Seth Seegobin, Michael Gibbs, Mickel Latchman, Katarzyna Hasior, Jerome Bouquet, Jianxin Wei, Katie Streicher, Albert Schmelzer, Dennis Brooks, Jonny Butcher, Dimitar Tonev, Douglas Arbetter, Philippe Damstetter, Philippe Legenne, Michael Stumpp, Susana Goncalves, Krishnan Ramanathan, Richa Chandra, Beth Baseler, Marc Teitelbaum, Adam Schechner, H Preston Holley, Shirley Jankelevich, Amy Adams, Nancy Becker, Suzanne Dolney, Debbie Hissey, Shelly Simpson, Mi Ha Kim, Joy Beeler, Liam Harmon, Mabel Asomah, Yvonne Jato, April Stottlemyer, Olivia Tang, Sharon Vanderpuye, Lindsey Yeon, Molly Buehn, Vanessa Eccard-Koons, Sadie Frary, Leah MacDonald, Jennifer Cash, Lisa Hoopengardner, Jessica Linton, Marylu Schaffhauser, Michaela Nelson, Mary Spinelli-Nadzam, Calvin Proffitt, Christopher Lee, Theresa Engel, Laura Fontaine, C K Osborne, Matt Hohn, Michael Galcik, DeeDee Thompson, Stacey Kopka, Denise M Shelley, Gregg Mendez, Shawn Brown, Sara Albert, Abby Balde, Michelle Baracz, Mona Bielica, Shere Billouin-Frazier, Jay Choudary, Mary Dixon, Carolyn Eyler, Leanne Frye, Jensen Gertz, Lisa Giebeig, Neelam Gulati, Liz Hankinson, Debi Hogarty, Lynda Huber, Gary Krauss, Eileen Lake, Meryan Manandhar, Erin Rudzinski, Jen Sandrus, Connie Suders, Ven Natarajan, Adam W Rupert, Michael Baseler, Danielle Lynam, Tom Imamichi, Sylvain Laverdure, Ashley McCormack, Sharada Paudel, Kyndal Cook, Kendra Haupt, Ayub Khan, Allison Hazen, Yunden Badralmaa, Kenneth Smith, Bhakti Patel, Amanda Kubernac, Robert Kubernac, Marie L Hoover, Courtney Solomon, Marium Rashid, Joseph Murphy, Craig Brown, Nadine DuChateau, Sadie Ellis, Adam Flosi, Lisa Fox, Les Johnson, Rich Nelson, Jelena Stojanovic, Amy Treagus, Christine Wenner, Richard Williams

**Affiliations:** Centre of Excellence for Health, Immunity, and Infections, Rigshospitalet, University of Copenhagen, Copenhagen, Denmark; Division of Biostatistics, University of Minnesota, Minneapolis, Minnesota, USA; Southwestern Medical Center, University of Texas, Dallas, Texas USA; Division of Biostatistics, University of Minnesota, Minneapolis, Minnesota, USA; School of Statistics, University of Minnesota, Minneapolis, Minnesota, USA; National Institute of Allergy and Infectious Diseases, National Institutes of Health, Bethesda, Maryland, USA; Division of Pulmonary and Critical Care Medicine, University of California SanFrancisco, San Francisco, California, USA; Division of Infectious Diseases and International Medicine, University of Minnesota, Minneapolis, Minnesota, USA; Division of Pulmonary and Critical Care Medicine, University of California SanFrancisco, San Francisco, California, USA; Division of Infectious Diseases and International Medicine, University of Minnesota, Minneapolis, Minnesota, USA; Division of Infectious Diseases, Hennepin Healthcare, Minneapolis, Minnesota, USA; Women's Guild Lung Institute, Department of medicine and Biomedical Sciences, Cedars-Sinai Medical Center, Los Angeles, California, USA; Frederick National Laboratory for Cancer Research, Frederick, Maryland, USA; Medical Research Council Clinical Trials Unit, University College London, London, United Kingdom; Department of Infectious Diseases, Guy's and St Thomas’ National Health Service Foundation Trust, London, United Kingdom; Lundquist Institute, Harbor-University of California Los Angeles Medical Center, Torrance, California, USA; Frederick National Laboratory for Cancer Research, Frederick, Maryland, USA; Department of Medicine, Infectious Diseases, Stanford University, Stanford, California, USA; Veterans Affairs Palo Alto Health Care System, Palo Alto, California, USA; Frederick National Laboratory for Cancer Research, Frederick, Maryland, USA; Laboratory of Human Retrovirology and Immunoinformatics, Frederick National Laboratory for Cancer Research, Frederick, Maryland, USA; Division of Cardiothoracic Surgery, Emory School of Medicine, Atlanta, Georgia, USA; Division of Infectious Diseases and Global Public Health, University of California San Diego, San Diego, California, USA; School of Medicine, National and Kapodistrian University of Athens, Athens, Greece; Joint Clinical Research Centre, Kampala, Uganda; Centre of Excellence for Health, Immunity, and Infections, Rigshospitalet, University of Copenhagen, Copenhagen, Denmark; Department of Medicine, Houston Methodist Hospital, Houston, Texas, USA; Salem Veterans Affairs Medical Center, Virginia, USA; Virginia Tech Carilion School of Medicine, Virginia, USA; Frederick National Laboratory for Cancer Research, Frederick, Maryland, USA; Frederick National Laboratory for Cancer Research, Frederick, Maryland, USA; Frederick National Laboratory for Cancer Research, Frederick, Maryland, USA; The Kirby Institute, University of New South Wales, Sydney, Australia; Infectious Diseases Section, Washington DC Veterans Affairs Medical Center, Washington, District of Columbia, USA; Department of Internal Medicine, University of California Davis, Davis, California, USA; Centre of Excellence for Health, Immunity, and Infections, Rigshospitalet, University of Copenhagen, Copenhagen, Denmark; Division of General Internal Medicine, Department of Medicine, Duke University Health System, Durham, North Carolina, USA

**Keywords:** COVID-19, neutralizing monoclonal antibody, plasma nucleocapsid antigen, anti-nucleocapsid antibody, inflammatory biomarkers

## Abstract

**Background:**

Neutralizing monoclonal antibodies (nmAbs) failed to show clear benefit for hospitalized patients with coronavirus disease 2019 (COVID-19). Dynamics of virologic and immunologic biomarkers remain poorly understood.

**Methods:**

Participants enrolled in the Therapeutics for Inpatients with COVID-19 trials were randomized to nmAb versus placebo. Longitudinal differences between treatment and placebo groups in levels of plasma nucleocapsid antigen (N-Ag), anti-nucleocapsid antibody, C-reactive protein, interleukin-6, and D-dimer at enrollment, day 1, 3, and 5 were estimated using linear mixed models. A 7-point pulmonary ordinal scale assessed at day 5 was compared using proportional odds models.

**Results:**

Analysis included 2149 participants enrolled between August 2020 and September 2021. Treatment resulted in 20% lower levels of plasma N-Ag compared with placebo (95% confidence interval, 12%–27%; *P* < .001), and a steeper rate of decline through the first 5 days (*P* < .001). The treatment difference did not vary between subgroups, and no difference was observed in trajectories of other biomarkers or the day 5 pulmonary ordinal scale.

**Conclusions:**

Our study suggests that nmAb has an antiviral effect assessed by plasma N-Ag among hospitalized patients with COVID-19, with no blunting of the endogenous anti-nucleocapsid antibody response. No effect on systemic inflammation or day 5 clinical status was observed.

**Clinical Trials Registration:**

NCT04501978.

## Teaser

Treatment with neutralizing monoclonal antibody lowered the trajectory of plasma nucleocapsid antigen over 5 days in patients hospitalized with COVID-19. No effect was seen on anti-nucleocapsid antibody, C-reactive protein, interleukin-6, D-dimer, or early clinical outcome by day 5.

Coronavirus disease 2019 (COVID-19) led to tremendous morbidity and mortality as well as remarkable scientific gains, providing critical antiviral and immunomodulatory treatments [1–11]. While neutralizing monoclonal antibody (nmAb) treatments benefitted outpatients with mild, early COVID-19, their impact in hospitalized patients have not shown consistently significant advantages over standard of care including remdesivir [1, 8, 12, 13].

Clinical data have suggested a correlation between ongoing viral replication, inflammation, and disease severity in hospitalized patients with COVID-19 [14–17], and several randomized controlled trials have indicated that there are subgroups of hospitalized patients who may benefit from treatment with nmAb. This includes patients with low baseline titer of anti-spike antibodies (anti-S Ab) [7, 8], patients with high baseline concentration of plasma SARS-CoV-2 nucleocapsid antigen (plasma N-Ag) [7], and patients who require a high level of respiratory support (high-flow nasal oxygen [HFNO] or noninvasive ventilation [NIV]) [1]. The dynamics of virological and immunological biomarkers over time have not been described and may further the understanding of trial results. Ultimately, this information could inform treatment strategies for managing COVID-19 at the point of hospital admission, aid in the design of future antiviral treatment and algorithms, and allow prognostic enrichment strategies in future clinical trials.

The Therapeutics for Inpatients with COVID-19 (TICO) trial platform, sponsored by the US National Institutes of Health within the Accelerating COVID-19 Therapeutic Interventions and Vaccines (ACTIV) program, conducted 4 international, blinded, randomized, placebo-controlled trials of nmAbs in hospitalized patients with COVID-19 receiving standard of care [1, 12, 13, 18]. This study reports measurement of plasma N-Ag at baseline and on days 1, 3, and 5 of follow-up, together with anti-nucleocapsid antibodies (anti-N Ab), anti-S Ab neutralizing activity, C-reactive protein (CRP), interleukin 6 (IL-6), and D-dimer. We describe the impact of nmAb on early trajectories of these measurements compared with placebo.

## METHODS

### Study Population

Between 5 August 2020 and 30 September 2021, TICO/ACTIV-3 trials enrolled 2254 hospitalized adult patients with laboratory-confirmed severe acute respiratory syndrome coronavirus 2 (SARS-CoV-2) infection, symptoms for ≤12 days, and no organ failure or major extrapulmonary manifestations of COVID-19. These trials evaluated the nmAbs bamlanivimab (Eli Lilly and Company) between August and October 2020 [13], sotrovimab (Vir Biotechnology and GlaxoSmithKline) between December 2020 and March 2021 [12], amubarvimab-romlusevimab (Brii Biosciences) between December 2020 and March 2021 [12], and tixagevimab-cilgavimab (AstraZeneca) between February and September 2021 [1]. Only the tixagevimab-cilgavimab trial passed the early futility assessment, and a higher number of patients were therefore enrolled in this trial. Participants were randomized to the specific nmAb or placebo, and remdesivir was provided as part of standard of care to all participants unless contraindicated. In some cases, a placebo participant was used as a control for multiple trials. Participants were enrolled at 108 sites in Denmark, Greece, Poland, Uganda, Singapore, Spain, Switzerland, the United Kingdom, and the United States. The trials are registered with ClinicalTrials.gov, NCT04501978.

Patients not requiring oxygen or receiving oxygen supplementation via conventional nasal cannula were eligible for enrollment in all trials, and the bamlanivimab and tixagevimab-cilgavimab trials also enrolled patients receiving HFNO or NIV. Patients requiring invasive mechanical ventilation were excluded in all trials. For our study, we included all participants who had a baseline sample (day 0) taken at the time of enrollment and at least 1 follow-up sample from days 1, 3, or 5 analyzed with the laboratory measurement of interest. Written informed consent for trial participation was obtained from each enrolled patient or a legally authorized representative, as applicable.

### Laboratory Measurements

Samples were stored at −70°C at a central repository, Advanced BioMedical Laboratories (Cinnaminson, NJ). Levels of plasma N-Ag, anti-N Ab, anti-S Ab neutralizing activity, CRP, IL-6, and D-dimer were determined centrally by the Frederick National Laboratory (Frederick, MD), blinded to treatment group.

The concentration of plasma N-Ag was determined using the Quanterix SARS-CoV-2 N Protein Antigen assay (Quanterix); the lower level of detection was 3 ng/L.

The level of anti-N Ab was measured using the BioRad Platelia SARS-CoV-2 Total Ab assay (BioRad). Results of the assay were reported as signal to cutoff ratio (S/C ratio) defined as the specimen optical density divided by that of the control. An S/C ratio above 1 was considered positive.

The level of anti-S Ab neutralizing activity was evaluated using the GenScript SARS-CoV-2 cPass Surrogate Virus Neutralization assay (GenScript). Levels were expressed as percent binding inhibition, and a positive result was defined as 30% binding inhibition or more [19].

Serum levels of CRP and plasma levels of IL-6 were measured using electrochemiluminescence (Meso Scale Discovery). Plasma D-dimer was measured by an enzyme-linked fluorescent assay on a VIDAS instrument (BioMerieux). Upper limits of normal for CRP, IL-6, and D-dimer were 10 mg/L, 2 ng/L, and 0.5 mg/L, respectively.

For Delta variant analysis, SARS-CoV-2 viral RNA was extracted from a midturbinate nasal swab collected at baseline. All participants enrolled after 1 May 2021 were tested for the presence of the Delta variant using a reverse transcription polymerase chain reaction (RT-PCR) assay specifically designed to detect the N-terminal domain region of the spike gene with the N gene serving as a positive control, as described in the original trial publication [1]. Participants enrolled prior to this date were considered infected with a non-Delta SARS-CoV-2 variant. Confirmation of the RT-PCR was done using whole genome sequencing as described previously [7]. Concordance was 99.9% (n = 811).

### Statistical Analysis

In the bamlanivimab trial, participants were randomized 1:1 to bamlanivimab versus placebo, and enrollment completed before the next nmAb trial started. For the majority of the second trial, participants were randomized 1:1:1 to sotrovimab, amubarvimab-romlusevimab, or placebo; the last month of enrolment also included tixagevimab-cilgavimab as a third active arm. Almost all participants in the tixagevimab-cilgavimab trial were randomized 1:1 to nmAb versus placebo. Because some placebo participants were used for multiple trials, more participants overall were randomized to nmAb than to placebo. To avoid bias in the treatment comparisons due the changes in randomization proportions over time, we defined 5 strata, 1 for each combination of nmAbs that were available to participants at the time of randomization. Each participant was assigned to 1 of the 5 strata. All comparisons of pooled nmAb versus placebo were stratified by this variable. For the comparisons of each individual nmAb versus placebo, we included all participants into the placebo group who were randomized contemporaneously to placebo, resulting in approximately equal numbers of participants in the active and matched placebo groups.

Levels of plasma N-Ag, CRP, IL-6, and D-dimer were log-transformed for analyses, and results were back-transformed to the original scale; thus, these biomarker levels were summarized by geometric means, and treatment differences were presented as geometric mean ratios. Anti-N and anti-S Ab neutralizing activity were analyzed on the original scale, and summarized as means and differences of means.

For each laboratory measurement, longitudinal plots of means with 95% confidence intervals (CIs) at days 0, 1, 3, and 5 were presented by nmAb versus placebo groups. Longitudinal differences between groups were estimated using linear mixed models for each laboratory measurement, modeling the biomarker levels on days 1, 3, and 5 as repeated measures over the 3 visits, with fixed effects for treatment group, visit (categorical variable), baseline oxygen requirement (pulmonary ordinal scale), baseline value of the laboratory measurement being analyzed, and random intercepts by subject. Comparisons of the pooled nmAb group versus placebo also included the study stratum as covariate. The longitudinal treatment effect was estimated with 95% CIs as the coefficient for treatment group indicator, which reflects an average treatment effect across days 1, 3, and 5. A treatment by day (categorical variable) interaction term was added to the above models to test whether the treatment effect varied across the 3 follow-up days.

Similar longitudinal models were used to compare biomarker trajectories between the nmAb and placebo groups within subgroups defined by baseline factors: age, sex, comorbidities (Supplementary Table 1), viral variant, symptom duration, pulmonary ordinal scale, plasma N-Ag concentration, anti-N Ab serostatus, anti-S Ab serostatus, and COVID-19 vaccination status. The treatment effect with 95% CI was estimated for each subgroup, and an interaction term (treatment group by subgroup indicator) was added to test whether the nmAb treatment effect differed across subgroups.

Because the biomarker changes we examined occurred over the first 5 days, we chose an outcome reflective of changes over 5 days. Thus, association between nmAb treatment and the day 5 pulmonary ordinal scale, collected as a secondary early outcome in TICO/ACTIV-3 (Supplementary Table 2), was used to correlate the effect of nmAb on biomarkers with clinical outcome. This association was estimated as the common odds ratio, using proportional odds models, reflecting the odds of being in a better category for participants in the nmAb group compared with placebo.

Nominal *P* values ≤.05 were considered significant, and the cutoff was lowered to ≤.01 in the subgroup analysis due to the high number of comparisons. Statistical analyses were conducted using SAS (version 9.4) and R (version 4.1).

## RESULTS

### Baseline Clinical Characteristics

In total, 2149 participants had laboratory measurements at baseline and 1 or more follow-up time points (days 1, 3, or 5). Baseline characteristics are summarized in Table 1, overall, and by nmAb trial. Overall, the median age was 57 years (interquartile range [IQR], 46–68; total range, 19–100), 58% were male, 83.4% had a history of at least 1 chronic illness, and only 8.8% were fully vaccinated.

**Table 1.** Baseline Characteristics in Total Cohort and Individual Trials

		Individual Trials of nmAb vs Placebo			
Characteristic	Total	Bamlanivimab	Sotrovimab	Amubarvimab/Romlusevimab	Tixagevimab/Cilgavimab
Participants, total (treatment)	2149 (1178)	306 (159)	254 (172)	250 (167)	1339 (680)
Age, y, median (IQR)	57 (46–68)	61 (49–71)	60 (50–72)	60 (49–71)	54 (44–66)
Female sex, No. (%)	903 (42.0)	132 (43.1)	104 (40.9)	107 (42.8)	560 (41.8)
Geographical region, No. (%)					
Africa	86 (4.0)	0 (0.0)	0 (0.0)	0 (0.0)	86 (6.4)
Asia	24 (1.1)	1 (0.3)	0 (0.0)	0 (0.0)	23 (1.7)
Europe	325 (15.1)	37 (12.1)	16 (6.3)	12 (4.8)	260 (19.4)
North America	1714 (79.8)	268 (87.6)	238 (93.7)	238 (95.2)	970 (72.4)
Comorbidities, No. (%)					
Cardiovascular disease	1033 (48.1)	165 (53.9)	147 (57.9)	148 (59.2)	573 (42.8)
Chronic kidney disease	211 (9.8)	32 (10.5)	37 (14.6)	19 (7.6)	123 (9.2)
Chronic lung disease	326 (15.2)	44 (14.4)	40 (15.7)	44 (17.6)	198 (14.8)
Diabetes	618 (28.8)	89 (29.1)	98 (38.6)	87 (34.8)	344 (25.7)
Hepatic impairment	36 (1.7)	1 (0.3)	5 (2.0)	6 (2.4)	24 (1.8)
HIV	36 (1.7)	2 (0.7)	5 (2.0)	2 (0.8)	27 (2.0)
Immunocompromised	328 (15.3)	29 (9.5)	33 (130)	36 (14.4)	230 (17.2)
Obesity	1151 (53.7)	161 (52.8)	141 (55.5)	129 (51.6)	720 (54.0)
Any of the above	1792 (83.4)	256 (83.7)	228 (89.8)	223 (89.2)	1085 (81.0)
COVID-19 treatments, No. (%)					
Corticosteroids^a^	1465 (68.2)	155 (50.7)	165 (65.0)	156 (62.4)	989 (73.9)
Heparin, therapeutic dose	85 (4.0)	6 (2.0)	6 (2.4)	4 (1.6)	69 (5.2)
Remdesivir	1995 (92.6)	294 (96.1)	231 (90.9)	224 (89.2)	1246 (92.7)
COVID-19 vaccination status, No. (%)					
Fully vaccinated^b^	190 (8.8)	0 (0.0)	1 (0.4)	0 (0.0)	189 (14.1)
Partially vaccinated	195 (9.1)	0 (0.0)	17 (6.7)	15 (6.0)	163 (12.2)
Not vaccinated	1764 (82.1)	306 (100)	236 (92.9)	235 (94.0)	987 (73.7)
Symptom duration, d, median (IQR)	8 (6–10)	7 (5–9)	8 (5–9)	8 (5–9)	8 (6–10)
Pulmonary ordinal scale, No. (%)					
No supplementary oxygen	553 (25.7)	87 (28.4)	84 (33.1)	80 (32.0)	302 (22.6)
< 4 L oxygen per min	816 (38.0)	111 (36.3)	114 (44.9)	102 (40.8)	489 (36.5)
≥ 4 L oxygen per min	582 (27.1)	62 (20.3)	56 (22.0)	68 (27.2)	396 (29.6)
HFNO or NIV	198 (9.2)	46 (15.0)	0 (0.0)	0 (0.0)	152 (11.4)
Viral variant, No. (%)					
Delta	658 (30.9)	0 (0.0)	0 (0.0)	0 (0.0)	658 (49.8)
Not Delta	1474 (69.1)	306 (100)	254 (100)	250 (100)	664 (50.2)
Nucleocapsid antigen result, No. (%)					
Positive	2033 (94.6)	291 (95.1)	241 (94.9)	239 (95.6)	1262 (94.3)
Positive, result ≥1000 ng/L	899 (41.9)	154 (50.3)	103 (40.6)	108 (43.2)	534 (39.9)
Antibody status, No. (%)					
Anti-nucleocapsid positive	1330 (61.9)	181 (59.2)	148 (58.3)	158 (63.2)	843 (63.0)
Anti-spike positive	1065 (49.6)	153 (50.0)	101 (39.8)	106 (42.4)	705 (52.7)
Biomarker results, median (IQR)					
C-reactive protein, mg/L	31 (14–56)	32 (15–59)	26 (13–49)	32 (14–54)	31 (14–56)
Interleukin-6, ng/L	6 (2–14)	7 (2–12)	6 (2–12)	6 (3–13)	6 (2–15)
D-dimer, mg/L	0.9 (0.6–1.4)	0.9 (0.6–1.4)	0.9 (0.6–1.4)	0.9 (0.7–1.4)	0.9 (0.6–1.5)

Abbreviations: COVID-19, coronavirus disease 2019; HFNO, high-flow nasal oxygen; HIV, human immunodeficiency virus; IQR, interquartile range; NIV, noninvasive ventilation; nmAb, neutralizing monoclonal antibody.

^a^Treatment was 10 mg or more of prednisolone or equivalent.

^b^Full primary vaccination course completed; symptoms started at least 14 days after the last dose.

The median duration from symptom onset to enrollment was 8 days (IQR, 6–10), and 74.3% of participants required oxygen supplementation at the time of enrollment with only a small proportion (9.2%) needing HFNO or NIV. Use of systemic corticosteroids was common (68.2%), and a small proportion received therapeutic heparin dosing (4.0%). Most participants (60.3%) were treated with remdesivir prior to enrollment, and because the trial provided remdesivir, post-randomization use increased to 92.6%. Of the participants, 30.9% were infected with the Delta variant.

At enrollment, plasma N-Ag was detected in almost all participants (94.6%), while 61.9% had a positive baseline test for anti-N Ab (median S/C ratio 2.4), and 49.6% had a positive test for anti-S Ab (mean 38.1% binding inhibition). The baseline median values of CRP, IL-6, and D-dimer were 31 mg/L, 6 ng/L, and 0.9 mg/L, respectively.

### Effect of nmAbs on Biomarker Trajectories Compared With Placebo

**Figure 1.**
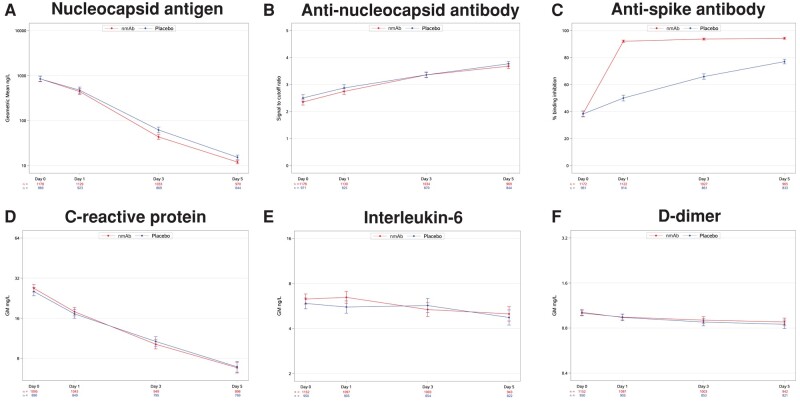
Line plots of mean biomarker levels (with 95% confidence intervals) over time by neutralizing monoclonal antibody treatment and placebo groups. *A*, *D*, *E*, and *F,* Levels as geometric means; these biomarkers were analyzed on the log scale and back transformed. *B* and *C*, Levels as means analyzed on the original scale.

In the combined cohort of all 4 nmAb trials, plasma N-Ag levels decreased steadily from baseline through day 5 in both nmAb and placebo groups with the nmAb group having a small but significantly greater decline by day 3 (Figure 1*A* and Table 2). The geometric mean plasma N-Ag levels at baseline, day 1, day 3, and day 5 in the nmAb group were 847 ng/L, 442 ng/L, 43 ng/L, and 12 ng/L, respectively; corresponding values in the placebo group were 844 ng/L, 474 ng/L, 62 ng/L, and 15 ng/L (Supplementary Table 3). After adjustment for baseline levels, the treatment group had 20% (95% CI, 12%–27%) lower plasma N-Ag levels, averaged over days 1, 3, and 5, than the placebo group (*P* < .001). The rate of decline in plasma N-Ag from baseline through day 5 was steeper in the nmAb group compared with placebo (*P* < .001 for treatment by day interaction) (Figure 1*A*). Of the 4 nmAb treatments, bamlanivimab had the smallest effect on plasma N-Ag levels; however, there was no statistically significant difference between agents (*P* = .64) (Table 2 and Supplementary Figures 1–6).

**Table 2.** Longitudinal Analysis of Change in Trajectories of Nucleocapsid Antigen, Anti-Nucleocapsid Antibody, C-Reactive Protein, Interleukin-6, and D-Dimer Associated With Neutralizing Monoclonal Antibody Treatment for Total Cohort and Trials of Individual Agents

		Plasma N-Ag (n = 2147)		Anti-N-Ab (n = 2149)		C-Reactive Protein (n = 1985)		Interleukin-6 (n = 2102)		D-Dimer (n = 2102)	
	No.	GM Ratio (95% CI)	*P* ^a^	Mean Difference (95% CI)	*P* ^a^	GM Ratio (95% CI)	*P* ^a^	GM Ratio (95% CI)	*P* ^a^	GM Ratio (95% CI)	*P* ^a^
Combined cohort	2149	0.80 (.73–.88)	<.001	−0.02 (−.11 to .07)	.412	0.96 (.89–1.03)	.470	1.03 (.93–1.13)	.011	1.03 (.99–1.08)	.979
Bamlanivimab	306	0.91 (.70–1.18)	.705	−0.17 (−.43 to .09)	.363	1.13 (.90–1.43)	.265	1.09 (.89–1.33)	.605	1.00 (.90–1.12)	.707
Sotrovimab	204	0.76 (.57–1.01)	.012	−0.05 (−.25 to .16)	.418	0.93 (.77–1.12)	.042	0.93 (.76–1.13)	.015	1.06 (.97–1.16)	.425
Amubarvimab-romlusevimab	250	0.69 (.53–.90)	<.001	0.12 (−.09 to .33)	.149	1.01 (.85–1.20)	.740	1.01 (.82–1.24)	.779	1.10 (1.01–1.20)	.661
Tixagevimab-cilgavimab	1339	0.81 (.72–.90)	<.001	−0.01 (−.12 to .09)	.412	0.93 (.85–1.02)	.470	1.03 (.91–1.17)	.011	1.04 (.99–1.10)	.979

Abbreviations: Ab, antibody; Anti-N-Ab, anti-nucleocapsid Ab as signal to cutoff ratio; CI, confidence interval; GM, geometric mean; n, number of participants with an available measurement at baseline and at least 1 follow-up sample; N-Ag, nucleocapsid antigen.

^a^ *P* value for heterogeneity of the treatment effect over time (interaction between treatment group and time; 3 categories: days 1, 3, and 5).

Anti-N Ab levels increased from baseline in both nmAb and placebo groups (Figure 1*B* and Table 2). There was no significant difference between the nmAb and placebo groups at any of the individual follow-up days 1, 3, or 5, or averaged across follow-up, in the combined cohort or in the individual trials. No interaction with the time variable was observed.

There was an expected marked increase from baseline to day 1 in anti-S Ab neutralization activity among participants receiving nmAb compared with a smaller linear increase over time in the placebo group, reflecting that all investigated nmAbs were anti-S antibodies (Figure 1*C*). The increase of anti-S Ab neutralization among participants receiving sotrovimab was lower than the increase for the other nmAbs (Supplementary Figure 3), consistent with the specific target of sotrovimab on the outside of the receptor-binding domain leading to lower detection in the assay [20].

CRP declined steadily from baseline through day 5 in both the nmAb and placebo groups, and much less pronounced declines were seen for IL-6 and D-dimer (Figure 1*D*–*F* and Table 2). There was no statistically significant difference in these biomarker levels between nmAb and placebo groups, and no evidence for interaction between treatment group and time.

**Figure 2.**
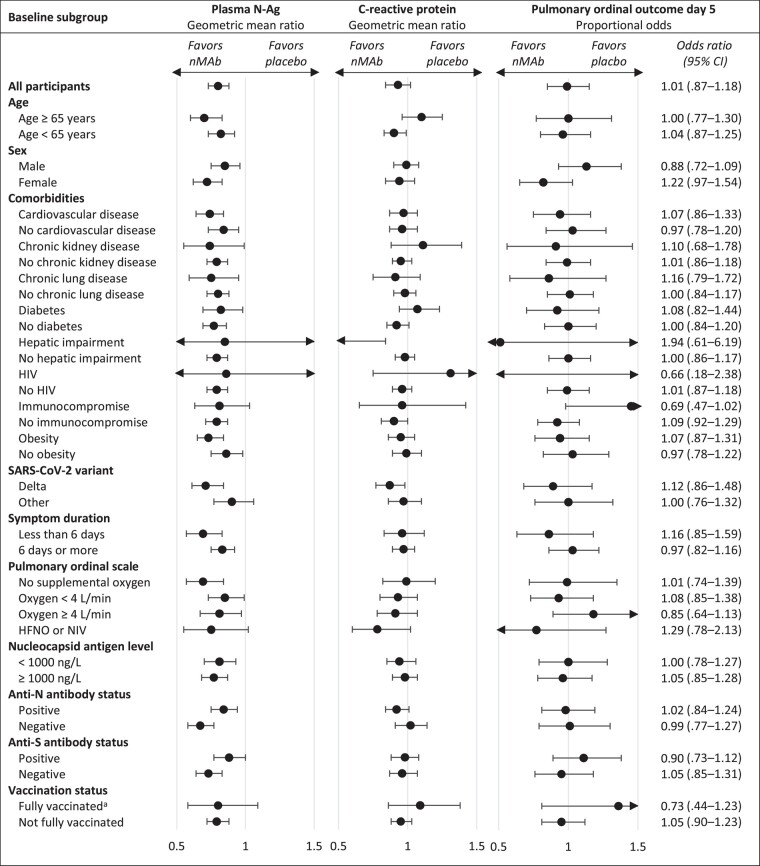
Subgroup analysis of baseline factors affected by neutralizing monoclonal antibody treatment on nucleocapsid antigen, C-reactive protein, and pulmonary ordinal outcome on day 5. Black circles represent the geometric mean ratio (plasma N-Ag, C-reactive protein) and odds ratio (pulmonary ordinal outcome) between nmAb and placebo groups with 95% CIs. Abbreviations: Anti-N, anti-nucleocapsid; Anti-S, anti-spike; CI, confidence interval; HFNO, high-flow nasal oxygen; HIV, human immunodeficiency virus; N-Ag, nucleocapsid antigen; NIV, non-invasive ventilation; nmAb, neutralizing monoclonal antibody; SARS-CoV-2, severe acute respiratory syndrome coronavirus 2. ^a^Fully vaccinated indicates full course completed, symptoms started at least 14 days after the last dose.

The longitudinal differences of laboratory measurements in the nmAb groups compared with placebo were homogeneous across subgroups defined by baseline factors (Figure 2 and Supplementary Table 4). Almost all subgroups demonstrated the same pattern of a statistically significant decrease in plasma N-Ag with nmAb compared to placebo, but no corresponding difference in other biomarker trajectories. There was no significant interaction between treatment groups and subgroup indicator for any of the outcomes (Supplementary Table 4). No impact of nmAb treatment on day 5 pulmonary ordinal scale was seen in the total cohort (common odds ratio, 1.01; 95% CI, .87–1.18), or any of the subgroups (Figure 2).

## DISCUSSION

After treatment with nmAb we observed a decline in viral burden, as measured by plasma N-Ag, with a steeper decrease from baseline through day 5 compared to placebo. These results confirm an antiviral effect of nmAb among hospitalized patients receiving remdesivir as part of the background standard of care regimen. Importantly, administration of nmAb did not mitigate the endogenous immune response reflected by anti-N Ab levels over time, a concern that has previously been raised about nmAbs directed towards the SARS-CoV-2 spike protein [21]. Interestingly, the size of the effect of nmAb on different trajectories did not vary significantly between subgroups, including participants who were anti-S Ab negative or receiving HFNO or NIV at baseline, which are subgroups where a clinical benefit of nmAb treatment has previously been reported [1, 7, 8]. Differences in plasma N-Ag trajectories between individual nmAb agents likely reflects differences in design and binding affinity as well as changes in binding sites of the virus over time.

The numerical magnitude of the change in plasma N-Ag for nmAb compared to placebo was small and there was no effect on markers of inflammation, D-dimer, or clinical improvement assessed by the pulmonary ordinal scale on day 5. This brings into question whether it has clinical or biological relevance.

Clinical progression in hospitalized patients is driven by a complex interplay between viral burden and inflammation [14–17], and our findings raise 2 possible explanations: (1) the antiviral effect of nmAbs is not sufficiently potent to add to the effect of remdesivir alone, or (2) nmAb treatment is ineffective because inflammation is the primary driver of disease progression in COVID-19 patients in need of hospitalization. To the second point, it is possible that administration of nmAb earlier in the disease course would result in decrease of inflammatory markers and improved outcome correlating with the decrease in plasma N-Ag. Future studies of treatment strategies in hospitalized patients with COVID-19 should focus on addressing these hypotheses. Perhaps an antiviral that is more potent or has a different mechanism of action than nmAbs will have more convincing clinical effects. On the other hand, more effective immunomodulatory strategies may lead to less deleterious effects of uncontrolled inflammatory responses.

Our analysis has both strengths and limitations. The analysis of trajectories over well-defined time points adds dynamic granularity to previous evidence based mostly on baseline measurements. The randomized comparison versus a placebo group minimized confounding and provides causal evidence. The large number of international sites provided comprehensive representation of different populations. An important limitation is that using plasma N-Ag concentrations may not be a specific proxy for actual ongoing viral replication. However, previously published data from the same cohort showed clear correlation between baseline plasma N-Ag and both baseline disease severity and clinical outcomes, which strongly supports the use of this biomarker as a measure of viral burden in this population of hospitalized patients [22]. Pooling of data from 4 different nmAb agents is also a limitation because this assumes similar properties, and it is plausible that there were relevant differences in trajectories among the different agents. The universal use of the antiviral remdesivir could also have influenced the estimate of the nmAb treatment effect. It is not clear whether our findings are generalizable to contemporary patients with high prevalence of vaccination and infected with Omicron sublineages. Finally, analyses are exploratory because we considered many outcomes without adjustment for inflation of type 1 error.

In summary, this study represents a placebo-matched comparison of virologic and immunologic response to nmAb in over 2000 hospitalized patients. Despite confirming an expected virological response to nmAb, we did not demonstrate any corresponding improvement of early pulmonary status suggesting meaningful clinical benefit of this drug class in hospitalized patients with COVID-19. Importantly, we did not observe blunting of the endogenous humoral response or difference in the inflammatory response, which is reassuring when nmAbs are considered for emerging SARS-CoV-2 variants of concerns or future epidemics with novel pathogens. Important questions on the roles of additional antiviral and immunomodulatory treatments remain and should be addressed by clinical trials in patients hospitalized with COVID-19.

## Supplementary Data

Supplementary materials are available at *The Journal of Infectious Diseases* online (http://jid.oxfordjournals.org/). Supplementary materials consist of data provided by the author that are published to benefit the reader. The posted materials are not copyedited. The contents of all supplementary data are the sole responsibility of the authors. Questions or messages regarding errors should be addressed to the author.

## Supplementary Material

jiad446_Supplementary_Data

## Notes

***Author contributions.*** T. J., G. G., M. J., T. M., B. G., M. M., and E. K. conceived of the study. All authors participated in its design and interpretation of results. G. G. led the statistical analysis. T. J. drafted the manuscript, and all other authors contributed to revisions. All authors read and approved the final manuscript.

***Financial support*.** This research was, in part, funded by the National Institutes of Health (NIH) Agreement 1OT2HL156812-01. The trial was sponsored and primarily funded by the National Institute of Allergy and Infectious Diseases (NIAID), National Institutes of Health (NIH), Bethesda, MD., in part with federal funds from the NIAID and the National Cancer Institute, NIH, under contract 75N91019D00024, task order numbers 75N91020F00014 and 75N91020F00039.
