# Supplementary material for: Effect of Neutralizing Monoclonal Antibody Treatment on Early Trajectories of Virologic and Immunologic Biomarkers in Patients Hospitalized With COVID-19
Source: J Infect Dis. 2023 Nov 9;229(3):671–9. doi: 10.1093/infdis/jiad446 (PMC10938202; doi:10.1093/infdis/jiad446)

**Figure S2. Line plots of the mean anti-nucleocapsid antibody (anti-N-Ab) levels (with 95% CI) over time by neutralizing monoclonal antibody (nMAb) treatment and placebo groups. One panel is presented for each of the 4 nMAb trials.**


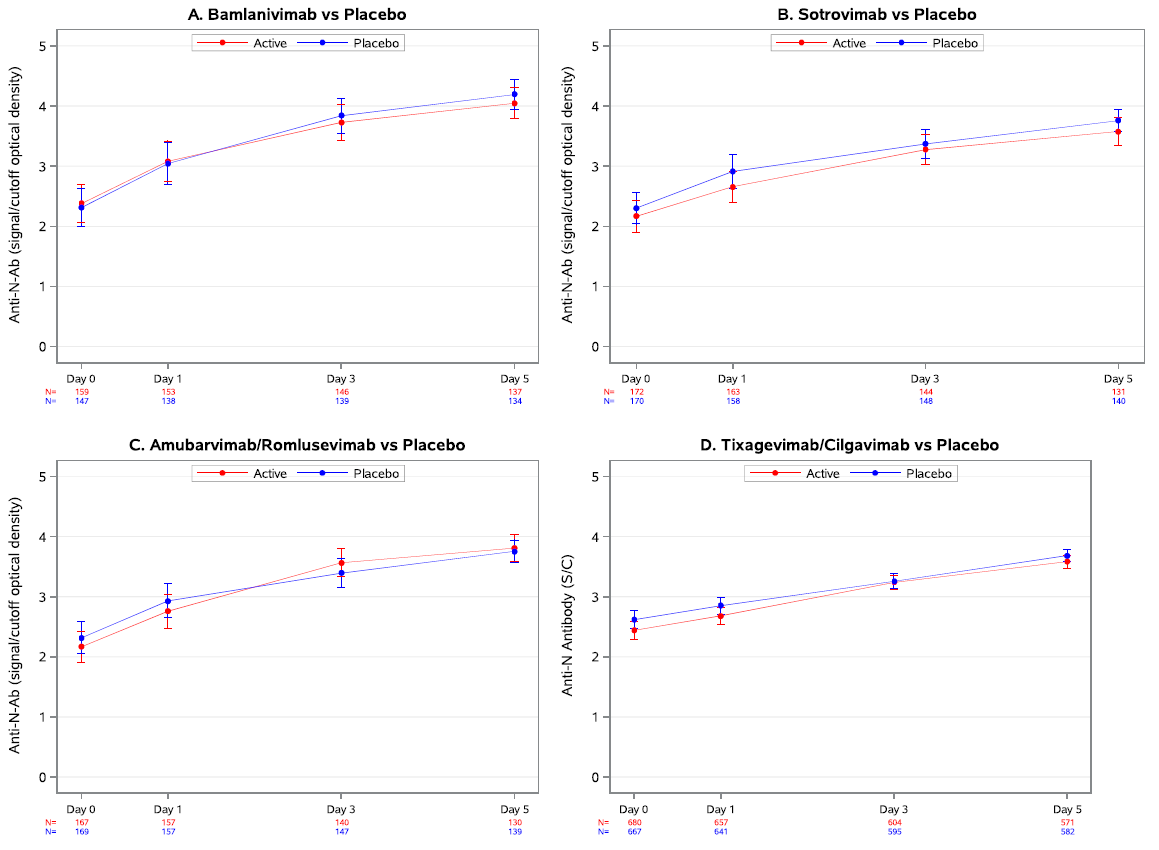

Supplement: jiad446_Supplementary_Data [file jiad446_supplementary_data.zip › TICO-trajectories-20230929-figS2-anti-N-line-plots.docx]
