# Supplementary material for: Effect of Neutralizing Monoclonal Antibody Treatment on Early Trajectories of Virologic and Immunologic Biomarkers in Patients Hospitalized With COVID-19
Source: J Infect Dis. 2023 Nov 9;229(3):671–9. doi: 10.1093/infdis/jiad446 (PMC10938202; doi:10.1093/infdis/jiad446)
Supplement: jiad446_Supplementary_Data [file jiad446_supplementary_data.zip › TICO-trajectories-20230929-tabS1-comorbidities.docx]

**Table S1. Definitions of baseline comorbidities.**

| **Comorbidities** |  |
| --- | --- |
| Cardiovascular disease | - Cerebrovascular event (thrombotic or hemorrhagic) - Heart failure - Hypertension requiring medications - Myocardial infarction (MI) or other acute coronary syndrome |
| Chronic kidney disease | - Renal impairment - Renal replacement therapy prior to COVID-19 |
| Chronic lung disease | - Asthma - Chronic obstructive lung disease - Chronic continuous oxygen supplementation |
| Diabetes | - Diabetes mellitus requiring medication |
| Hepatic impairment | - Hepatic impairment |
| HIV | - HIV |
| Immunocompromise | - Immunosuppressive disorder other than HIV - Malignancy (active or receiving treatment) - Treatment with antirejection medications (antirejection medicine after solid or stem cell transplant) - Treatment with biological medications (Biological medicine to treat autoimmune disease or cancer) - Treatment with immune modulators (Interleukin-1 inhibitors, Interleukin-6 inhibitors, Interferons, Janus kinase inhibitors, TNF inhibitors, others) |
| Obesity | - Body mass index of 30 kg/m^2^ or above |
