# Supplementary material for: Effect of Neutralizing Monoclonal Antibody Treatment on Early Trajectories of Virologic and Immunologic Biomarkers in Patients Hospitalized With COVID-19
Source: J Infect Dis. 2023 Nov 9;229(3):671–9. doi: 10.1093/infdis/jiad446 (PMC10938202; doi:10.1093/infdis/jiad446)
Supplement: jiad446_Supplementary_Data [file jiad446_supplementary_data.zip › TICO-trajectories-20230929-tabS2-pulmonary-ordinal-scale.docx]

**Table S2. The 7-category pulmonary ordinal outcome scale.**

| 1. Can independently undertake usual activities with minimal or no symptoms  2. Symptomatic and currently unable to independently undertake usual activities but no need of supplemental oxygen (or not above premorbid requirements)  3. Supplemental oxygen (<4 liters/min, or <4 liters/min above premorbid requirements)  4. Supplemental oxygen (≥4 liters/min, or ≥4 liters/min above premorbid requirements, but not high-flow oxygen)  5. Non-invasive ventilation or high-flow oxygen (high flow nasal cannula)  6. Invasive ventilation, extracorporeal membrane oxygenation (ECMO), mechanical circulatory support, or new receipt of renal replacement therapy  7. Death |
| --- |
