# Supplementary material for: Effect of Neutralizing Monoclonal Antibody Treatment on Early Trajectories of Virologic and Immunologic Biomarkers in Patients Hospitalized With COVID-19
Source: J Infect Dis. 2023 Nov 9;229(3):671–9. doi: 10.1093/infdis/jiad446 (PMC10938202; doi:10.1093/infdis/jiad446)
Supplement: jiad446_Supplementary_Data [file jiad446_supplementary_data.zip › TICO-trajectories-20230929-tabS3-follow-up-estimates.docx]

**Table S3. Geometric means (for plasma nucleocapsid antigen [N-Ag], C-reactive protein, Interleukin-6, and D-dimer) and means (for anti-N Ab [anti-nucleocapsid antibody] and anti-S Ab (anti-spike antibody]) along with 95% confidence intervals (CI) in participants randomized to neutralizing monoclonal antibody (nMAb) or placebo.**

|  | **Trial treatment allocation** | | | |
| --- | --- | --- | --- | --- |
|  | nMAb | | Placebo | |
| **Plasma N-Ag** | N | Geometric mean  (95% CI) | N | Geometric mean  (95% CI) |
| Baseline | 1178 | 847 [741, 969] | 969 | 845 [729, 978] |
| Day 1 | 1129 | 442 [381, 514] | 923 | 475 [405, 558] |
| Day 3 | 1033 | 43 [38, 49] | 869 | 62 [54, 72] |
| Day 5 | 970 | 12 [11, 13] | 844 | 15 [14, 17] |
| **Anti-N Ab** | N | Mean  (95% CI) | N | Mean  (95% CI) |
| Baseline | 1178 | 2.4 [2.2, 2.5] | 971 | 2.5 [2.4, 2.6] |
| Day 1 | 1130 | 2.7 [2.6, 2.9] | 925 | 2.9 [2.8, 3.0] |
| Day 3 | 1034 | 3.4 [3.3, 3.5] | 870 | 3.4 [3.3, 3.5] |
| Day 5 | 969 | 3.7 [3.6, 3.8] | 844 | 3.8 [3.7, 3.9] |
| **Anti-S Ab** | N | Mean  (95% CI) | N | Mean  (95% CI) |
| Baseline | 1172 | 38.5 [36.5, 40.5] | 961 | 38.2 [36.1, 40.4] |
| Day 1 | 1122 | 92.1 [91.2, 92.9] | 914 | 50.0 [47.9, 52.1] |
| Day 3 | 1027 | 93.7 [93.0, 94.4] | 861 | 65.9 [63.9, 67.9] |
| Day 5 | 965 | 94.3 [93.6, 95.0] | 833 | 77.0 [75.3, 78.7] |
| **C-reactive protein** | N | Geometric mean  (95% CI) | N | Geometric mean  (95% CI) |
| Baseline | 1095 | 26.9 [25.2, 28.6] | 890 | 25.4 [23.6, 27.3] |
| Day 1 | 1043 | 17.9 [16.7, 19.3] | 849 | 17.3 [16.0, 18.6] |
| Day 3 | 949 | 10.2 [9.4, 11.1] | 795 | 10.7 [9.9, 11.6] |
| Day 5 | 898 | 6.8 [6.2, 7.5] | 769 | 6.9 [6.3, 7.6] |
| **Interleukin-6** | N | Geometric mean  (95% CI) | N | Geometric mean  (95% CI) |
| Baseline | 1152 | 6.3 [5.8, 6.8] | 950 | 5.9 [5.4, 6.4] |
| Day 1 | 1097 | 6.5 [5.9, 7.1] | 905 | 5.6 [5.1, 6.1] |
| Day 3 | 1003 | 5.4 [4.8, 5.9] | 854 | 5.7 [5.1, 6.4] |
| Day 5 | 943 | 5.0 [4.5, 5.6] | 822 | 4.7 [4.2, 5.3] |
| **D-dimer** | N | Geometric mean  (95% CI) | N | Geometric mean  (95% CI) |
| Baseline | 1152 | 1.01 [0.97, 1.05] | 950 | 1.02 [0.97, 1.07] |
| Day 1 | 1097 | 0.95 [0.91, 0.99] | 905 | 0.94 [0.89, 0.99] |
| Day 3 | 1003 | 0.90 [0.86, 0.95] | 853 | 0.88 [0.83, 0.93] |
| Day 5 | 942 | 0.88 [0.83, 0.93] | 821 | 0.85 [0.79, 0.90] |
