# Supplementary material for: Effect of Neutralizing Monoclonal Antibody Treatment on Early Trajectories of Virologic and Immunologic Biomarkers in Patients Hospitalized With COVID-19
Source: J Infect Dis. 2023 Nov 9;229(3):671–9. doi: 10.1093/infdis/jiad446 (PMC10938202; doi:10.1093/infdis/jiad446)
Supplement: jiad446_Supplementary_Data [file jiad446_supplementary_data.zip › TICO-trajectories-20230929-tabS4-subgroups.docx]

**Table S4. Longitudinal analysis of neutralizing monoclonal antibody treatment effect on day 0-5 trajectories of nucleocapsid antigen, anti-N antibody, C-reactive protein, Interleukin-6, and D-dimer, according to subgroups defined by baseline factors.**

|  | **Nucleocapsid antigen** | | **Anti-nucleocapsid antibody** | | **C-reactive protein** | | **Interleukin-6** | | **D-dimer** | |
| --- | --- | --- | --- | --- | --- | --- | --- | --- | --- | --- |
| **Baseline subgroup*** | N | Geometric mean ratio [95% CI] | N | Mean difference  [95% CI] | N | Geometric mean ratio [95% CI] | N | Geometric mean ratio [95% CI] | N | Geometric mean ratio [95% CI] |
| Age ≥ 65 years | 698 | 0.70 [0.60, 0.83] | 698 | 0.05 [-0.10, 0.21] | 635 | 1.10 [0.96, 1.25] | 684 | 0.98 [0.82, 1.16] | 684 | 1.06 [0.98, 1.14] |
| Age < 65 years | 1,449 | 0.82 [0.73, 0.92] | 1,451 | -0.03 [-0.13, 0.08] | 1,350 | 0.90 [0.83, 0.99] | 1,418 | 1.00 [0.89, 1.13] | 1,418 | 1.02 [0.96, 1.07] |
| P-value |  | 0.138 |  | 0.391 |  | 0.014 |  | 0.827 |  | 0.395 |
| Male | 1,244 | 0.85 [0.75, 0.96] | 1,246 | 0.03 [-0.09, 0.14] | 1,149 | 0.99 [0.90, 1.08] | 1,218 | 1.06 [0.93, 1.21] | 1,218 | 1.04 [0.98, 1.10] |
| Female | 903 | 0.72 [0.62, 0.83] | 903 | -0.06 [-0.19, 0.07] | 836 | 0.94 [0.84, 1.05] | 884 | 0.93 [0.80, 1.08] | 884 | 1.03 [0.96, 1.10] |
| P-value |  | 0.101 |  | 0.323 |  | 0.519 |  | 0.175 |  | 0.847 |
| Cardiovascular disease | 1,033 | 0.74 [0.64, 0.84] | 1,033 | 0.02 [-0.10, 0.15] | 948 | 0.97 [0.87, 1.07] | 1,006 | 1.04 [0.90, 1.19] | 1,006 | 1.06 [1.00, 1.13] |
| No cardiovascular disease | 1,114 | 0.84 [0.73, 0.95] | 1,116 | -0.03 [-0.16, 0.09] | 1,037 | 0.96 [0.87, 1.07] | 1,096 | 0.97 [0.85, 1.11] | 1,096 | 1.00 [0.94, 1.07] |
| P-value |  | 0.189 |  | 0.571 |  | 0.954 |  | 0.496 |  | 0.181 |
| Chronic kidney disease | 214 | 0.74 [0.55, 0.99] | 211 | -0.00 [-0.28, 0.28] | 196 | 1.11 [0.88, 1.39] | 205 | 1.23 [0.90, 1.68] | 205 | 1.08 [0.94, 1.24] |
| No chronic kidney disease | 1,936 | 0.79 [0.72, 0.87] | 1,938 | -0.01 [-0.10, 0.08] | 1,789 | 0.95 [0.89, 1.03] | 1,897 | 0.98 [0.88, 1.09] | 1,897 | 1.03 [0.98, 1.08] |
| P-value |  | 0.638 |  | 0.951 |  | 0.221 |  | 0.178 |  | 0.547 |
| Chronic lung disease | 325 | 0.75 [0.59, 0.95] | 326 | -0.01 [-0.24, 0.21] | 303 | 0.91 [0.75, 1.09] | 316 | 0.90 [0.70, 1.16] | 316 | 1.06 [0.95, 1.18] |
| No chronic lung disease | 1,822 | 0.80 [0.72, 0.88] | 1,823 | -0.01 [-0.10, 0.09] | 1,682 | 0.98 [0.90, 1.06] | 1,786 | 1.02 [0.92, 1.13] | 1,786 | 1.03 [0.98, 1.08] |
| P-value |  | 0.633 |  | 0.970 |  | 0.473 |  | 0.384 |  | 0.666 |
| Diabetes | 618 | 0.82 [0.69, 0.98] | 618 | 0.00 [-0.16, 0.17] | 568 | 1.07 [0.94, 1.23] | 601 | 1.06 [0.88, 1.27] | 601 | 1.06 [0.98, 1.15] |
| No diabetes | 1,529 | 0.77 [0.69, 0.86] | 1,531 | -0.01 [-0.11, 0.09] | 1,417 | 0.92 [0.85, 1.01] | 1,501 | 0.98 [0.87, 1.09] | 1,501 | 1.02 [0.97, 1.08] |
| P-value |  | 0.579 |  | 0.903 |  | 0.068 |  | 0.457 |  | 0.458 |
| Hepatic impairment | 36 | 0.85 [0.40, 1.78] | 36 | -0.27 [-0.96, 0.41] | 36 | 0.49 [0.28, 0.84] | 36 | 0.75 [0.35, 1.60] | 36 | 0.80 [0.57, 1.12] |
| No hepatic impairment | 2,111 | 0.79 [0.72, 0.87] | 2,113 | -0.00 [-0.09, 0.08] | 1,949 | 0.98 [0.91, 1.05] | 2,066 | 1.01 [0.91, 1.11] | 2,066 | 1.04 [0.99, 1.09] |
| P-value |  | 0.855 |  | 0.449 |  | 0.014 |  | 0.454 |  | 0.136 |
| HIV | 36 | 0.86 [0.41, 1.80] | 36 | -0.35 [-1.04, 0.33] | 35 | 1.31 [0.75, 2.29] | 35 | 0.98 [0.45, 2.13] | 35 | 1.01 [0.71, 1.43] |
| No HIV | 2,111 | 0.79 [0.72, 0.87] | 2,113 | -0.00 [-0.09, 0.09] | 1,950 | 0.96 [0.89, 1.03] | 2,067 | 1.00 [0.91, 1.10] | 2,067 | 1.03 [0.99, 1.08] |
| P-value |  | 0.832 |  | 0.321 |  | 0.279 |  | 0.959 |  | 0.886 |
| Immunocompromise | 328 | 0.81 [0.63, 1.03] | 328 | -0.05 [-0.27, 0.17] | 312 | 0.96 [0.65, 1.42] | 321 | 1.23 [0.96, 1.57] | 321 | 1.07 [0.96, 1.20] |
| No immunocompromise | 1,819 | 0.79 [0.71, 0.87] | 1,821 | 0.00 [-0.09, 0.09] | 1,673 | 0.90 [0.81, 1.00] | 1,781 | 0.97 [0.87, 1.07] | 1,781 | 1.03 [0.98, 1.08] |
| P-value |  | 0.849 |  | 0.667 |  | 0.483 |  | 0.080 |  | 0.458 |
| Obesity | 1,150 | 0.73 [0.65, 0.84] | 1,151 | 0.02 [-0.09, 0.14] | 1,065 | 0.95 [0.86, 1.05] | 1,122 | 1.02 [0.89, 1.16] | 1,122 | 1.04 [0.98, 1.10] |
| No obesity | 991 | 0.86 [0.75, 0.98] | 992 | -0.04 [-0.17, 0.09] | 915 | 0.99 [0.89, 1.10] | 974 | 0.98 [0.85, 1.13] | 974 | 1.02 [0.96, 1.09] |
| P-value |  | 0.109 |  | 0.475 |  | 0.591 |  | 0.715 |  | 0.731 |
| Delta | 656 | 0.71 [0.60, 0.85] | 658 | -0.03 [-0.18, 0.13] | 641 | 0.87 [0.77, 0.98] | 641 | 1.02 [0.86, 1.21] | 641 | 1.01 [0.94, 1.09] |
| Other | 1,474 | 0.84 [0.75, 0.94] | 1,474 | -0.02 [-0.12, 0.09] | 1,328 | 1.02 [0.93, 1.11] | 1,445 | 1.02 [0.91, 1.15] | 1,445 | 1.04 [0.99, 1.10] |
| P-value |  | 0.132 |  | 0.917 |  | 0.043 |  | 0.995 |  | 0.493 |
| Symptom duration < 6 days | 507 | 0.69 [0.57, 0.83] | 507 | -0.04 [-0.21, 0.14] | 457 | 0.96 [0.83, 1.12] | 497 | 0.92 [0.75, 1.12] | 497 | 1.08 [0.98, 1.18] |
| Symptom duration ≥ 6 days | 1,640 | 0.83 [0.75, 0.92] | 1,642 | -0.01 [-0.11, 0.09] | 1,528 | 0.97 [0.89, 1.05] | 1,605 | 1.03 [0.92, 1.15] | 1,605 | 1.02 [0.97, 1.07] |
| P-value |  | 0.093 |  | 0.773 |  | 0.893 |  | 0.326 |  | 0.323 |
| 2: No supplemental oxygen | 553 | 0.69 [0.57, 0.84] | 553 | 0.06 [-0.12, 0.23] | 511 | 0.99 [0.82, 1.20] | 545 | 1.18 [0.90, 1.54] | 545 | 1.03 [0.91, 1.15] |
| 3: Oxygen < 4 L/min | 815 | 0.85 [0.73, 0.99] | 816 | 0.05 [-0.09, 0.19] | 748 | 0.93 [0.80, 1.07] | 800 | 0.90 [0.73, 1.11] | 800 | 1.04 [0.95, 1.14] |
| 4: Oxygen ≥ 4 L/min | 582 | 0.81 [0.67, 0.97] | 582 | -0.09 [-0.26, 0.07] | 553 | 0.91 [0.78, 1.07] | 567 | 1.07 [0.85, 1.35] | 567 | 0.98 [0.89, 1.09] |
| 5: HFNO or NIV | 197 | 0.75 [0.55, 1.02] | 198 | -0.04 [-0.32, 0.24] | 173 | 0.78 [0.60, 1.02] | 190 | 1.00 [0.68, 1.46] | 190 | 1.13 [0.96, 1.33] |
| P-value |  | 0.418 |  | 0.536 |  | 0.462 |  | 0.826 |  | 0.568 |
| Plasma N-Ag < 1,000 ng/L | 899 | 0.81 [0.70, 0.93] | 899 | -0.07 [-0.21, 0.06] | 829 | 0.94 [0.85, 1.06] | 879 | 1.04 [0.90, 1.21] | 879 | 1.06 [0.99, 1.13] |
| Plasma N-Ag ≥ 1,000 ng/L | 1,248 | 0.77 [0.68, 0.87] | 1,249 | 0.03 [-0.08, 0.15] | 1,156 | 0.98 [0.89, 1.07] | 1,223 | 0.97 [0.85, 1.10] | 1,223 | 1.02 [0.96, 1.08] |
| P-value |  | 0.624 |  | 0.235 |  | 0.646 |  | 0.491 |  | 0.408 |
| Anti-N Ab positive | 1,329 | 0.84 [0.75, 0.94] | 1,330 | -0.06 [-0.16, 0.04] | 1,229 | 0.92 [0.84, 1.01] | 1,299 | 1.04 [0.92, 1.17] | 1,299 | 1.05 [1.00, 1.11] |
| Anti-N Ab negative | 818 | 0.67 [0.58, 0.77] | 819 | 0.08 [-0.05, 0.22] | 756 | 1.02 [0.91, 1.14] | 803 | 0.93 [0.79, 1.09] | 803 | 1.00 [0.93, 1.08] |
| P-value |  | 0.016 |  | 0.093 |  | 0.197 |  | 0.289 |  | 0.289 |
| Anti-S Ab positive | 1,063 | 0.88 [0.77, 1.00] | 1,065 | 0.03 [-0.10, 0.15] | 988 | 0.98 [0.88, 1.08] | 1,037 | 1.06 [0.93, 1.22] | 1,037 | 1.10 [1.03, 1.17] |
| Anti-S Ab negative | 1,083 | 0.73 [0.64, 0.83] | 1,083 | -0.05 [-0.17, 0.07] | 996 | 0.96 [0.87, 1.07] | 1,064 | 0.95 [0.83, 1.09] | 1,064 | 0.98 [0.92, 1.04] |
| P-value |  | 0.042 |  | 0.385 |  | 0.828 |  | 0.254 |  | 0.010 |
| Fully vaccinated† | 189 | 0.80 [0.58, 1.09] | 190 | 0.36 [0.07, 0.64] | 183 | 1.09 [0.86, 1.38] | 183 | 0.97 [0.70, 1.35] | 183 | 1.03 [0.89, 1.20] |
| Not fully vaccinated | 1,958 | 0.79 [0.72, 0.88] | 1,959 | -0.05 [-0.14, 0.04] | 1,802 | 0.95 [0.88, 1.03] | 1,919 | 1.01 [0.91, 1.11] | 1,919 | 1.03 [0.99, 1.08] |
| P-value |  | 0.973 |  | 0.007 |  | 0.294 |  | 0.833 |  | 0.986 |

Abbreviations: Anti-N Ab, anti-nucleocapsid antibody; Anti-S Ab, anti-spike antibody; HFNO, high-flow nasal oxygen; NIV, non-invasive ventilation; N-Ag, nucleocapsid antigen.
*P-value for interaction between subgroup and treatment group.
†Fully vaccinated = full course completed, symptoms started at least 14 days after the last dose.
