# Supplementary material for: Effect of Neutralizing Monoclonal Antibody Treatment on Early Trajectories of Virologic and Immunologic Biomarkers in Patients Hospitalized With COVID-19
Source: J Infect Dis. 2023 Nov 9;229(3):671–9. doi: 10.1093/infdis/jiad446 (PMC10938202; doi:10.1093/infdis/jiad446)
Supplement: jiad446_Supplementary_Data [file jiad446_supplementary_data.zip › TICO-trajectories-20230929-tabS5-study-group-members.docx]

**Table S5: List of all study group members.**

| **Last name** | **First name** | **Degrees** | **Affiliation** |
| --- | --- | --- | --- |
| Sahner | David | M.D. | U.S. National Institute of Allergy and Infectious Diseases incl. Department of Clinical Research |
| Tierney | John | B.Sc.N. | U.S. National Institute of Allergy and Infectious Diseases incl. Department of Clinical Research |
| Vogel | Susan E. | R.N., B.Sc.N. | U.S. National Institute of Allergy and Infectious Diseases incl. Department of Clinical Research |
| Herpin | Betsey R. | M.Sc.N., C.C.R.C., R.N. | U.S. National Institute of Allergy and Infectious Diseases incl. Department of Clinical Research |
| Smolskis | Mary C. | B.Sc.N., M.A | U.S. National Institute of Allergy and Infectious Diseases incl. Department of Clinical Research |
| McKay | Laura A. | M.SC. | U.S. National Institute of Allergy and Infectious Diseases incl. Department of Clinical Research |
| Cahill | Kelly | R.N., M.Sc., C.C.R.C., R.A.C. | U.S. National Institute of Allergy and Infectious Diseases incl. Department of Clinical Research |
| Crew | Page | PharmD., M.P.H., B.C.P.S. | U.S. National Institute of Allergy and Infectious Diseases incl. Department of Clinical Research |
| Sardana | Ratna | B.A. | U.S. National Institute of Allergy and Infectious Diseases incl. Department of Clinical Research |
| Raim | Sharon Segal | M.P.H. | U.S. National Institute of Allergy and Infectious Diseases incl. Department of Clinical Research |
| Hensely | Lisa | Ph.D. | U.S. National Institute of Allergy and Infectious Diseases incl. Department of Clinical Research |
| Lorenzo | Johsua | M.P.H. | U.S. National Institute of Allergy and Infectious Diseases incl. Department of Clinical Research |
| Mock | Rebecca | Ph.D., R.A.C. | U.S. National Institute of Allergy and Infectious Diseases incl. Department of Clinical Research |
| Zuckerman | Judith | B.S.N. | U.S. National Institute of Allergy and Infectious Diseases incl. Department of Clinical Research |
| Atri | Negin | M.P.H. | U.S. National Institute of Allergy and Infectious Diseases incl. Department of Clinical Research |
| Miller | Mark | PharmD., B.C.P.S., R.A.C. | U.S. National Institute of Allergy and Infectious Diseases incl. Department of Clinical Research |
| Vallee | David | PharmD., M.P.H. | U.S. National Institute of Allergy and Infectious Diseases incl. Department of Clinical Research |
| Chung | Lucy | PharmD., C.C.R.P. | U.S. National Institute of Allergy and Infectious Diseases incl. Department of Clinical Research |
| Kang | Nayon | PharmD., M.S. | U.S. National Institute of Allergy and Infectious Diseases incl. Department of Clinical Research |
| Barrett | Kevin | R.N., B.Sc.N. | U.S. National Institute of Allergy and Infectious Diseases incl. Department of Clinical Research |
| Adam | Stacey J. | Ph.D. | Foundation for the National Institutes of Health, The Accelerating COVID-19 Therapeutic Interventions and Vaccines (ACTIV) and Operation Warp Speed |
| Read | Sarah | M.D. | Foundation for the National Institutes of Health, The Accelerating COVID-19 Therapeutic Interventions and Vaccines (ACTIV) and Operation Warp Speed |
| Draghia-Akli | Ruxandra | M.D., Ph.D. | Foundation for the National Institutes of Health, The Accelerating COVID-19 Therapeutic Interventions and Vaccines (ACTIV) and Operation Warp Speed |
| Currier | Judy | M.D. | Foundation for the National Institutes of Health, The Accelerating COVID-19 Therapeutic Interventions and Vaccines (ACTIV) and Operation Warp Speed |
| Hughes | Eric | M.D., Ph.D. | Foundation for the National Institutes of Health, The Accelerating COVID-19 Therapeutic Interventions and Vaccines (ACTIV) and Operation Warp Speed |
| Harrigan | Rachel H. | M.D. | Foundation for the National Institutes of Health, The Accelerating COVID-19 Therapeutic Interventions and Vaccines (ACTIV) and Operation Warp Speed |
| Amos | Laura |  | INSIGHT SDMC, Division of Biostatistics, School of Public Health and School of Statistics, University of Minnesota, Minneapolis, MN, USA |
| Carlsen | Amy | R.N. | INSIGHT SDMC, Division of Biostatistics, School of Public Health and School of Statistics, University of Minnesota, Minneapolis, MN, USA |
| Carter | Anita |  | INSIGHT SDMC, Division of Biostatistics, School of Public Health and School of Statistics, University of Minnesota, Minneapolis, MN, USA |
| Collins | Gary | M.S. | INSIGHT SDMC, Division of Biostatistics, School of Public Health and School of Statistics, University of Minnesota, Minneapolis, MN, USA |
| Davis | Bionca | M.P.H. | INSIGHT SDMC, Division of Biostatistics, School of Public Health and School of Statistics, University of Minnesota, Minneapolis, MN, USA |
| Denning | Eileen | M.P.H. | INSIGHT SDMC, Division of Biostatistics, School of Public Health and School of Statistics, University of Minnesota, Minneapolis, MN, USA |
| DuChene | Alain |  | INSIGHT SDMC, Division of Biostatistics, School of Public Health and School of Statistics, University of Minnesota, Minneapolis, MN, USA |
| Eckroth | Kate | M.P.H. | INSIGHT SDMC, Division of Biostatistics, School of Public Health and School of Statistics, University of Minnesota, Minneapolis, MN, USA |
| Engen | Nicole | M.S. | INSIGHT SDMC, Division of Biostatistics, School of Public Health and School of Statistics, University of Minnesota, Minneapolis, MN, USA |
| Frase | Alex |  | INSIGHT SDMC, Division of Biostatistics, School of Public Health and School of Statistics, University of Minnesota, Minneapolis, MN, USA |
| Gandits | Greg | M.S. | INSIGHT SDMC, Division of Biostatistics, School of Public Health and School of Statistics, University of Minnesota, Minneapolis, MN, USA |
| Grund | Birgit | Ph.D. | INSIGHT SDMC, Division of Biostatistics, School of Public Health and School of Statistics, University of Minnesota, Minneapolis, MN, USA |
| Harrison | Merrie |  | INSIGHT SDMC, Division of Biostatistics, School of Public Health and School of Statistics, University of Minnesota, Minneapolis, MN, USA |
| Hurlbut | Nancy |  | INSIGHT SDMC, Division of Biostatistics, School of Public Health and School of Statistics, University of Minnesota, Minneapolis, MN, USA |
| Kaiser | Payton |  | INSIGHT SDMC, Division of Biostatistics, School of Public Health and School of Statistics, University of Minnesota, Minneapolis, MN, USA |
| Koopmeiners | Joseph | Ph.D. | INSIGHT SDMC, Division of Biostatistics, School of Public Health and School of Statistics, University of Minnesota, Minneapolis, MN, USA |
| Larson | Gregg | M.A. | INSIGHT SDMC, Division of Biostatistics, School of Public Health and School of Statistics, University of Minnesota, Minneapolis, MN, USA |
| Meger | Sue |  | INSIGHT SDMC, Division of Biostatistics, School of Public Health and School of Statistics, University of Minnesota, Minneapolis, MN, USA |
| Mistry | Shweta Sharma | M.S. | INSIGHT SDMC, Division of Biostatistics, School of Public Health and School of Statistics, University of Minnesota, Minneapolis, MN, USA |
| Murray | Thomas | Ph.D. | INSIGHT SDMC, Division of Biostatistics, School of Public Health and School of Statistics, University of Minnesota, Minneapolis, MN, USA |
| Nelson | Ray | R.N. | INSIGHT SDMC, Division of Biostatistics, School of Public Health and School of Statistics, University of Minnesota, Minneapolis, MN, USA |
| Quan | Kien | M.S. | INSIGHT SDMC, Division of Biostatistics, School of Public Health and School of Statistics, University of Minnesota, Minneapolis, MN, USA |
| Quan | Siu Fun |  | INSIGHT SDMC, Division of Biostatistics, School of Public Health and School of Statistics, University of Minnesota, Minneapolis, MN, USA |
| Reilly | Cavan | Ph.D. | INSIGHT SDMC, Division of Biostatistics, School of Public Health and School of Statistics, University of Minnesota, Minneapolis, MN, USA |
| Siegel | Lianne | Ph.D. | INSIGHT SDMC, Division of Biostatistics, School of Public Health and School of Statistics, University of Minnesota, Minneapolis, MN, USA |
| Thompson | Greg |  | INSIGHT SDMC, Division of Biostatistics, School of Public Health and School of Statistics, University of Minnesota, Minneapolis, MN, USA |
| Vock | David | Ph.D. | INSIGHT SDMC, Division of Biostatistics, School of Public Health and School of Statistics, University of Minnesota, Minneapolis, MN, USA |
| Walski | Jamie | M.H.A. | INSIGHT SDMC, Division of Biostatistics, School of Public Health and School of Statistics, University of Minnesota, Minneapolis, MN, USA |
| Gelijns | Annetine C. | Ph.D. | Cardiothoracic Surgical Trials Network (CTSN) International Coordinating Center (ICC). Icahn School of Medicine at Mount Sinai, New York, USA |
| Moskowitz | Alan J. | M.D. | Cardiothoracic Surgical Trials Network (CTSN) International Coordinating Center (ICC). Icahn School of Medicine at Mount Sinai, New York, USA |
| Bagiella | Emilia | Ph.D. | Cardiothoracic Surgical Trials Network (CTSN) International Coordinating Center (ICC). Icahn School of Medicine at Mount Sinai, New York, USA |
| Moquete | Ellen | R.N., B.S.N. | Cardiothoracic Surgical Trials Network (CTSN) International Coordinating Center (ICC). Icahn School of Medicine at Mount Sinai, New York, USA |
| O'Sullivan | Karen | M.P.H. | Cardiothoracic Surgical Trials Network (CTSN) International Coordinating Center (ICC). Icahn School of Medicine at Mount Sinai, New York, USA |
| Marks | Mary E. | R.N., B.S.N. | Cardiothoracic Surgical Trials Network (CTSN) International Coordinating Center (ICC). Icahn School of Medicine at Mount Sinai, New York, USA |
| Accardi | Evan | B.A. | Cardiothoracic Surgical Trials Network (CTSN) International Coordinating Center (ICC). Icahn School of Medicine at Mount Sinai, New York, USA |
| Kinzel | Emily | M.P.H. | Cardiothoracic Surgical Trials Network (CTSN) International Coordinating Center (ICC). Icahn School of Medicine at Mount Sinai, New York, USA |
| Burris | Sarah | M.H.A. | Cardiothoracic Surgical Trials Network (CTSN) International Coordinating Center (ICC). Icahn School of Medicine at Mount Sinai, New York, USA |
| Bedoya | Gabriela | B.S: | Cardiothoracic Surgical Trials Network (CTSN) International Coordinating Center (ICC). Icahn School of Medicine at Mount Sinai, New York, USA |
| Gupta | Lola | M.P.H. | Cardiothoracic Surgical Trials Network (CTSN) International Coordinating Center (ICC). Icahn School of Medicine at Mount Sinai, New York, USA |
| Overbey | Jessica R. | Dr.P.H. | Cardiothoracic Surgical Trials Network (CTSN) International Coordinating Center (ICC). Icahn School of Medicine at Mount Sinai, New York, USA |
| Santos | Milerva | M.P.A. | Cardiothoracic Surgical Trials Network (CTSN) International Coordinating Center (ICC). Icahn School of Medicine at Mount Sinai, New York, USA |
| Gillinov | Marc A. | M.D. | CTSN Steering Committee Chair, Cleveland Clinic Foundation |
| Miller | Marissa A. | D.V.M, M.P.H. | U.S. National Heart Lung and Blood Institute |
| Taddei-Peters | Wendy C. | Ph.D. | U.S. National Heart Lung and Blood Institute |
| Fenton | Kathleen | M.D., M.S. | U.S. National Heart Lung and Blood Institute |
| Sandkovsky | Uriel | M.D., M.S. | Baylor, Scott and White Health |
| Gottlieb | Robert L. | M.D., Ph.D | Baylor, Scott and White Health |
| Mack | Michael | M.D. | Baylor, Scott and White Health |
| Berhe | Mezgebe | M.D., M.P.H. | Baylor, Scott and White Health |
| Haley | Clinton | M.D., M.P.H. | Baylor, Scott and White Health |
| Dishner | Emma | M.D., M.P.H. | Baylor, Scott and White Health |
| Bettacchi | Christopher | M.D. | Baylor, Scott and White Health |
| Golden | Kevin | M.D. | Baylor, Scott and White Health |
| Duhaime | Erin | P.A.-C. | Baylor, Scott and White Health |
| Ryan | Madison | B.S. | Baylor, Scott and White Health |
| Burris | Sarah | M.H.A. | Baylor, Scott and White Health |
| Tallmadge | Catherine | B.A., C.M.A. | Baylor, Scott and White Health |
| Estrada | Lorie | C.C.R.C. | Baylor, Scott and White Health |
| Jones | Felecia | CRC | Baylor, Scott and White Health |
| Villa | Samatha |  | Baylor, Scott and White Health |
| Wang | Samatha | BSN, RN | Baylor, Scott and White Health |
| Robert | Raven | MPH | Baylor, Scott and White Health |
| Coleman | Tanquinisha |  | Baylor, Scott and White Health |
| Clariday | Laura |  | Baylor, Scott and White Health |
| Baker | Rebecca | BSN. RN | Baylor, Scott and White Health |
| Hurutado-Rodriguez | Mariana | BSN, CCRC, CPXP, CMI | Baylor, Scott and White Health |
| Iram | Nazia | CCRC | Baylor, Scott and White Health |
| Fresnedo | Michelle |  | Baylor, Scott and White Health |
| Davis | Allyson |  | Baylor, Scott and White Health |
| Leonard | Kiara |  | Baylor, Scott and White Health |
| Ramierez | Noelia |  | Baylor, Scott and White Health |
| Thammavong | Jon | B.S. | Baylor, Scott and White Health |
| Duque | Krizia |  | Baylor, Scott and White Health |
| Turner | Emma |  | Baylor, Scott and White Health |
| Fisher | Tammy | M.B.A, M.S.N, R.N, C.C.R.C. | Baylor, Scott and White Health |
| Robinson | Dianna | LVN, CCRC | Baylor, Scott and White Health |
| Ransom | Desirae | MS, CCRC | Baylor, Scott and White Health |
| Maldonado | Nicholas | B.A., A.C.R.P.,-CP | Baylor, Scott and White Health |
| Lusk | Erica | CCRP | Baylor, Scott and White Health |
| Killian | Aaron | PharmD. | Baylor, Scott and White Health |
| Palacious | Adriana | PharmD. | Baylor, Scott and White Health |
| Solis | Edilia | BS, CPhT | Baylor, Scott and White Health |
| Jerrow | Janet |  | Baylor, Scott and White Health |
| Watts | Matthew |  | Baylor, Scott and White Health |
| Whitacre | Heather |  | Baylor, Scott and White Health |
| Cothran | Elizabeth |  | Baylor, Scott and White Health |
| Smith | Peter K. | M.D. | Duke University Health System |
| Barkauskas | Christina E. | M.D. | Duke University Health System |
| Vekstein | Andrew M. | M.D. | Duke University Health System |
| Ko | Emily R. | M.D., PhD. | Duke University Health System |
| Dreyer | Grace R. | PA-C | Duke University Health System |
| Stafford | Neil | M.D. | Duke University Health System |
| Brooks | Megan | M.D. | Duke University Health System |
| Der | Tatyana | M.D. | Duke University Health System |
| Witte | Marie | M.D. | Duke University Health System |
| Gamarallage | Ruwan | M.D. | Duke University Health System |
| Franzone | John | M.D. | Duke University Health System |
| Ivey | Noel | M.D. | Duke University Health System |
| Lumsden | Rebecca H. | M.D. | Duke University Health System |
| Mosaly | Nilima | M.D. | Duke University Health System |
| Mourad | Ahmaad | M.D. | Duke University Health System |
| Holland | Thomas L. | M.D. | Duke University Health System |
| Motta | Mary | B.Sc.N., R.N. | Duke University Health System |
| Lane | Kathleen | B.Sc.N., R.N. | Duke University Health System |
| McGowan | Lauren M. | B.Sc., E.M.T.B. | Duke University Health System |
| Stout | Jennifer | B.Sc. | Duke University Health System |
| Aloor | Heather | M.P.H., M.S. | Duke University Health System |
| Bragg | Kennesha M. | M.S. | Duke University Health System |
| Toledo | Barvina | M.A. | Duke University Health System |
| McLendon-Arvik | Beth | PharmD. | Duke University Health System |
| Bussadori | Barbara | R.P.h. | Duke University Health System |
| Hollister | Beth A. | B.Sc.N., R.N | Duke University Health System |
| Griffin | Michelle | M.P.H., E.M.T.P. | Duke University Health System |
| Giangiacomo | Dana M. |  | Duke University Health System |
| Rodriguez | Vicente | M.D. | Lutheran Medical Group |
| Bokhart | Gordon | PharmD. | Lutheran Medical Group |
| Eichman | Sharon M. |  | Lutheran Medical Group |
| Parrino | Patrick E. | M.D., F.A.C.S. | Ochsner Clinic |
| Spindel | Stephen | M.D. | Ochsner Clinic |
| Bansal | Aditya | M.D. | Ochsner Clinic |
| Baumgarten | Katherine | M.D., F.A.C.P., F.I.D.S.A. | Ochsner Clinic |
| Hand | Johnathan | M.D. | Ochsner Clinic |
| Vonderhaar | Derek | M.D. | Ochsner Clinic |
| Nossaman | Bobby | M.D. | Ochsner Clinic |
| Laudun | Sylvia | D.N.P., M.B.A., R.N., C.P.H.Q. | Ochsner Clinic |
| Ames | DeAnna | M.S. | Ochsner Clinic |
| Broussard | Shane |  | Ochsner Clinic |
| Hernandez | Nilmo |  | Ochsner Clinic |
| Isaac | Geralyn | PharmD. | Ochsner Clinic |
| Dinh | Huan | PharmD. | Ochsner Clinic |
| Zheng | Yiling | PharmD. | Ochsner Clinic |
| Tran | Sonny | PharmD. | Ochsner Clinic |
| McDaniel | Hunter |  | Ochsner Clinic |
| Crovetto | Nicolle | M.S. | Ochsner Clinic |
| Perin | Emerson | M.D., PhD. | Texas Heart Institute |
| Costello | Briana | M.D. | Texas Heart Institute |
| Manian | Prasad | M.D. | Texas Heart Institute |
| Sohail | M. Rizwan | M.D. | Texas Heart Institute |
| Postalian | Alexander | M.D. | Texas Heart Institute |
| Hinsu | Punit | PharmD. | Texas Heart Institute |
| Watson | Carolyn |  | Texas Heart Institute |
| Chen | James | RN | Texas Heart Institute |
| Fink | Melyssa |  | Texas Heart Institute |
| Sturgis | Lydia |  | Texas Heart Institute |
| Walker | Kim |  | Texas Heart Institute |
| Mahon | Kim |  | Texas Heart Institute |
| Parenti | Jennifer | RN | Texas Heart Institute |
| Kappenman | Casey | MS | Texas Heart Institute |
| Knight | Aryn |  | Texas Heart Institute |
| Sturek | Jeffrey M. | M.D., Ph.D. | University of Virginia Health Systems |
| Barros | Andrew | M.D., M.S. | University of Virginia Health Systems |
| Enfield | Kyle B. | M.D., F.C.C.M., S.H.E.A. | University of Virginia Health Systems |
| Kadl | Alexandra | M.D. | University of Virginia Health Systems |
| Green | China J. | B.S., C.C.R.C. | University of Virginia Health Systems |
| Simon | Rachel M. | R.N., B.S.N., C.C.R.C. | University of Virginia Health Systems |
| Fox | Ashley | B.S. | University of Virginia Health Systems |
| Thornton | Kara | PharmD., M.Ed., C.C.R.P. | University of Virginia Health Systems |
| Adams | Amy | PharmD., C.C.R.P. | University of Virginia Health Systems |
| Badhwar | Vinay | M.D. | West Virginia University |
| Sharma | Sunil | M.D. | West Virginia University |
| Peppers | Briana | D.O. | West Virginia University |
| McCarthy | Paul | M.D. | West Virginia University |
| Krupica | Troy | M.D. | West Virginia University |
| Sarwari | Arif | M.D., M.S., M.B.A. | West Virginia University |
| Reece | Rebecca | M.D. | West Virginia University |
| Fornaresico | Lisa | Ph.D. | West Virginia University |
| Glaze | Chad | M.S. | West Virginia University |
| Evans | Raquel | B.S.N., R.N. | West Virginia University |
| Di | Fang | R.N., M.S.N. | West Virginia University |
| Carlson | Shawn | M.D., M.S. | West Virginia University |
| Aucremanne | Tanja | B.S.N., R.N. | West Virginia University |
| Tennant | Connie | B.S.N., R.N. | West Virginia University |
| Giblin Sutton | Lisa | Pharm.D. | West Virginia University |
| Buterbaugh | Sabrina | Pharm.D. | West Virginia University |
| Williams | Roger | C.Ph.T. | West Virginia University |
| Bunner | Robin | B.S. | West Virginia University |
| Traverse | Jay H. | M.D. | Minneapolis Heart Institute Foundation |
| Rhame | Frank | M.D. | Minneapolis Heart Institute Foundation |
| Huelster | Joshua | M.D. | Minneapolis Heart Institute Foundation |
| Kethireddy | Rajesh | M.D. | Minneapolis Heart Institute Foundation |
| Davies | Irena | CCRC | Minneapolis Heart Institute Foundation |
| Salamanca | Julianne | MS | Minneapolis Heart Institute Foundation |
| Majeski | Christine | RN, CCRC | Minneapolis Heart Institute Foundation |
| Skelton | Paige | PharmD. | Minneapolis Heart Institute Foundation |
| Zarambo | Maria | PharmD., B.C.O.P | Minneapolis Heart Institute Foundation |
| Sarafolean | Andrea | RN, CCRC | Minneapolis Heart Institute Foundation |
| Bowdish | Michael E. | M.D., M.S. | University of Southern California |
| Borok | Zea | M.B., Ch.B. | University of Southern California |
| Wald-Dickler | Noah | M.D. | University of Southern California |
| Hutcheon | Douglass | M.D. | University of Southern California |
| Towfighi | Amytis | M.D. | University of Southern California |
| Lee | Mary | M.D. | University of Southern California |
| Lewis | Meghan R. | M.D. | University of Southern California |
| Spellberg | Brad | M.D. | University of Southern California |
| Sher | Linda | M.D. | University of Southern California |
| Sharma | Aniket | M.D. | University of Southern California |
| Olds | Anna P. | M.D. | University of Southern California |
| Justino | Chris | P.A.-C. | University of Southern California |
| Loxano | Edward | M.D. | University of Southern California |
| Romero | Chris | C.R.C. | University of Southern California |
| Leong | Janet | C.R.C. | University of Southern California |
| Rodina | Valentina | M.D. | University of Southern California |
| Quesada | Christine | C.R.C. | University of Southern California |
| Hamilton | Luke |  | University of Southern California |
| Escobar | Jose |  | University of Southern California |
| Leshnower | Brad | M.D., F.A.C.S. | Emory University |
| Bender | William | M.D., M.P.H. | Emory University |
| Sharifpour | Milad | M.D., M.S. | Emory University |
| Miller | Jeffrey | M.D. | Emory University |
| Farrington | Woodrow | M.D. | Emory University |
| Baio | Kim T. | R.N., M.S.N. | Emory University |
| McBride | Mary | R.N., B.S.N., M.A.S. | Emory University |
| Fielding | Michele | R.N., B.S.N., C.C.R.C. | Emory University |
| Mathewson | Sonya | R.N., B.S.N., C.C.R.C. | Emory University |
| Porte | Kristina | B.A:, C.C.R.C. | Emory University |
| Maton | Missy | R.N., B.S.N. | Emory University |
| Ponder | Chari | R.N., B.S.N. | Emory University |
| Haley | Elisabeth | R.N., B.S.N., C.C.R.C. | Emory University |
| Spainhour | Christine | R.N., C.C.R.C. | Emory University |
| Rogers | Susan | R.Ph. | Emory University |
| Tyler | Derrick | C.C.R.P. | Emory University |
| Madathil | Ronson J. | M.D. | University of Maryland |
| Rabin | Joseph | M.D. | University of Maryland |
| Levine | Andrea | M.D. | University of Maryland |
| Saharia | Kapil | M.D. | University of Maryland |
| Tabatabai | Ali | M.D. | University of Maryland |
| Lau | Christine | M.D., M.B.A. | University of Maryland |
| Gammie | James S. | M.D. | University of Maryland |
| Peguero | Maya-Loren |  | University of Maryland |
| McKernan | Kimberly |  | University of Maryland |
| Audette | Mathew |  | University of Maryland |
| Fleischmann | Emily |  | University of Maryland |
| Akbari | Kreshta | M.S. | University of Maryland |
| Lee | Myounghee | Ph.D., Pharm. D. | University of Maryland |
| Chi | Andrew | Pharm.D. | University of Maryland |
| Salehi | Hanna | Pharm.D. | University of Maryland |
| Pariser | Alan | Pharm.D. | University of Maryland |
| Nyguyen | Phuong Tran | Pharm.D. | University of Maryland |
| Moore | Jessica |  | University of Maryland |
| Gee | Adrienne |  | University of Maryland |
| Vincent | Shelika |  | University of Maryland |
| Zuckerman | Richard A. | M.D., M.P.H. | Dartmouth-Hitchcock Medical Center |
| Iribarne | Alexander | M.D., M.S. | Dartmouth-Hitchcock Medical Center |
| Metzler | Sara | B.S.N., R.N. | Dartmouth-Hitchcock Medical Center |
| Shipman | Samantha | B.S.N., R.N. | Dartmouth-Hitchcock Medical Center |
| Johnson | Haley |  | Dartmouth-Hitchcock Medical Center |
| Newton | Crystallee | B.A., C.C.R.C. | Dartmouth-Hitchcock Medical Center |
| Parr | Doug | Pharm.D. | Dartmouth-Hitchcock Medical Center |
| Miller | Leslie | M.D. | BayCare Health System |
| Schelle | Beth | R.N. | BayCare Health System |
| McLean | Sherry | R.N. | BayCare Health System |
| Rothbaum | Howard R. | M.D. | BayCare Health System |
| Alvarez | Michael S. | D.O. | BayCare Health System |
| Kalan | Shivam P. | M.D. | BayCare Health System |
| Germann | Heather H. | M.D. | BayCare Health System |
| Hendershot | Jennifer | Pharm.D., B.C.C.C.P. | BayCare Health System |
| Moroney | Karen | R.N. | BayCare Health System |
| Herring | Karen | R.N. | BayCare Health System |
| Cook | Sharri | R.R.T. | BayCare Health System |
| Paul | Pam |  | BayCare Health System |
| Walker-Ignasiak | Rebecca |  | BayCare Health System |
| North | Crystal | M.D. | Prevention and Early Treatment of Acute Lung Injury (PETAL) ICC, Massachusetts General Hospital, Boston, Massachusetts, USA |
| Oldmixon | Cathryn | R.N. | Prevention and Early Treatment of Acute Lung Injury (PETAL) ICC, Massachusetts General Hospital, Boston, Massachusetts, USA |
| Ringwood | Nancy | B.S.N. | Prevention and Early Treatment of Acute Lung Injury (PETAL) ICC, Massachusetts General Hospital, Boston, Massachusetts, USA |
| Muzikansky | Ariela | R.N., B.A./B.S. | Prevention and Early Treatment of Acute Lung Injury (PETAL) ICC, Massachusetts General Hospital, Boston, Massachusetts, USA |
| Morse | Richard | B.A./B.S. | Prevention and Early Treatment of Acute Lung Injury (PETAL) ICC, Massachusetts General Hospital, Boston, Massachusetts, USA |
| Fitzgerald | Laura | B.A./B.S. | Prevention and Early Treatment of Acute Lung Injury (PETAL) ICC, Massachusetts General Hospital, Boston, Massachusetts, USA |
| Morin | Haley D. | B.S.N. | Prevention and Early Treatment of Acute Lung Injury (PETAL) ICC, Massachusetts General Hospital, Boston, Massachusetts, USA |
| Brower | Roy G. | M.D. | PETAL Steering Committee Chair, Johns Hopkins University |
| Reineck | Lora A. | M.D., M.S. | U.S. National Heart Lung and Blood Institute |
| Bienstock, | Karen | PA-C, M.S. | U.S. National Heart Lung and Blood Institute |
| Steingrub | Jay H. | M.D. | ALIGNE Site Coordinating Center (SCC) Lead Investigators, Baystate Medical Center |
| Hou | Peter K. | M.D. | ALIGNE Site Coordinating Center (SCC) Lead Investigators, Brigham and Women's Hospital |
| Steingrub | Jay S. | M.D. | Baystate Medical Center |
| Tidswell | Mark A. | M.D. | Baystate Medical Center |
| Kozikowski | Lori-Ann | R.N., B.S.N., C.C.R.N. | Baystate Medical Center |
| Kardos | Cynthia | R.N., B.S.N., C.C.R.N. | Baystate Medical Center |
| DeSouza | Leslie |  | Baystate Medical Center |
| Romain | Sarah | R.N., B.S.N. | Baystate Medical Center |
| Thornton-Thompson | Sherell |  | Baystate Medical Center |
| Talmor | Daniel | M.D. | Boston SCC Lead Investigators, Beth Israel Deaconess Medical Center |
| Shapiro | Nathan | M.D. | Boston SCC Lead Investigators, Beth Israel Deaconess Medical Center |
| Andromidas, | Konstantinos |  | Beth Israel Deaconess Medical Center |
| Banner-Goodspeed, | Valerie | M.P.H. | Beth Israel Deaconess Medical Center |
| Bolstad | Michael |  | Beth Israel Deaconess Medical Center |
| Boyle, | Katherine L. | M.D. | Beth Israel Deaconess Medical Center |
| Cabrera | Payton |  | Beth Israel Deaconess Medical Center |
| deVilla, | Arnaldo | R.N., M.P.H. | Beth Israel Deaconess Medical Center |
| Ellis, | Joshua C. | M.D. | Beth Israel Deaconess Medical Center |
| Grafals, | Ana |  | Beth Israel Deaconess Medical Center |
| Hayes | Sharon | R.N. | Beth Israel Deaconess Medical Center |
| Higgins | Conor |  | Beth Israel Deaconess Medical Center |
| Kurt | Lisa |  | Beth Israel Deaconess Medical Center |
| Kurtzman, | Nicholas | M.D. | Beth Israel Deaconess Medical Center |
| Redman, | Kimberly | R.N., B.S.N. | Beth Israel Deaconess Medical Center |
| Rosseto | Elinita |  | Beth Israel Deaconess Medical Center |
| Scaffidi | Douglas |  | Beth Israel Deaconess Medical Center |
| Shapiro, | Nathan | M.D., M.P.H. | Beth Israel Deaconess Medical Center |
| Filbin, | Michael R. | M.D., M.Sc. | Massachusetts General Hospital |
| Hibbert, | Kathryn A. | M.D. | Massachusetts General Hospital |
| Parry, | Blair | C.C.R.C., B.A. | Massachusetts General Hospital |
| Margolin, | Justin | B.S. | Massachusetts General Hospital |
| Hillis, | Brooklynn | B.S.N, R.N. | Massachusetts General Hospital |
| Hamer | Rhonda |  | Massachusetts General Hospital |
| Brait | Kelsey | B.B.A., B.Sc. | Massachusetts General Hospital |
| Beakes | Caroline | B.S. | Massachusetts General Hospital |
| McKaig | Brenna | B.S. | Massachusetts General Hospital |
| Kugener | Eleonore | B.A. | Massachusetts General Hospital |
| Jones | Alan E. | M.D. | University of Mississippi |
| Galbraith | James | M.D. | University of Mississippi |
| Nandi | Utsav | M.D. | University of Mississippi |
| Peacock | Rebekah | R.N. | University of Mississippi |
| Hendey | Gregory | M.D. | California SCC Lead Investigators, David Geffen School of Medicine at UCLA |
| Kangelaris | Kirsten | M.D., M.A.S. | University of California San Francisco |
| Ashktorab | Kimia | B.A. | University of California San Francisco |
| Gropper | Rachel | B.S. | University of California San Francisco |
| Agrawal | Anika | B.S. | University of California San Francisco |
| Yee | Kimberley J. | B.Sc. | University of California San Francisco |
| Jauregui | Alejandra E. | B.A. | University of California San Francisco |
| Zhuo | Hanjing | M.P.H. | University of California San Francisco |
| Almasri | Eyad | M.D. | University of California Fresno |
| Fayed | Mohamed | M.D. | University of California Fresno |
| Hubel | Kinsley A. | M.D. | University of California Fresno |
| Hughes | Alyssa R. | B.S. | University of California Fresno |
| Garcia | Rebekah L. | C.C.R.P. | University of California Fresno |
| Lim | George W. | M.D. | Ronald Reagan UCLA Medical Center |
| Chang | Steven Y. | M.D. | Ronald Reagan UCLA Medical Center |
| Hendey | Gregory | M.D. | Ronald Reagan UCLA Medical Center |
| Lin | Michael Y. | M.D. | Ronald Reagan UCLA Medical Center |
| Vargas | Julia | B.S. | Ronald Reagan UCLA Medical Center |
| Sihota | Hena | B.S. | Ronald Reagan UCLA Medical Center |
| Beutler | Rebecca | M.S. | Ronald Reagan UCLA Medical Center |
| Agarwal | Trisha |  | Ronald Reagan UCLA Medical Center |
| Wilson, | Jennifer G. | M.D., M.S. | Stanford University |
| Vojnik, | Rosemary | B.S. | Stanford University |
| Perez, | Cynthia | B.S. | Stanford University |
| McDowell, | Jordan H. | M.S. | Stanford University |
| Roque | Jonasel | B.S. | Stanford University |
| Wang | Henry | M.D., M.S. | University of Texas Health Science Center |
| Huebinger | Ryan M. | M.D. | University of Texas Health Science Center |
| Patel | Bela | M.D. | University of Texas Health Science Center |
| Vidales | Elizabeth | M.P.H., B.M.S. | University of Texas Health Science Center |
| Albertson | Timothy | M.D. | University of California Davis Health |
| Hardy | Erin | B.A./B.S. | University of California Davis Health |
| Harper | Richart | M.D. | University of California Davis Health |
| Moss | Marc A. | M.D. | Colorado SCC Lead Investigators, University of Colorado Hospital |
| Baduashvili | Amiran | M.D. | University of Colorado Hospital |
| Chauhan | Lakshmi | M.D. | University of Colorado Hospital |
| Douin | David J. | M.D. | University of Colorado Hospital |
| Martinez | Flora | R.N. | University of Colorado Hospital |
| Finck | Lani L. | M.P.H. | University of Colorado Hospital |
| Bastman | Jill | R.N. | University of Colorado Hospital |
| Howell | Michelle | R.N. | University of Colorado Hospital |
| Higgins | Carrie | R.N. | University of Colorado Hospital |
| McKeehan | Jeffrey | M.Sc.N. | University of Colorado Hospital |
| Finigan | Jay | M.D. | National Jewish Health/ St. Joseph Hospital |
| Stubenrauch | Peter | M.D. | National Jewish Health/ St. Joseph Hospital |
| Janssen | William J. | M.D. | National Jewish Health/ St. Joseph Hospital |
| Griesmer | Christine | R.N., M.P.H. | National Jewish Health/ St. Joseph Hospital |
| VerBurg | Olivia | B.A. | National Jewish Health/ St. Joseph Hospital |
| Hyzy | Robert C. | M.D. | Michigan SCC Lead Investigators, University of Michigan |
| Park | Pauline K. | M.D. | Michigan SCC Lead Investigators, University of Michigan |
| Nelson, | Kristine | R.N. | University of Michigan |
| McSparron, | Jake I. | M.D. | University of Michigan |
| Co, | Ivan N. | M.D. | University of Michigan |
| Wang, | Bonnie R. | M.D. | University of Michigan |
| Jimenez, | Jose | M.D. | University of Michigan |
| Olbrich | Norman |  | University of Michigan |
| McDonough | Kelli |  | University of Michigan |
| Jia | Shijing | M.D. | University of Michigan |
| Hanna | Sinan |  | University of Michigan |
| Gong | Michelle N. | M.D., M.S. | Montefiore-Sinai SCC Lead Investigators: Montefiore Medical Center |
| Richardson | Lynne D. | M.D. | Mount Sinai Hospital |
| Nair | Rahul | M.D. | Montefiore Medical Center Moses |
| Lopez | Brenda | M.D. | Montefiore Medical Center Moses |
| Amosu | Omowunmi | M.S. | Montefiore Medical Center Moses |
| Offor | Obiageli | M.D. | Montefiore Medical Center Moses |
| Tzehaie | Hiwet | B.S. | Montefiore Medical Center Moses |
| Nkemdirim | William | M.D. | Montefiore Medical Center Moses |
| Boujid | Sabah | B.S. | Montefiore Medical Center Moses |
| Mosier | Jarrod M. | M.D. | Banner University Medical Center Tucson |
| Hypes | Cameron | M.D. | Banner University Medical Center Tucson |
| Campbell | Elizabeth Salvagio | Ph.D. | Banner University Medical Center Tucson |
| Bixby | Billie | M.D. | Banner University Medical Center Tucson |
| Gilson | Boris | B.A./B.S. | Banner University Medical Center Tucson |
| Lopez | Anitza | B.S. | Banner University Medical Center Tucson |
| Bime | Christian | M.D. | Banner University Medical Center Tucson |
| Parthasarathy | Sairam | M.D. | Banner University Medical Center Tucson |
| Cano | Ariana M. | B.A., B.S. | Banner University Medical Center Tucson |
| Hite | R. Duncan | M.D. | Ohio SCC Lead Investigators, University of Cincinnati |
| Terndrup | Thomas E. | M.D. | Ohio State University |
| Wiedemann | Herbert P. | M.D., M.B.A. | Cleveland Clinic Foundation |
| Hudock | Kristin | M.D. | University of Cincinnati |
| Tanzeem | Hammad | M.D. | University of Cincinnati |
| More | Harshada | M.D. | University of Cincinnati |
| Martinkovic | Jamie | C.N.P. | University of Cincinnati |
| Sellers | Susan | R.N., B.S.N., C.C.R.P. | University of Cincinnati |
| Houston | Judy | PharmD. | University of Cincinnati |
| Burns | Mary | PharmD. | University of Cincinnati |
| Kiran | Simra | M.D. | University of Cincinnati |
| Roads | Tammy | C.C.R.P. | University of Cincinnati |
| Kennedy | Sarah | C.N.P. | University of Cincinnati |
| Duggal | Abhijit | M.D. | Cleveland Clinic Foundation, Cleveland Clinic Fairview Hospital |
| Thiruchelvam | Nirosshan | M.D. | Cleveland Clinic Foundation, Cleveland Clinic Fairview Hospital |
| Ashok | Kiran | B.S. | Cleveland Clinic Foundation, Cleveland Clinic Fairview Hospital |
| King | Alexander H. | M.S. | Cleveland Clinic Foundation, Cleveland Clinic Fairview Hospital |
| Mehkri | Omar | M.D. | Cleveland Clinic Foundation, Cleveland Clinic Fairview Hospital |
| Dugar | Siddharth | M.D. | Cleveland Clinic Foundation, Cleveland Clinic Fairview Hospital |
| Sahoo | Debasis | M.D. | Cleveland Clinic Foundation, Cleveland Clinic Fairview Hospital |
| Yealy | Donald M. | M.D. | University of Pittsburgh Medical Center |
| Angus | Derek C. | M.D. | University of Pittsburgh Medical Center |
| Weissman | Alexandra J. | M.D. | University of Pittsburgh Medical Center |
| Vita | Tina M. | R.N. | University of Pittsburgh Medical Center |
| Berryman | Emily | B.S., B.A. | University of Pittsburgh Medical Center |
| Hough | Catherine L. | M.D. | Pacific Northwest SCC Lead Investigators, Oregon Health and Science University |
| Khan | Akram | M.D. | Oregon Health and Science University |
| Krol | Olivia F. |  | Oregon Health and Science University |
| Mills | Emmanuel | M.D. | Oregon Health and Science University |
| Kinjal | Mistry |  | Oregon Health and Science University |
| Briceno | Genesis |  | Oregon Health and Science University |
| Reddy | Raju | M.D. | Oregon Health and Science University |
| Hubel | Kinsley | M.D. | Oregon Health and Science University |
| Jouzestani | Milad K. |  | Oregon Health and Science University |
| McDougal | Madeline | B.A., B.S. | Oregon Health and Science University |
| Deshmukh | Rupali |  | Oregon Health and Science University |
| Johnston | Nicholas J. | M.D. | Harborview Medical Center, University of Washington Medical Center |
| Robinson | Bryce H. | M.D. | Harborview Medical Center, University of Washington Medical Center |
| Gundel | Staphanie J. | R.D. | Harborview Medical Center, University of Washington Medical Center |
| Katsandres | Sarah C. | B.S. | Harborview Medical Center, University of Washington Medical Center |
| Chen | Peter | M.D. | Cedars-Sinai Medical Center |
| Torbati | Sam S. | M.D. | Cedars-Sinai Medical Center |
| Parimon | Tanyalak | M.D. | Cedars-Sinai Medical Center |
| Caudill | Antonina | M.P.H., C.P.H. | Cedars-Sinai Medical Center |
| Mattison | Brittany |  | Cedars-Sinai Medical Center |
| Jackman | Susan E. | B.S.N., M.S. | Cedars-Sinai Medical Center |
| Chen | Po-En | B.S.N. | Cedars-Sinai Medical Center |
| Bayoumi | Emad | M.D. | Cedars-Sinai Medical Center |
| Ojukwu | Cristabelle | B.S. | Cedars-Sinai Medical Center |
| Fine | Devin | B.S. | Cedars-Sinai Medical Center |
| Weissberg | Gwendolyn | B.S. | Cedars-Sinai Medical Center |
| Isip | Katherine | B.S. | Cedars-Sinai Medical Center |
| Choi-Kuaea | Yunhee | M.S.W. | Cedars-Sinai Medical Center |
| Mehdikhani | Shaunt | M.S. | Cedars-Sinai Medical Center |
| Dar | Tahir B. | Ph.D. | Cedars-Sinai Medical Center |
| Augustin | Nsole Biteghe Fleury | Ph.D. | Cedars-Sinai Medical Center |
| Tran | Dana | B.S. | Cedars-Sinai Medical Center |
| Dukov | Jennifer Emilow | B.S. | Cedars-Sinai Medical Center |
| Matusov | Yuri | M.D. | Cedars-Sinai Medical Center |
| Choe | June | M.D. | Cedars-Sinai Medical Center |
| Hindoyan | Niree A. | B.S. | Cedars-Sinai Medical Center |
| Wynter | Timothy | B.S. | Cedars-Sinai Medical Center |
| Pascual | Ethan | M.A. | Cedars-Sinai Medical Center |
| Clapham | Gregg J. | M.A: | Cedars-Sinai Medical Center |
| Herrera | Lisa |  | Cedars-Sinai Medical Center |
| Caudill | Antonia | M.P.H., C.P.H. | Cedars-Sinai Medical Center |
| O’Mahony | D. Shane | M.D. | Swedish Hospital First Hill |
| Nyatsatsang | Sonam T. | M.D. | Swedish Hospital First Hill |
| Wilson | David M. | M.D. | Swedish Hospital First Hill |
| Wallick | Julie A. | B.A./B.S. | Swedish Hospital First Hill |
| Duven | Alexandria M. | R.N. | Swedish Hospital First Hill |
| Fletcher | Dakota D. | B.S. | Swedish Hospital First Hill |
| Miller | Chadwick | M.D. | Wake Forest Baptist Health |
| Files | D. Clark | M.D. | Wake Forest Baptist Health |
| Gibbs | Kevin W. | M.D. | Wake Forest Baptist Health |
| Flores, | Lori S. | D.N.P. | Wake Forest Baptist Health |
| LaRose | Mary E. | R.N., B.S.N. | Wake Forest Baptist Health |
| Landreth | Leigha D. | R.N., B.S.N. | Wake Forest Baptist Health |
| Palacios | D. Rafael | B.S.C.R. | Wake Forest Baptist Health |
| Parks | Lisa | R.N. | Wake Forest Baptist Health |
| Hicks | Madeline | B.A. | Wake Forest Baptist Health |
| Goodwin | Andrew J. | M.D. | Medical Center of South Carolina |
| Kilb | Edward F. | M.D. | Medical Center of South Carolina |
| Lematty | Caitlan T. | B.S. | Medical Center of South Carolina |
| Patti | Kerilyn |  | Medical Center of South Carolina |
| Grady | Abigail | B.S. | Medical Center of South Carolina |
| Rasberry | April | B.S. | Medical Center of South Carolina |
| Morris | Peter E. | M.D. | University of Kentucky |
| Sturgill | Jamie L. | Ph.D. | University of Kentucky |
| Cassity | Evan P. | M.S. | University of Kentucky |
| Dhar | Sanjay | M.D. | University of Kentucky |
| Montgomery-Yates | Ashley A. | M.D. | University of Kentucky |
| Pasha | Sarah N. | M.D. | University of Kentucky |
| Mayer | Kirby P. | Ph.D. | University of Kentucky |
| Bissel | Brittany | Pharm.D., Ph.D. | University of Kentucky |
| Trott | Terren | M.D. | University of Kentucky |
| Rehman | Shahnaz | M.D. | University of Kentucky |
| de Wit | Marjolein | M.D. | Virginia Commonwealth University |
| Mason | Jessica | M.P.H. | Virginia Commonwealth University |
| Bledsoe | Joseph | M.D. | Intermountain Medical Center |
| Knowlton | Kirk U. | M.D. | Intermountain Medical Center |
| Brown | Samuel | M.D. | Intermountain Medical Center |
| Lanspa | Michael | M.D. | Intermountain Medical Center |
| Leither | Lindsey | M.D. | Intermountain Medical Center |
| Pelton | Ithan | M.D. | Intermountain Medical Center |
| Armbruster | Brent P. | B.S. | Intermountain Medical Center |
| Montgomery | Quinn | B.S., A.E.M.T. | Intermountain Medical Center |
| Kumar | Naresh | M.P.H., C.C.R.P. | Intermountain Medical Center |
| Fergus | Melissa | B.S. | Intermountain Medical Center |
| Imel | Karah | A.S., C.C.R.P. | Intermountain Medical Center |
| Palmer | Ghazal | PharmD. | Intermountain Medical Center |
| Webb | Brandon | M.D. | Intermountain Medical Center |
| Klippel | Carolyn | B.S. | Intermountain Medical Center |
| Jensen | Hannah | B.S. | Intermountain Medical Center |
| Duckworth | Sarah |  | Intermountain Medical Center |
| Gray | Andrew | B.S. | Intermountain Medical Center |
| Burke | Tyler | B.S. | Intermountain Medical Center |
| Knox | Dan | M.D. | Intermountain Medical Center |
| Lumpkin | Jenna | B.S. | Intermountain Medical Center |
| Aston | Valerie T. | M.B.A., R.R.T., C.C.R.P. | Intermountain Medical Center |
| Applegate | Darrin | B.S. | Intermountain Medical Center |
| Serezlic | Erna | B.S. | Intermountain Medical Center |
| Brown | Katie | B.S., R.N. | Intermountain Medical Center |
| Merril | Mardee | B.S., C.C.R.P. | Intermountain Medical Center |
| Harris | Estelle S. | M.D. | University of Utah |
| Middleton | Elizabeth A. | M.D. | University of Utah |
| Barrios | Macy A.G. | B.S. | University of Utah |
| Greer | Jorden | B.S. | University of Utah |
| Schmidt | Amber D. | B.S | University of Utah |
| Webb | Melissa K. | Pharm.D. | University of Utah |
| Paine | Roert | M.D. | University of Utah |
| Callahan | Sean J. | M.D. | University of Utah |
| Waddoups | Lindsey J. | M.S. | University of Utah |
| Yamane | Misty B. | B.S. | University of Utah |
| Self | Wesley H. | M.D., M.P.H. | Vanderbilt SCC Lead Investigators, Vanderbilt University Medical Center |
| Rice | Todd W. | M.D., M.S.C.I. | Vanderbilt SCC Lead Investigators, Vanderbilt University Medical Center |
| Casey | Jonathan D. | M.D., M.S.C.I. | Vanderbilt University Medical Center |
| Johnson | Jakea | M.P.H. | Vanderbilt University Medical Center |
| Gray | Christopher | R.N. | Vanderbilt University Medical Center |
| Hays | Margaret | R.N. | Vanderbilt University Medical Center |
| Roth | Megan | R.N. | Vanderbilt University Medical Center |
|  |  |  | Divison of Clinical Research, NIAID, NIH, ICC |
| Menon | Vidya | M.D, F.A.C.P. | Lincoln Medical Center |
| Kasubhai | Moiz | M.D. | Lincoln Medical Center |
| Pillai | Anjana | M.D. | Lincoln Medical Center |
| Daniel | Jean | M.D., M.A.C.P. | Lincoln Medical Center |
| Sittler | Daniel | M.D. | Lincoln Medical Center |
| Kanna | Balavenkatesh | M.D., M.P.H., F.A.C.P. | Lincoln Medical Center |
| Jilani | Nargis | M.D. | Lincoln Medical Center |
| Amaro | Francisco | R.N., F.N.P.,-BC. | Lincoln Medical Center |
| Santana | Jessica | B.A. | Lincoln Medical Center |
| Lyakovestsky | Aleksandr | PharmD., B.C.P.S. | Lincoln Medical Center |
| Madhoun | Issa | PharmD. | Lincoln Medical Center |
| Desroches | Louis Marie | R.P.H. | Lincoln Medical Center |
| Amadon | Nicole | PharmD., B.C.G.P. | Lincoln Medical Center |
| Bahr | Alaa | PharmD., B.C.P.S. | Lincoln Medical Center |
| Ezzat | Imaan | PharmD., B.H.S.A., M.A. | Lincoln Medical Center |
| Guerrero | Maryanne |  | Lincoln Medical Center |
| Padilla | Joane |  | Lincoln Medical Center |
| Fullmer | Jessie |  | Lincoln Medical Center |
| Singh | Inderpreet |  | Lincoln Medical Center |
| Shah | Syed Hamad Ali |  | Lincoln Medical Center |
| Narang | Rajeev | M.D. | CHRISTUS Spohn Shoreline Hospital |
| Mock | Polly | R.N., C.C.R.C., C.H.R.C. | CHRISTUS Spohn Shoreline Hospital |
| Shadle | Melissa | R.N., B.S.N., O.C.N., C.C.R.C. | CHRISTUS Spohn Shoreline Hospital |
| Hernandez | Brenda | R.N. | CHRISTUS Spohn Shoreline Hospital |
| Welch | Kevin | PharmD. | CHRISTUS Spohn Shoreline Hospital |
| Payne | Andrea | PharmD. | CHRISTUS Spohn Shoreline Hospital |
| Ertl | Gabriela | PharmD. | CHRISTUS Spohn Shoreline Hospital |
| Canario | Daniel | M.D. | Hendrick Medical Center |
| Barrientos | Isabel | M.S.N., A.R.P.N. | Hendrick Medical Center |
| Goss | Danielle | M.P.H., M.H.A. | Hendrick Medical Center |
| DeVries | Mattie | RPh., PharmD. | Hendrick Medical Center |
| Folowosele | Ibidolapo | RPh. | Hendrick Medical Center |
| Garner | Dorothy | M.D. | Carilion Roanoke Memorial Hospital |
| Gomez | Mariana | M.D. | Carilion Roanoke Memorial Hospital |
| Price | Justin | M.D. | Carilion Roanoke Memorial Hospital |
| Bansal | Ekta | M.D. | Carilion Roanoke Memorial Hospital |
| Wong | Jim | M.D. | Carilion Roanoke Memorial Hospital |
| Faulhaber | Jason | M.D. | Carilion Roanoke Memorial Hospital |
| Fazili | Tasaduq | M.D. | Carilion Roanoke Memorial Hospital |
| Yeary | Brian | M.D. | Carilion Roanoke Memorial Hospital |
| Ndolo | Ruth | R.N. | Carilion Roanoke Memorial Hospital |
| Bryant | Christina | R.N. | Carilion Roanoke Memorial Hospital |
| Smigeil | Bridgette | PharmD. | Carilion Roanoke Memorial Hospital |
| Robinson | Philip | M.D. | Hoag Memorial Hospital Presbyterian |
| Najjar | Rana | M.S.H.C.A., C.R.C. | Hoag Memorial Hospital Presbyterian |
| Jones | Patrice | C.R.C. | Hoag Memorial Hospital Presbyterian |
| Nguyen | Julie | R.R.T., C.R.C. | Hoag Memorial Hospital Presbyterian |
| Chin | Christina | PharmD. | Hoag Memorial Hospital Presbyterian |
| Taha | Hassan | M.D. | Cotton O’Neil Clinical Research Center |
| Najm | Salah | M.D., M.B.A. | Cotton O’Neil Clinical Research Center |
| Smith | Christopher | PharmD. | Cotton O’Neil Clinical Research Center |
| Moore | Jason | PharmD. | Cotton O’Neil Clinical Research Center |
| Nassar | Talal | PharmD. | Cotton O’Neil Clinical Research Center |
| Gallinger | Nick | PharmD. | Cotton O’Neil Clinical Research Center |
| Christian | Amy | R.N., C.C.R.C. | Cotton O’Neil Clinical Research Center |
| Mauer | D’Amber | R.N., B.S.N. | Cotton O’Neil Clinical Research Center |
| Phipps | Ashley | R.N., B.S.N. | Cotton O’Neil Clinical Research Center |
| Waters, | Michael | M.D. | Velocity Chula Vista |
| Zepeda | Karla | N.P. | Velocity Chula Vista |
| Coslet | Jordan | P.A. | Velocity Chula Vista |
| Landazuri | Rosalynn | B.S., C.R.C. | Velocity Chula Vista |
| Pineda | Jacob | C.R.C. | Velocity Chula Vista |
| Uribe | Nicole | RPh. | Velocity Chula Vista |
| Garcia | Jose Ruiz | CPhT. | Velocity Chula Vista |
| Barbabosa | Cecilia | R.N. | Velocity Chula Vista |
| Sandler | Kaitlyn | B.S.N. | Velocity Chula Vista |
| Overcash | J. Scott | M.D. | Velocity San Diego |
| Marquez | Adrienna |  | Velocity San Diego |
| Chu | Hanh | M.S.N., NP-C. | Velocity San Diego |
| Lee | Kia | M.S.N., A.N.P.-B.C. | Velocity San Diego |
| Quillin | Kimberly | B.S.N., R.N. | Velocity San Diego |
| Garcia | Andrea | M.S.N., R.N. | Velocity San Diego |
| Lew | Pauline | PharmD. | Velocity San Diego |
| Rogers | Ralph | M.D. | Rhode Island Hospital; The Miriam Hospital |
| Shehadeh | Fadi | M.Sc. | Rhode Island Hospital; The Miriam Hospital |
| Mylona | Evangelia K. | M.Sc. | Rhode Island Hospital; The Miriam Hospital |
| Kaczynski | Matthew | B.Sc. | Rhode Island Hospital; The Miriam Hospital |
| Tran | Quynh-Lam | B.Sc. | Rhode Island Hospital; The Miriam Hospital |
| Benitez | Gregorio | M.P.H. | Rhode Island Hospital; The Miriam Hospital |
| Mishra | Biswajit | Ph.D. | Rhode Island Hospital; The Miriam Hospital |
| Felix | Lewis Oscar | Ph.D. | Rhode Island Hospital; The Miriam Hospital |
| Vafea | Maria Tsikala | M.D. | Rhode Island Hospital; The Miriam Hospital |
| Atalla | Eleftheria | M.D. | Rhode Island Hospital; The Miriam Hospital |
| Davies | Robin | B.S.N., B.A., R.N. | Rhode Island Hospital; The Miriam Hospital |
| Hedili | Salma | C.P.T. | Rhode Island Hospital; The Miriam Hospital |
| Monkeberg | Maria Andrea | M.S., R.Ph., B.C.O.P. | Rhode Island Hospital |
| Tabler | Sandra | R.Ph., B.C.O.P. | Rhode Island Hospital |
| Harrington | Britt | Pharm.D. | The Miriam Hospital |
| Meegada | Sreenath | M.D., | Christus Good Shepard |
| Koripalli | VenkataSandeep | M.D. | Christus Good Shepard |
| Muddana | Prithvi | M.D. | Christus Good Shepard |
| Jain | Lakshay | M.D. | Christus Good Shepard |
| Undavalli | Chaitanya | M.D. | Christus Good Shepard |
| Kavya | Parasa | M.D. | Christus Good Shepard |
| Ibiwoye | Mofoluwaso | M.D. | Christus Good Shepard |
| Akilo | Hameed | M.D. | Christus Good Shepard |
| Lovette, | Bryce D. | PharmD. | Christus Good Shepard |
| Wylie, | Jamie-Crystal | MHA, FACHE, | Christus Good Shepard |
| Smith, | Diana M. | BS | Christus Good Shepard |
| Poon | Kenneth | M.D., F.A.C.P. | Memorial Health Care System |
| Eckardt, | Paula | M.D., F.A.C.P., F.I.D.S.A., A.A.H.I.V.S. | Memorial Health Care System |
| Heysu | Rubio-Gomez, | M.D., F.A.C.P., F.I.D.S.A. | Memorial Health Care System |
| Sundararaman, | Nithya | M.A., M.S., M.B.A. | Memorial Health Care System |
| Alaby, | Doris | B.S.N., R.N. | Memorial Health Care System |
| Sareli | Candice | M.D. | Memorial Health Care System |
| Sánchez | Adriana | M.S. | INSIGHT Washington ICC, Veterans Affairs (VA) Medical Center, Washington, DC |
| Popielski | Laura | M.P.H. | INSIGHT Washington ICC, Veterans Affairs (VA) Medical Center, Washington, DC |
| Kambo | Amy | M.P.H. | INSIGHT Washington ICC, Veterans Affairs (VA) Medical Center, Washington, DC |
| Viens | Kimberley | B.S., C.C.R.P. | INSIGHT Washington ICC, Veterans Affairs (VA) Medical Center, Washington, DC |
| Turner | Melissa | M.S.W. | INSIGHT Washington ICC, Veterans Affairs (VA) Medical Center, Washington, DC |
| Vjecha | Michael J. | M.D. | INSIGHT Washington ICC, Veterans Affairs (VA) Medical Center, Washington, DC |
| Weintrob | Amy | M.D. | INSIGHT Washington ICC, Veterans Affairs (VA) Medical Center, Washington, DC |
| Brar | Indira | M.D. | Henry Ford Health System |
| Markowitz | Norman | M.D. | Henry Ford Health System |
| Pastor | Erika | R.N | Henry Ford Health System |
| Corpuz | Roweena | R.N | Henry Ford Health System |
| Alangaden | George | M.D. | Henry Ford Health System |
| McKinnon | John | M.D. | Henry Ford Health System |
| Ramesh | Mayur | M.D. | Henry Ford Health System |
| Herc | Erica | M.D. | Henry Ford Health System |
| Yared | Nicholas | M.D. | Henry Ford Health System |
| Abreu Lanfranco | Odaliz | M.D. | Henry Ford Health System |
| Rivers | Emanuel | M.D. | Henry Ford Health System |
| Swiderek | Jennifer | M.D. | Henry Ford Health System |
| Hodari Gupta | Ariella | M.D. | Henry Ford Health System |
| Pabla | Pardeep | Pharm D | Henry Ford Health System |
| Eliya | Sonia | Pharm D | Henry Ford Health System |
| Jazrawi | Jehan | RpH | Henry Ford Health System |
| Delor | Jeremy | Pharm D | Henry Ford Health System |
| Desai | Mona | Pharm D | Henry Ford Health System |
| Cook | Aaron |  | Henry Ford Health System |
| Kathrina Jaehne | Anja |  | Henry Ford Health System |
| Kaur Gill | Jasreen |  | Henry Ford Health System |
| Renaud | Sheri |  | Henry Ford Health System |
| Sarveswaran | Siva |  | Henry Ford Health System |
| Gardner | Edward | M.D. | Public Health Institute at Denver Health |
| Scott | James | RN | Public Health Institute at Denver Health |
| Bianchini | Monica | Pharm D | Public Health Institute at Denver Health |
| Melvin | Casey | Pharm D | Public Health Institute at Denver Health |
| Kim | Gina | Pharm D | Public Health Institute at Denver Health |
| Wyles | David | M.D. | Public Health Institute at Denver Health |
| Kamis | Kevin |  | Public Health Institute at Denver Health |
| Miller | Rachel |  | Public Health Institute at Denver Health |
| Douglas | Ivor | MD | Public Health Institute at Denver Health |
| Haukoos | Jason |  | Public Health Institute at Denver Health |
| Hicks | Carrie |  | Public Health Institute at Denver Health |
| Lazarte | Susana | MD | Parkland Health and Hospital Systems |
| Marines-Price | Rubria | Ph.D., D.N.P., A.P.R.N. | Parkland Health and Hospital Systems |
| Osuji | Alice | R.N., B.S.N., M.S.N. | Parkland Health and Hospital Systems; University of Texas Southwestern Medical Center |
| Agbor | Barbine Tchamba Agbor | M.D. | Parkland Health and Hospital Systems |
| Petersen | Tianna | M.Sc., M.S.N. | Parkland Health and Hospital Systems; University of Texas Southwestern Medical Center |
| Kamel | Dena | B.S. | Parkland Health and Hospital Systems; University of Texas Southwestern Medical Center |
| Hansen | Laura | M.A. | Parkland Health and Hospital Systems; University of Texas Southwestern Medical Center |
| Garcia | Angie | M.D. | Parkland Health and Hospital Systems; University of Texas Southwestern Medical Center |
| Cha | Christine | PharmD. | Parkland Health and Hospital Systems |
| Mozaffari | Azadeh | PharmD. | Parkland Health and Hospital Systems |
| Hernandez | Rosa | PharmD., M.B.A. | Parkland Health and Hospital Systems |
| Cutrell | James | MD | University of Texas Southwestern Medical Center |
| Agbor | Barbine Tchamba Agbor | M.D. | University of Texas Southwestern Medical Center |
| Kim | Mina | PharmD. | University of Texas Southwestern Medical Center |
| DellaValle | Natalie | PharmD., B.C.P.S. | University of Texas Southwestern Medical Center |
| Gonzales | Sonia | PharmD., B.C.O.P. | University of Texas Southwestern Medical Center |
| Somboonwit | Charurut | M.D. | University of South Florida, Tampa General Hospital |
| Oxner | Asa | M.D. | University of South Florida, Tampa General Hospital |
| Guerra | Lucy | M.D. | University of South Florida, Tampa General Hospital |
| Hayes | Michael | PharmD. | University of South Florida, Tampa General Hospital |
| Nguyen | Thi | PharmD. | University of South Florida, Tampa General Hospital |
| Tran | Thanh | M.P.H. | University of South Florida, Tampa General Hospital |
| Pinto | Avenette |  | University of South Florida, Tampa General Hospital |
| Hatlen | Timothy | M.D. | Lundquist Institute for Biomedical Innovation |
| Anderson | Betty | B.S. | Lundquist Institute for Biomedical Innovation |
| Zepeda-Gutierrez | Ana | B.S. | Lundquist Institute for Biomedical Innovation |
| Martin | Dannae | B.A. | Lundquist Institute for Biomedical Innovation |
| Temblador | Cindi |  | Lundquist Institute for Biomedical Innovation |
| Cuenca | Avon | B.A. | Lundquist Institute for Biomedical Innovation |
| Tanoviceanu | Roxanne | PharmD. | Lundquist Institute for Biomedical Innovation |
| Prieto | Martha | PharmD. | Lundquist Institute for Biomedical Innovation |
| Guerrero | Mario | M.D. | Lundquist Institute for Biomedical Innovation |
| Martin | Dannae |  | Lundquist Institute for Biomedical Innovation |
| Daar | Eric | M.D. | Lundquist Institute for Biomedical Innovation |
| Correa | Ramiro |  | Lundquist Institute for Biomedical Innovation |
| Hartnell | Gabe |  | Lundquist Institute for Biomedical Innovation |
| Wortmann | Glenn | M.D. | Medstar Health Research Institute |
| Doshi | Saumil | M.D. | Medstar Health Research Institute |
| Moriarty | Theresa | M.S.N.R.N. | Medstar Health Research Institute |
| Gonzales | Melissa | C.R.C., III | Medstar Health Research Institute |
| Garman | Kristin | C.R.N. | Medstar Health Research Institute |
| Baker | Jason V. | M.D. | Hennepin Healthcare Research Institute |
| Frosch | Anne | M.D. | Hennepin Healthcare Research Institute |
| Goldsmith | Rachael | B.Sc. | Hennepin Healthcare Research Institute |
| Driver | Brian | M.D. | Hennepin Healthcare Research Institute |
| Frank | Christine | PharmD | Hennepin Healthcare Research Institute |
| Leviton | Tzivia | PharmD | Hennepin Healthcare Research Institute |
| Prekker | Matthew | M.D. | Hennepin Healthcare Research Institute |
| Jibrell | Hodan | B.Sc. | Hennepin Healthcare Research Institute |
| Lo | Melanie | M.D. | Hennepin Healthcare Research Institute |
| Klaphake | Jonathan | B.Sc. | Hennepin Healthcare Research Institute |
| Mackedanz | Shari | R.N. | Hennepin Healthcare Research Institute |
| Ngo | Linh | M.D. | Hennepin Healthcare Research Institute |
| Garcia-Myers | Kelly | B.Sc. | Hennepin Healthcare Research Institute |
| Kunisaki | Ken M. | M.D., M.S. | Minneapolis VA Medical Center |
| Wendt | Chris | M.D. | Minneapolis VA Medical Center |
| Melzer | Anne | M.D. | Minneapolis VA Medical Center |
| Wetherbee | Erin | M.D. | Minneapolis VA Medical Center |
| Drekonja | Dimitri | M.D. | Minneapolis VA Medical Center |
| Pragman | Alexa | M.D. | Minneapolis VA Medical Center |
| Hamel | Aimee | RN | Minneapolis VA Medical Center |
| Thielen | Abbie | PharmD. | Minneapolis VA Medical Center |
| Kunisaki | Ken M. | M.D., M.S. | Minneapolis VA Medical Center |
| Hassler | Miranda | B.A. | Minneapolis VA Medical Center |
| Walquist | Mary | B.S. | Minneapolis VA Medical Center |
| Augenbraun | Michael | M.D. | SUNY Downstate Medical Center |
| George | Jensen |  | SUNY Downstate Medical Center |
| Demeo | Lynette |  | SUNY Downstate Medical Center |
| Mishko | Motria | PharmD. | SUNY Downstate Medical Center |
| Thomas | Lorraine |  | SUNY Downstate Medical Center |
| Tatem | Luis |  | SUNY Downstate Medical Center |
| Dehovitz | Jack | M.D. | SUNY Downstate Medical Center |
| Abassi | Mahsa | D.O. | University of Minnesota |
| Leuck | Anne-Marie | M.D. | University of Minnesota |
| Rao | Via | M.S. | University of Minnesota |
| Pullen | Matthew | M.D. | University of Minnesota |
| Luke | Darlette | RPh | University of Minnesota |
| LaBar | Derek | PharmD, BCPS | University of Minnesota |
| Christiansen | Theresa | RPh | University of Minnesota |
| Howard | Diondra |  | University of Minnesota |
| Biswas | Kousick | Ph.D. | INSIGHT US Department of Veterans Affairs (VA) ICC |
| Harrington | Cristin | B.A. | INSIGHT US Department of Veterans Affairs (VA) ICC |
| Garcia | Amanda | M.P.H. | INSIGHT US Department of Veterans Affairs (VA) ICC |
| Bremer | Tammy |  | INSIGHT US Department of Veterans Affairs (VA) ICC |
| Burke | Tara |  | INSIGHT US Department of Veterans Affairs (VA) ICC |
| Koker | Brittany | B.S. | INSIGHT US Department of Veterans Affairs (VA) ICC |
| Davis-Karim | Anne | PharmD. | INSIGHT US Department of Veterans Affairs (VA) ICC |
| Pittman | David | B.E. | INSIGHT US Department of Veterans Affairs (VA) ICC |
| Vasudeva | Shikha S. | M.D. | INSIGHT US Department of Veterans Affairs (VA) ICC |
| Johnstone | Jaylynn R. | MPH | INSIGHT US Department of Veterans Affairs (VA) ICC |
| Agnetti | Kate | B.S. | INSIGHT US Department of Veterans Affairs (VA) ICC |
| Davis | Ruby | B.S. | INSIGHT US Department of Veterans Affairs (VA) ICC |
| Trautner | Barbara | M.D., Ph.D. | Michael E. DeBakey VA Medical Center, |
| Hines-Munson | Casey | B.S. | Michael E. DeBakey VA Medical Center, |
| Van | John | B.A. | Michael E. DeBakey VA Medical Center, |
| Dillon | Laura | M.Sc. | Michael E. DeBakey VA Medical Center, |
| Wang | Yiqun | B.S., M.A. | Michael E. DeBakey VA Medical Center, |
| Nagy-Agren | Stephanie | M.D. | Salem VA Medical Center |
| Vasudeva | Shikha | M.D. | Salem VA Medical Center |
| Ochalek | Tracy | B.S.N. | Salem VA Medical Center |
| Caldwell | Erin | D.O. | Salem VA Medical Center |
| Humerickhouse | Edward | M.D. | Salem VA Medical Center |
| Boone | David | D.O. | Salem VA Medical Center |
| McGraw | William | PharmD. | Salem VA Medical Center |
| Looney | David J. | M.D. | VA San Diego Healthcare System |
| Mehta | Sanjay R. | M.D. | VA San Diego Healthcare System |
| Johns | Scott Thompson | PharmD. | VA San Diego Healthcare System |
| St. John | Melissa |  | VA San Diego Healthcare System |
| Raceles | Jacqueline | C.C.R.C. | VA San Diego Healthcare System |
| Sear | Emily | B.S.N, R.N. | VA San Diego Healthcare System |
| Funk | Stephen | PharmD. | VA San Diego Healthcare System |
| Cesarini | Rosa |  | VA San Diego Healthcare System |
| Fang | Michelle | PharmD. | VA San Diego Healthcare System |
| Nicalo | Keith | R.N. | VA San Diego Healthcare System |
| Drake | Wonder | M.D. | VA TVHS Nashville Campus |
| Jones | Beatrice | M.S.N., R.N. | VA TVHS Nashville Campus |
| Holtman | Teresa | DPh. | VA TVHS Nashville Campus |
| Nguyen | Hien H. | M.D. | Sacramento VA Medical Center |
| Maniar | Archana | M.D. | Sacramento VA Medical Center |
| Johnson | Eric A. | M.D. | Sacramento VA Medical Center |
| Nguyen | Lam | B.A. | Sacramento VA Medical Center |
| Tran | Michelle T. | B.S. | Sacramento VA Medical Center |
| Barrett | Thomas W. | M.D., M.C.R. | Portland VA Health Care System |
| Johnston | Tera | B.S. | Portland VA Health Care System |
| Huggins | John T. | M.D. | Charleston VA Medical Center |
| Beiko | Tatsiana Y. | M.D. | Charleston VA Medical Center |
| Hughes | Heather Y. | M.D. | Charleston VA Medical Center |
| McManigle | William C. | M.D. | Charleston VA Medical Center |
| Tanner | Nichole T. | M.D. | Charleston VA Medical Center |
| Washburn | Ronald G. | M.D. | Charleston VA Medical Center |
| Ardelt | Magdalena | A.L.M. | Charleston VA Medical Center |
| Tuohy | Patricia A. | B.S. | Charleston VA Medical Center |
| Mixson | Jennifer L. | RPh. | Charleston VA Medical Center |
| Hinton | Charles G. | PharmD. | Charleston VA Medical Center |
| Thornley | Nicola | M.P.H. | Charleston VA Medical Center |
| Allen | Heather | R.N. | Charleston VA Medical Center |
| Elam | Shannon | R.N. | Charleston VA Medical Center |
| Boatman | Barry | R.N., O.C.N. | Charleston VA Medical Center |
| Baber | Brittany J. |  | Charleston VA Medical Center |
| Ryant | Rudell | M.B.A | Charleston VA Medical Center |
| Roller | Brentin | D.O. | Southern Arizona VA Health Care System |
| Nguyen | Chinh | M.D. | Southern Arizona VA Health Care System |
| Mikail | Amani Morgan | M.S.C.R.M. | Southern Arizona VA Health Care System |
| Hansen | Marivic | Research R.N. | Southern Arizona VA Health Care System |
| Lichtenberger | Paola | M.D. | Miami Bruce Carter VA Health CareSystem |
| Baracco | Gio | M.D. | Miami Bruce Carter VA Health CareSystem |
| Ramos | Carol | M.D. | Miami Bruce Carter VA Health CareSystem |
| Bjork | Lauren | PharmD. | Miami Bruce Carter VA Health CareSystem |
| Sueiro | Melyssa | M.Sc. | Miami Bruce Carter VA Health CareSystem |
| Tien | Phyllis | M.D. | San Francisco VA Health Care System |
| Freasier | Heather | M.Sc., R.D. | San Francisco VA Health Care System |
| Buck | Theresa | M.D. | Bay Pines VA Healthcare System |
| Nekach | Hafida | M.D. | Bay Pines VA Healthcare System |
| Holodniy | Mark | M.D., F.A.C.P., F.I.D.S.A. | Veterans Affairs Palo Alto Health Care System |
| Chary | Aarthi | M.D. | Veterans Affairs Palo Alto Health Care System |
| Lu | Kan | PharmD. | Veterans Affairs Palo Alto Health Care System |
| Peters | Theresa | R.N., M.S., C.C.R.C. | Veterans Affairs Palo Alto Health Care System |
| Lopez | Jessica | C.C.R.C. | Veterans Affairs Palo Alto Health Care System |
| Tan | Susanna Yu | M.D. | VA Long Beach Healthcare System |
| Lee | Robert H. | M.D. | VA Long Beach Healthcare System |
| Asghar | Aliya | M.P.H | VA Long Beach Healthcare System |
| Isip | Tasadduq Karim Karyn | B.A. | VA Long Beach Healthcare System |
| Le | Katherine | PharmD. | VA Long Beach Healthcare System |
| Nguyen | Thao | PharmD. | VA Long Beach Healthcare System |
| Wong | Shinn | PharmD. | VA Long Beach Healthcare System |
| Raben | Dorthe | M.Sc. | INSIGHT Copenhagen ICC, CHIP (Centre of Excellence for Health, Immunity and Infections), Rigshospitalet, University of Copenhagen, Copenhagen, Denmark |
| Murray | Daniel D. | Ph.D. | INSIGHT Copenhagen ICC, CHIP (Centre of Excellence for Health, Immunity and Infections), Rigshospitalet, University of Copenhagen, Copenhagen, Denmark |
| Jensen | Tomas O. | M.D. | INSIGHT Copenhagen ICC, CHIP (Centre of Excellence for Health, Immunity and Infections), Rigshospitalet, University of Copenhagen, Copenhagen, Denmark |
| Peters | Lars | M.D., Ph.D., D.M.Sc. | INSIGHT Copenhagen ICC, CHIP (Centre of Excellence for Health, Immunity and Infections), Rigshospitalet, University of Copenhagen, Copenhagen, Denmark |
| Aagaard | Bitten | B.Sc.N. | INSIGHT Copenhagen ICC, CHIP (Centre of Excellence for Health, Immunity and Infections), Rigshospitalet, University of Copenhagen, Copenhagen, Denmark |
| Nielsen | Charlotte B. |  | INSIGHT Copenhagen ICC, CHIP (Centre of Excellence for Health, Immunity and Infections), Rigshospitalet, University of Copenhagen, Copenhagen, Denmark |
| Krapp | Katharina | Ph.D. | INSIGHT Copenhagen ICC, CHIP (Centre of Excellence for Health, Immunity and Infections), Rigshospitalet, University of Copenhagen, Copenhagen, Denmark |
| Nykjær | Bente Rosdahl |  | INSIGHT Copenhagen ICC, CHIP (Centre of Excellence for Health, Immunity and Infections), Rigshospitalet, University of Copenhagen, Copenhagen, Denmark |
| Olsson | Christina |  | INSIGHT Copenhagen ICC, CHIP (Centre of Excellence for Health, Immunity and Infections), Rigshospitalet, University of Copenhagen, Copenhagen, Denmark |
| Kanne | Katja Lisa | M.Sc., B.Sc.N. | INSIGHT Copenhagen ICC, CHIP (Centre of Excellence for Health, Immunity and Infections), Rigshospitalet, University of Copenhagen, Copenhagen, Denmark |
| Grevsen | Anne Louise | M.Sc., Dent. | INSIGHT Copenhagen ICC, CHIP (Centre of Excellence for Health, Immunity and Infections), Rigshospitalet, University of Copenhagen, Copenhagen, Denmark |
| Joensen | Zillah Maria | B.Sc.N. | INSIGHT Copenhagen ICC, CHIP (Centre of Excellence for Health, Immunity and Infections), Rigshospitalet, University of Copenhagen, Copenhagen, Denmark |
| Bruun | Tina | B.Sc.N. | INSIGHT Copenhagen ICC, CHIP (Centre of Excellence for Health, Immunity and Infections), Rigshospitalet, University of Copenhagen, Copenhagen, Denmark |
| Bojesen | Ane |  | INSIGHT Copenhagen ICC, CHIP (Centre of Excellence for Health, Immunity and Infections), Rigshospitalet, University of Copenhagen, Copenhagen, Denmark |
| Woldbye | Frederik |  | INSIGHT Copenhagen ICC, CHIP (Centre of Excellence for Health, Immunity and Infections), Rigshospitalet, University of Copenhagen, Copenhagen, Denmark |
| Normand, | Nick E. | B.Sc. | INSIGHT Copenhagen ICC, CHIP (Centre of Excellence for Health, Immunity and Infections), Rigshospitalet, University of Copenhagen, Copenhagen, Denmark |
| Esman | Frederik V.L. | B.Sc. | INSIGHT Copenhagen ICC, CHIP (Centre of Excellence for Health, Immunity and Infections), Rigshospitalet, University of Copenhagen, Copenhagen, Denmark |
| Benfield | Thomas | M.D, D.M.Sc. | Denmark Copenhagen University Hospital - Amager and Hvidovre, Center of Research & Disruption of Infectious Diseases, Department of Infectious Diseases |
| Clausen | Clara Lundetoft | M.D. | Denmark Copenhagen University Hospital - Amager and Hvidovre, Center of Research & Disruption of Infectious Diseases, Department of Infectious Diseases |
| Hovmand | Nichlas | M.D. | Denmark Copenhagen University Hospital - Amager and Hvidovre, Center of Research & Disruption of Infectious Diseases, Department of Infectious Diseases |
| Israelsen | Simone Bastrup | M.D. | Denmark Copenhagen University Hospital - Amager and Hvidovre, Center of Research & Disruption of Infectious Diseases, Department of Infectious Diseases |
| Iversen | Katrine | M.D. | Denmark Copenhagen University Hospital - Amager and Hvidovre, Center of Research & Disruption of Infectious Diseases, Department of Infectious Diseases |
| Leding | Caecilie | M.D. | Denmark Copenhagen University Hospital - Amager and Hvidovre, Center of Research & Disruption of Infectious Diseases, Department of Infectious Diseases |
| Pedersen | Karen Brorup | M.D. | Denmark Copenhagen University Hospital - Amager and Hvidovre, Center of Research & Disruption of Infectious Diseases, Department of Infectious Diseases |
| Thorlacius-Ussing | Louise | M.D. | Denmark Copenhagen University Hospital - Amager and Hvidovre, Center of Research & Disruption of Infectious Diseases, Department of Infectious Diseases |
| Tinggaard | Michaela | M.D. | Denmark Copenhagen University Hospital - Amager and Hvidovre, Center of Research & Disruption of Infectious Diseases, Department of Infectious Diseases |
| Tingsgard | Sandra | M.D. | Denmark Copenhagen University Hospital - Amager and Hvidovre, Center of Research & Disruption of Infectious Diseases, Department of Infectious Diseases |
| Krohn-Dehli | Louise | R.N. | Denmark Copenhagen University Hospital - Amager and Hvidovre, Center of Research & Disruption of Infectious Diseases, Department of Infectious Diseases |
| Pedersen | Dorthe | R.N. | Denmark Copenhagen University Hospital - Amager and Hvidovre, Center of Research & Disruption of Infectious Diseases, Department of Infectious Diseases |
| Villadsen | Signe | R.N. | Denmark Copenhagen University Hospital - Amager and Hvidovre, Center of Research & Disruption of Infectious Diseases, Department of Infectious Diseases |
| Jensen | Jens-Ulrik Staehr | M.D., Ph.D. | Herlev-Gentofte Hospital, Respiratory Medicine Section, Department of Internal Medicine |
| Overgaard | Rikke | R.N. | Herlev-Gentofte Hospital, Respiratory Medicine Section, Department of Internal Medicine |
| Rastoder | Ema | M.D. | Herlev-Gentofte Hospital, Respiratory Medicine Section, Department of Internal Medicine |
| Heerfordt | Christian | M.D. | Herlev-Gentofte Hospital, Respiratory Medicine Section, Department of Internal Medicine |
| Hedsund | Caroline | M.D | Herlev-Gentofte Hospital, Respiratory Medicine Section, Department of Internal Medicine |
| Ronn | Christian Phillip | M.D. | Herlev-Gentofte Hospital, Respiratory Medicine Section, Department of Internal Medicine |
| Kamstrup | Peter Thobias | M.D. | Herlev-Gentofte Hospital, Respiratory Medicine Section, Department of Internal Medicine |
| Hogsberg | Dorthe Sandbaek | R.N. | Herlev-Gentofte Hospital, Respiratory Medicine Section, Department of Internal Medicine |
| Bergsoe | Christina | B.Sc. | Herlev-Gentofte Hospital, Respiratory Medicine Section, Department of Internal Medicine |
| Søborg | Christian | M.D., Ph.D. | Herlev-Gentofte Hospital, Respiratory Medicine Section, Department of Internal Medicine |
| Hissabu | Nuria M.S. | B.Sc. | Herlev-Gentofte Hospital, Respiratory Medicine Section, Department of Internal Medicine |
| Arp | Bodil C. | B.Sc. | Herlev-Gentofte Hospital, Respiratory Medicine Section, Department of Internal Medicine |
| Ostergaard | Lars | M.D., Ph.D., D.M.Sc. | Aarhus Universitetshospital, Skejby |
| Staerke | Nina Breinholt | M.D. | Aarhus Universitetshospital, Skejby |
| Yehdego | Yordanos | R.N. | Aarhus Universitetshospital, Skejby |
| Sondergaard | Ane | R.N. | Aarhus Universitetshospital, Skejby |
| Johansen | Isik S. | M.D., D.M.Sc. | Odense University Hospital, Department of Infectious Diseases |
| Arnholdt Pedersen | Andreas | M.D. | Odense University Hospital, Department of Infectious Diseases |
| Knudtzen | Fredrikke C. | M.D. | Odense University Hospital, Department of Infectious Diseases |
| Larsen | Lykke | M.D. | Odense University Hospital, Department of Infectious Diseases |
| Hertz | Mathias A. | M.D. | Odense University Hospital, Department of Infectious Diseases |
| Fabricius | Thilde | M.D. | Odense University Hospital, Department of Infectious Diseases |
| Holden | Inge K. | M.D., Ph.D. | Odense University Hospital, Department of Infectious Diseases |
| Lindvig | Susan O. | M.Sc. | Odense University Hospital, Department of Infectious Diseases |
| Helleberg | Marie | M.D., Ph.D., D.M.Sc. | Dept. of Infectious Diseases, Rigshospitalet, Copenhagen University Hospital |
| Gerstoft | Jan | M.D., D.M.Sc. | Dept. of Infectious Diseases, Rigshospitalet, Copenhagen University Hospital |
| Kirk | Ole | M.D., D.M.Sc. | Dept. of Infectious Diseases, Rigshospitalet, Copenhagen University Hospital |
| Bruun | Tina | R.N., M.Sc., PH. | Dept. of Infectious Diseases, Rigshospitalet, Copenhagen University Hospital |
| Jensen | Tomas Ostergaard | M.D. | North Zealand University Hospital, Department of Pulmonary and Infectious Diseases |
| Madsen | Birgitte Lindegaard | M.D. | North Zealand University Hospital, Department of Pulmonary and Infectious Diseases |
| Pedersen | Thomas Ingemann | M.D. | North Zealand University Hospital, Department of Pulmonary and Infectious Diseases |
| Harboe | Zitta Barrella | M.D. | North Zealand University Hospital, Department of Pulmonary and Infectious Diseases |
| Roge | Birgit Thorup | M.D., Ph.D. | Kolding Hospital, Department of Medicine |
| Hansen | Thomas Michael | M.D. | Kolding Hospital, Department of Medicine |
| Glesner | Matilde Kanstrup | M.D. | Kolding Hospital, Department of Medicine |
| Lofberg | Sandra Valborg | M.D. | Kolding Hospital, Department of Medicine |
| Nielsen | Ariella Denize | M.D. | Kolding Hospital, Department of Medicine |
| Leicht von Huth | Sebastian | M.D., Ph.D. | Kolding Hospital, Department of Medicine |
| Nielsen | Henrik | M.D., D.M.Sci. | Aalborg University Hospital, Department of Infectious Diseases |
| Thisted | Rikke Krog | R.N. | Aalborg University Hospital, Department of Infectious Diseases |
| Petersen | Kristine Toft | R.N. | Aalborg University Hospital, Department of Infectious Diseases |
| Juhl | Maria Ruwald | R.N | Aalborg University Hospital, Department of Infectious Diseases |
| Podlekareva | Daria | M.D., Ph.D. | Department of Respiratory Medicine, Bispebjerg Hospital, Copenhagen, Denmark |
| Johnsen | Stine | M.D., Ph.D. | Department of Respiratory Medicine, Bispebjerg Hospital, Copenhagen, Denmark |
| Andreassen | Helle Frost | M.D., Ph.D. | Department of Respiratory Medicine, Bispebjerg Hospital, Copenhagen, Denmark |
| Pedersen | Lars | M.D., Ph.D. | Department of Respiratory Medicine, Bispebjerg Hospital, Copenhagen, Denmark |
| Lindnér | Cecilia Ebba Clara Ellinor | M.D. | Department of Respiratory Medicine, Bispebjerg Hospital, Copenhagen, Denmark |
| Wiese | Lothar | M.D., Ph.D. | Department of Infectious Diseases, Zealand University Hospital Roskilde and Department of Internal Medicine, Zealand University Hospital Koge |
| Knudsen | Lene Surland | M.D., Ph.D. | Department of Infectious Diseases, Zealand University Hospital Roskilde and Department of Internal Medicine, Zealand University Hospital Koge |
| Nytofte | Nikolaj Julian Skrøder | M.D., Ph.D. | Department of Infectious Diseases, Zealand University Hospital Roskilde and Department of Internal Medicine, Zealand University Hospital Koge |
| Havmøller | Signe Ravn | M.D. | Department of Infectious Diseases, Zealand University Hospital Roskilde and Department of Internal Medicine, Zealand University Hospital Koge |
| Expósito | Maria | B.Sc. | Spain INSIGHT SCC Spain, Hospital Universitari Germans Trias i Pujol, Badalona |
| Badillo | José | B.Sc. | Spain INSIGHT SCC Spain, Hospital Universitari Germans Trias i Pujol, Badalona |
| Martínez | Ana | B.Sc. | Spain INSIGHT SCC Spain, Hospital Universitari Germans Trias i Pujol, Badalona |
| Abad | Elena | B.Sc. | Spain INSIGHT SCC Spain, Hospital Universitari Germans Trias i Pujol, Badalona |
| Chamorro | Ana | B.Sc. | Spain INSIGHT SCC Spain, Hospital Universitari Germans Trias i Pujol, Badalona |
| Figuerola | Ariadna | B.Sc. | Spain INSIGHT SCC Spain, Hospital Universitari Germans Trias i Pujol, Badalona |
| Mateu | Lourdes | M.D., Ph.D. | Hospital Universitari Germans Trias i Pujol, Badalona |
| España | Sergio | M.D. | Hospital Universitari Germans Trias i Pujol, Badalona |
| Lucero | Maria Constanza | MD., Ph.D. | Hospital Universitari Germans Trias i Pujol, Badalona |
| Santos | José Ramón | M.D., Ph.D. | Hospital Universitari Germans Trias i Pujol, Badalona |
| Lladós | Gemma | M.D. | Hospital Universitari Germans Trias i Pujol, Badalona |
| Lopez | Cristina | M.D., Ph.D. | Hospital Universitari Germans Trias i Pujol, Badalona |
| Carabias | Lydia | M.D. | Hospital Universitari Germans Trias i Pujol, Badalona |
| Molina-Morant | Daniel | M.D., Ph.D. | Hospital Universitari Germans Trias i Pujol, Badalona |
| Loste | Cora | M.D., Ph.D. | Hospital Universitari Germans Trias i Pujol, Badalona |
| Bracke | Carmen | M.D. | Hospital Universitari Germans Trias i Pujol, Badalona |
| Siles | Adrian | B.Sc. | Hospital Universitari Germans Trias i Pujol, Badalona |
| Fernández-Cruz | Eduardo | M.D., Ph.D. | Hospital General Universitario Gregorio Marañón, Madrid |
| Natale | Marisa Di | M.D. | Hospital General Universitario Gregorio Marañón, Madrid |
| Padure | Sergiu |  | Hospital General Universitario Gregorio Marañón, Madrid |
| Gomez | Jimena | M.D. | Hospital General Universitario Gregorio Marañón, Madrid |
| Ausin | Cristina | M.D. | Hospital General Universitario Gregorio Marañón, Madrid |
| Cervilla | Eva | M.D. | Hospital General Universitario Gregorio Marañón, Madrid |
| Balastegui | Héctor | M.D. | Hospital General Universitario Gregorio Marañón, Madrid |
| Sainz | Carmen Rodríguez | Ph.D. | Hospital General Universitario Gregorio Marañón, Madrid |
| Lopez | Paco | M.D. | Hospital General Universitario Gregorio Marañón, Madrid |
| Carbone | Javier | M.D., Ph.D. | Hospital General Universitario Gregorio Marañón, Madrid |
| Escobar | Mariam | R.N. | Hospital General Universitario Gregorio Marañón, Madrid |
| Balerdi | Leire | M.D. | Barcelona Institute for Global Health (ISGlobal), Hospital Clínic - Universitat de Barcelona, Barcelona |
| Legarda | Almudena |  | Barcelona Institute for Global Health (ISGlobal), Hospital Clínic - Universitat de Barcelona, Barcelona |
| Roldan | Montserrat |  | Barcelona Institute for Global Health (ISGlobal), Hospital Clínic - Universitat de Barcelona, Barcelona |
| Letona | Laura | M.D. | Barcelona Institute for Global Health (ISGlobal), Hospital Clínic - Universitat de Barcelona, Barcelona |
| Muñoz | José | M.D., Ph.D. | Barcelona Institute for Global Health (ISGlobal), Hospital Clínic - Universitat de Barcelona, Barcelona |
| Camprubí | Daniel | M.D. | Barcelona Institute for Global Health (ISGlobal), Hospital Clínic - Universitat de Barcelona, Barcelona |
| Arribas | Jose R. | M.D. | Hospital Universitario La Paz, IdiPAZ, Madrid |
| Sánchez | Rocio Montejano | M.D., Ph.D. | Hospital Universitario La Paz, IdiPAZ, Madrid |
| Díaz-Pollán | Beatriz | M.D., Ph.D. | Hospital Universitario La Paz, IdiPAZ, Madrid |
| Stewart | Stefan Mark | M.D. | Hospital Universitario La Paz, IdiPAZ, Madrid |
| Garcia | Irene | M.D. | Hospital Universitario La Paz, IdiPAZ, Madrid |
| Borobia | Alberto | M.D., Ph.D. | Hospital Universitario La Paz, IdiPAZ, Madrid |
| Mora-Rillo | Marta | M.D., Ph.D. | Hospital Universitario La Paz, IdiPAZ, Madrid |
| Estrada | Vicente | M.D., Ph.D. | Hospital Clínico San Carlos, Madrid |
| Cabello | Noemi | M.D. | Hospital Clínico San Carlos, Madrid |
| Nuñez-Orantos | M.J. | M.D. | Hospital Clínico San Carlos, Madrid |
| Sagastagoitia | I. | M.D. | Hospital Clínico San Carlos, Madrid |
| Homen | J.R. | MD | Hospital Clínico San Carlos, Madrid |
| Orviz | E. | MD. | Hospital Clínico San Carlos, Madrid |
| Montalvá | Adrián Sánchez | M.D., P.hD. | Hospital Universitary Vall d'Hebron, Barcelona |
| Espinosa-Pereiro | Juan | M.D. | Hospital Universitary Vall d'Hebron, Barcelona |
| Bosch-Nicolau | Pau | M.D. | Hospital Universitary Vall d'Hebron, Barcelona |
| Salvador | Fernando | M.D., P.hD. | Hospital Universitary Vall d'Hebron, Barcelona |
| Burgos | Joaquin | M.D., Ph.D. | Hospital Universitary Vall d'Hebron, Barcelona |
| Morales-Rull | Jose Luis | M.D., Ph.D. | Internal Medicine Department. University Hospital Arnau de Vilanova, Lleida |
| Pena | Anna Maria Moreno | M.D. | Internal Medicine Department. University Hospital Arnau de Vilanova, Lleida |
| Acosta | Cristina | M.D. | Internal Medicine Department. University Hospital Arnau de Vilanova, Lleida |
| Solé-Felip | Cristina | M.D. | Internal Medicine Department. University Hospital Arnau de Vilanova, Lleida |
| Horcajada | Juan P. | M.D., Ph.D. | Hospital del Mar-IMIM, Barcelona |
| Sendra | Elena | M.D. | Hospital del Mar-IMIM, Barcelona |
| Castañeda | Silvia | M.D. | Hospital del Mar-IMIM, Barcelona |
| López-Montesinos | Inmaculada | M.D. | Hospital del Mar-IMIM, Barcelona |
| Gómez-Junyent | Joan | M.D., Ph.D. | Hospital del Mar-IMIM, Barcelona |
| Gonzáles | Carlota Gudiol | M.D. | Infectious Diseases Department, Bellvitge University Hospital, IDIBELL, University of Barcelona, CIBERINFEC |
| Cuervo | Guilermo | M.D., Ph.D. | Infectious Diseases Department, Bellvitge University Hospital, IDIBELL, University of Barcelona, CIBERINFEC |
| Pujol | Miquel | M.D., Ph.D. | Infectious Diseases Department, Bellvitge University Hospital, IDIBELL, University of Barcelona, CIBERINFEC |
| Carratalà | Jordi | M.D., Ph.D. | Infectious Diseases Department, Bellvitge University Hospital, IDIBELL, University of Barcelona, CIBERINFEC |
| Videla | Sebastià | M.D., Ph.D. | Infectious Diseases Department, Bellvitge University Hospital, IDIBELL, University of Barcelona, CIBERINFEC |
| Günthard | Huldrych | M.D. | Switzerland Department of Infectious Diseases and Hospital Epidemiology, University Hospital Zurich and Institute of Medical Virology, University of Zurich, Zurich Switzerland |
| Braun | Dominique L. | M.D. | Switzerland Department of Infectious Diseases and Hospital Epidemiology, University Hospital Zurich and Institute of Medical Virology, University of Zurich, Zurich Switzerland |
| West | Emily | M.D. | Switzerland Department of Infectious Diseases and Hospital Epidemiology, University Hospital Zurich and Institute of Medical Virology, University of Zurich, Zurich Switzerland |
| M’Rabeth-Bensalah | Khadija | M.D. | Switzerland Department of Infectious Diseases and Hospital Epidemiology, University Hospital Zurich and Institute of Medical Virology, University of Zurich, Zurich Switzerland |
| Eichinger | Mareile L. | M.D. | Switzerland Department of Infectious Diseases and Hospital Epidemiology, University Hospital Zurich and Institute of Medical Virology, University of Zurich, Zurich Switzerland |
| Grüttner-Durmaz | Manuela | R.N. | Switzerland Department of Infectious Diseases and Hospital Epidemiology, University Hospital Zurich and Institute of Medical Virology, University of Zurich, Zurich Switzerland |
| Grube | Christina | R.N. | Switzerland Department of Infectious Diseases and Hospital Epidemiology, University Hospital Zurich and Institute of Medical Virology, University of Zurich, Zurich Switzerland |
| Zink | Veronika | M.Sc., pharmacist | Switzerland Department of Infectious Diseases and Hospital Epidemiology, University Hospital Zurich and Institute of Medical Virology, University of Zurich, Zurich Switzerland |
| Goes | Josefine | pharmacist | Switzerland Department of Infectious Diseases and Hospital Epidemiology, University Hospital Zurich and Institute of Medical Virology, University of Zurich, Zurich Switzerland |
| Fätkenheuer | Gerd | M.D. | Department I of Internal Medicine, Division of Infectious Diseases, University of Cologne, Germany |
| Malin | Jakob J. | M.D. | Department I of Internal Medicine, Division of Infectious Diseases, University of Cologne, Germany |
| Tsertsvadze | Tengiz | M.D., Ph.D. | Georgia SCC, Infectious Diseases, AIDS and Clinical Immunology Research Center, Tbilisi, Georgia |
| Abutidze | Akaki | M.D., M.P.H., Ph.D. | Georgia SCC, Infectious Diseases, AIDS and Clinical Immunology Research Center, Tbilisi, Georgia |
| Chkhartishvili | Nikoloz | M.D., M.S., Ph.D. | Georgia SCC, Infectious Diseases, AIDS and Clinical Immunology Research Center, Tbilisi, Georgia |
| Metchurtchlishvili | Revaz | M.D. | Georgia SCC, Infectious Diseases, AIDS and Clinical Immunology Research Center, Tbilisi, Georgia |
| Endeladze | Marina | M.D. | Georgia SCC, Infectious Diseases, AIDS and Clinical Immunology Research Center, Tbilisi, Georgia |
| Paciorek | Marcin | M.D., Ph.D. | Poland SCC, Wojewodzki Szpital Zakazny Warsaw |
| Bursa | Dominik | M.D., Ph.D. | Poland SCC, Wojewodzki Szpital Zakazny Warsaw |
| Krogulec | Dominika | M.D. | Poland SCC, Wojewodzki Szpital Zakazny Warsaw |
| Pulik | Piotr | M.D. | Poland SCC, Wojewodzki Szpital Zakazny Warsaw |
| Ignatowska | Anna | M.D. | Poland SCC, Wojewodzki Szpital Zakazny Warsaw |
| Horban | Andrzej | M.D., Ph.D. | Poland SCC, Wojewodzki Szpital Zakazny Warsaw |
| Bakowska | Elzbieta | M.D. | Poland SCC, Wojewodzki Szpital Zakazny Warsaw |
| Kowaska | Justyna | M.D., Ph.D. | Poland SCC, Wojewodzki Szpital Zakazny Warsaw |
| Bednarska | Agnieszka | M.D., Ph.D. | Poland SCC, Wojewodzki Szpital Zakazny Warsaw |
| Jurek | Natalia | M.D. | Poland SCC, Wojewodzki Szpital Zakazny Warsaw |
| Skrzat-Klapaczynska | Agata | M.D., Ph.D. | Poland SCC, Wojewodzki Szpital Zakazny Warsaw |
| Bienkowski | Carlo | M.D. | Poland SCC, Wojewodzki Szpital Zakazny Warsaw |
| Hackiewicz | Malgorzata | M.D. | Poland SCC, Wojewodzki Szpital Zakazny Warsaw |
| Makowiecki | Michal | M.D. | Poland SCC, Wojewodzki Szpital Zakazny Warsaw |
| Platowski | Antoni | M.D. | Poland SCC, Wojewodzki Szpital Zakazny Warsaw |
| Fishchuk | Roman | M.D | Ukraine Central City Clinical Hospital of Ivano-Frankivsk City, Ukraine |
| Kobrynska | Olena | M.D | Ukraine Central City Clinical Hospital of Ivano-Frankivsk City, Ukraine |
| Levandovska | Khrystyna | M.D | Ukraine Central City Clinical Hospital of Ivano-Frankivsk City, Ukraine |
| Kirieieva | Ivanna |  | Ukraine Central City Clinical Hospital of Ivano-Frankivsk City, Ukraine |
| Kuziuk | Mykhailo |  | Ukraine Central City Clinical Hospital of Ivano-Frankivsk City, Ukraine |
| Naucler | Pontus | M.D., Ph.D. | Sweden, Dept. Of Infectious Diseases, Karolinska University Hospital and Division of Infectious Diseases, Dept. Of Medicine, Solna, Karolinska Instituttet |
| Perlhamre | Emma | M.Sc. | Sweden, Dept. Of Infectious Diseases, Karolinska University Hospital and Division of Infectious Diseases, Dept. Of Medicine, Solna, Karolinska Instituttet |
| Mazouch | Lotta | M.Sc. | Sweden, Dept. Of Infectious Diseases, Karolinska University Hospital and Division of Infectious Diseases, Dept. Of Medicine, Solna, Karolinska Instituttet |
| Kelleher | Anthony | M.B.B.S., Ph.D. | INSIGHT Sydney ICC, The Kirby Institute, University of New South Wales, Sydney, Australia |
| Polizzotto | Mark | M.D., Ph.D. | INSIGHT Sydney ICC, The Kirby Institute, University of New South Wales, Sydney, Australia |
| Carey | Catherine | B.A., M.Sc. | INSIGHT Sydney ICC, The Kirby Institute, University of New South Wales, Sydney, Australia |
| Chang | Christina C. | M.D., Ph.D. | INSIGHT Sydney ICC, The Kirby Institute, University of New South Wales, Sydney, Australia |
| Hough | Sally | B.Sc. | INSIGHT Sydney ICC, The Kirby Institute, University of New South Wales, Sydney, Australia |
| Virachit | Sophie | B.Sc., Ph.D. | INSIGHT Sydney ICC, The Kirby Institute, University of New South Wales, Sydney, Australia |
| Davidson | Sarah | B.N. | INSIGHT Sydney ICC, The Kirby Institute, University of New South Wales, Sydney, Australia |
| Bice | Daniel J. | B.MSc. | INSIGHT Sydney ICC, The Kirby Institute, University of New South Wales, Sydney, Australia |
| Ognenovska | Katherine | B.Sc., Ph.D. | INSIGHT Sydney ICC, The Kirby Institute, University of New South Wales, Sydney, Australia |
| Cabrera | Gesalit | B.MSc., M.I.P.H. | INSIGHT Sydney ICC, The Kirby Institute, University of New South Wales, Sydney, Australia |
| Flynn | Ruth | B.App.Sc., M.App.Sc. | INSIGHT Sydney ICC, The Kirby Institute, University of New South Wales, Sydney, Australia |
| Young | Barnaby E. | M.B.B.S., Ph.D. | National Centre for Infectious Diseases; Tan Tock Seng Hospital; Lee Kong Chian School of Medicine; Singapore |
| Chia | Po Ying | M.B.B.S. | National Centre for Infectious Diseases; Tan Tock Seng Hospital; Lee Kong Chian School of Medicine; Singapore |
| Lee | Tau Hong | M.B.B.S. | National Centre for Infectious Diseases; Tan Tock Seng Hospital; Lee Kong Chian School of Medicine; Singapore |
| Lin | Ray J. | M.B.B.S. | National Centre for Infectious Diseases; Tan Tock Seng Hospital; Woodlands Health; Singapore |
| Lye | David C. | M.B.B.S. | National Centre for Infectious Diseases; Tan Tock Seng Hospital; Yong Loo Lin School of Medicine; Lee Kong Chian School of Medicine; Singapore |
| Ong | Sean W.X. | M.B.B.S. | National Centre for Infectious Diseases; Tan Tock Seng Hospital; Singapore |
| Puah | Ser Hon | M.B.B.S. | Tan Tock Seng Hospital; Singapore |
| Yeo | Tsin Wen | M.B.B.S., Ph.D. | National Centre for Infectious Diseases; Tan Tock Seng Hospital; Lee Kong Chian School of Medicine; Singapore |
| Diong | Shiau Hui | B.Bio., M.Sc. | National Centre for Infectious Diseases; Tan Tock Seng Hospital; Singapore |
| Ongko | Juwinda | B.Sc. | National Centre for Infectious Diseases; Tan Tock Seng Hospital; Singapore |
| Yeo | He Ping | B.Sc. | National Centre for Infectious Diseases; Tan Tock Seng Hospital; Singapore |
| Eriobu | Nnakelu | M.D., M.P.H. | Institute of Human Virology-Nigeria (IHVN) |
| Kwaghe | Vivian | M.D. | Institute of Human Virology-Nigeria (IHVN) |
| Zaiyad | Habib | M.D. | Institute of Human Virology-Nigeria (IHVN) |
| Idoko | Godwin | M.D. | Institute of Human Virology-Nigeria (IHVN) |
| Uche | Blessing | R.M., R.N. | Institute of Human Virology-Nigeria (IHVN) |
| Selvamuthu | Poongulali | M.B.B.S., M.Sc., Ph.D. | Chennai Antiviral Research and Treatment Clinical Research Site, India |
| Kumarasamy | Nagalingeswaran | M.B.B.S., Ph.D. | Chennai Antiviral Research and Treatment Clinical Research Site, India |
| Beulah | Faith Ester | B.Sc., M.A., M.Sc. | Chennai Antiviral Research and Treatment Clinical Research Site, India |
| Govindarajan | Narayan | B.Pharm. | Chennai Antiviral Research and Treatment Clinical Research Site, India |
| Mariyappan | Kowsalya | B.Pharm. | Chennai Antiviral Research and Treatment Clinical Research Site, India |
| Losso | Marcelo H. | M.D., M.S. | INSIGHT SCC Argentina, Coordinación en Investigación Clínica Académica en Latinoamérica |
| Abela | Cecilia | R.N., B.S.N. | INSIGHT SCC Argentina, Coordinación en Investigación Clínica Académica en Latinoamérica |
| Moretto | Renzo | M.D. | INSIGHT SCC Argentina, Coordinación en Investigación Clínica Académica en Latinoamérica |
| Belloc | Carlos G. | B.Sc., Ph.D. | INSIGHT SCC Argentina, Coordinación en Investigación Clínica Académica en Latinoamérica |
| Ludueña | Jael | T.B.A. | INSIGHT SCC Argentina, Coordinación en Investigación Clínica Académica en Latinoamérica |
| Amar | Josefina |  | INSIGHT SCC Argentina, Coordinación en Investigación Clínica Académica en Latinoamérica |
| Losso | Marcelo H. | M.D., M.S. | Hospital General de Agudos JM Ramos Mejia, Buenos Aires |
| Toibaro | Javier | M.D., B.C. | Hospital General de Agudos JM Ramos Mejia, Buenos Aires |
| Macias | Laura Moreno | M.D. | Hospital General de Agudos JM Ramos Mejia, Buenos Aires |
| Fernandez | Lucia | M.D. | Hospital General de Agudos JM Ramos Mejia, Buenos Aires |
| Frare | Pablo S. | M.D. | Hospital General de Agudos JM Ramos Mejia, Buenos Aires |
| Chaio | Sebastian R. | M.D. | Hospital General de Agudos JM Ramos Mejia, Buenos Aires |
| Pachioli | Valeria | M.D. | Hospital General de Agudos JM Ramos Mejia, Buenos Aires |
| Timpano | Stella M. | B.Pharm. | Hospital General de Agudos JM Ramos Mejia, Buenos Aires |
| Sanchez | Marisa del Lujan | M.D. | Hospital Italiano de Buenos Aires, Buenos Aires |
| Sierra | Mariana de Paz | M.D. | Hospital Italiano de Buenos Aires, Buenos Aires |
| Stanek | Vanina | M.D. | Hospital Italiano de Buenos Aires, Buenos Aires |
| Belloso | Waldo | M.D. | Hospital Italiano de Buenos Aires, Buenos Aires |
| Cilenti | Flavia L. |  | Hospital Italiano de Buenos Aires, Buenos Aires |
| Valentini | Ricardo N. | M.D. | Centro de Educacion Medica e Investigaciones Clinicas, Buenos Aires |
| Stryjewski | Martin E. | M.D. | Centro de Educacion Medica e Investigaciones Clinicas, Buenos Aires |
| Locatelli | Nicolas | M.D. | Centro de Educacion Medica e Investigaciones Clinicas, Buenos Aires |
| Soler Riera | Maria C. | M.D. | Centro de Educacion Medica e Investigaciones Clinicas, Buenos Aires |
| Salgado | Clara | M.D. | Centro de Educacion Medica e Investigaciones Clinicas, Buenos Aires |
| Baeck | Ines M. | M.D. | Centro de Educacion Medica e Investigaciones Clinicas, Buenos Aires |
| Di Castelnuovo | Valentina | M.D. | Centro de Educacion Medica e Investigaciones Clinicas, Buenos Aires |
| Zarza | Stella M. | M.D. | Centro de Educacion Medica e Investigaciones Clinicas, Buenos Aires |
| Hudson | Fleur | B.A. | INSIGHT London ICC, MRC Clinical Trials Unit at UC, London, UK |
| Parmar | Mahesh K.B. | Ph.D. | INSIGHT London ICC, MRC Clinical Trials Unit at UC, London, UK |
| Goodman | Anna L. | F.R.C.P., Dphil | INSIGHT London ICC, MRC Clinical Trials Unit at UC, London, UK |
| Badrock | Jonathan | B.Sc. | INSIGHT London ICC, MRC Clinical Trials Unit at UC, London, UK |
| Gregory | Adam | M.A. | INSIGHT London ICC, MRC Clinical Trials Unit at UC, London, UK |
| Goodall | Katharine | B.A. | INSIGHT London ICC, MRC Clinical Trials Unit at UC, London, UK |
| Harris | Nicola |  | INSIGHT London ICC, MRC Clinical Trials Unit at UC, London, UK |
| Wyncoll | James | B.Sc. | INSIGHT London ICC, MRC Clinical Trials Unit at UC, London, UK |
| Bhagani | S. | M.D. | United Kingdom SCC: Royal Free Hospital |
| Rodger | A. | Ph.D. | United Kingdom SCC: Royal Free Hospital |
| Luntiel | A. | M.D. | United Kingdom SCC: Royal Free Hospital |
| Patterson | C. | M.D. | United Kingdom SCC: Royal Free Hospital |
| Morales | J. | B.Sc | United Kingdom SCC: Royal Free Hospital |
| Witele | E. | B.Sc. | United Kingdom SCC: Royal Free Hospital |
| Preston | A-M | B.Sc. | United Kingdom SCC: Royal Free Hospital |
| Nandani | A. | M.Pharm. | United Kingdom SCC: Royal Free Hospital |
| Price | D.A. | M.D. | Royal Victoria Infirmary |
| Hanrath | Aiden | M.B.B.S. | Royal Victoria Infirmary |
| Nell | Jeremy | M.D. | Royal Victoria Infirmary |
| Patel | Bijal | M.Sc. | Royal Victoria Infirmary |
| Hays | Carole | A.D.N.S. | Royal Victoria Infirmary |
| Jones | Geraldine | B.Sc. | Royal Victoria Infirmary |
| Davidson | Jade | B.T.E.C. Pharm | Royal Victoria Infirmary |
| Goodman | Anna L. | F.R.C.P., D.Phil. | Guy’s & St. Thomas’ NHS Foundation Trust |
| Bawa | T. | M.B.B.S. | Guy’s & St. Thomas’ NHS Foundation Trust |
| Mathews | M. | M.Sc., B.Pharm. | Guy’s & St. Thomas’ NHS Foundation Trust |
| Mazzella | A. | M.R.C.P., M.Sc. | Guy’s & St. Thomas’ NHS Foundation Trust |
| Bisnauthsing | K. | B.Sc. | Guy’s & St. Thomas’ NHS Foundation Trust |
| Aguilar-Jimenez | L. | B.Sc. | Guy’s & St. Thomas’ NHS Foundation Trust |
| Borchini | F. | B.Sc. | Guy’s & St. Thomas’ NHS Foundation Trust |
| Hammett | S. | B.Sc. | Guy’s & St. Thomas’ NHS Foundation Trust |
| Touloumi | Giota | Ph.D. | Greece SCC, National & Kapodistrian University of Athens Medical School |
| Pantazis | Nikos | Ph.D. | Greece SCC, National & Kapodistrian University of Athens Medical School |
| Gioukari | Vicky | B.Sc. | Greece SCC, National & Kapodistrian University of Athens Medical School |
| Souliou | Tania |  | Greece SCC, National & Kapodistrian University of Athens Medical School |
| Antoniadou | A. | M.D | Attikon University General Hospital |
| Protopapas | K. | M.D. | Attikon University General Hospital |
| Kavatha | D. | M.D. | Attikon University General Hospital |
| Grigoropoulou | S. | M.D. | Attikon University General Hospital |
| Tziolos | R-N. | M.D. | Attikon University General Hospital |
| Oikonomopoulo | C. |  | Attikon University General Hospital |
| Moschopoulos | C. | M.D. | Attikon University General Hospital |
| Koulouris | N.G. | M.D. | 1st Respiratory Medicine Department, Athens University Medical School |
| Tzimopoulos | K. | M.D. | 1st Respiratory Medicine Department, Athens University Medical School |
| Koromilias | A. | M.D. | 1st Respiratory Medicine Department, Athens University Medical School |
| Argyraki | K. | M.D. | 1st Respiratory Medicine Department, Athens University Medical School |
| Lourida | P. | M.D. | 1st Respiratory Medicine Department, Athens University Medical School |
| Bakakos | P. | M.D. | 1st Respiratory Medicine Department, Athens University Medical School |
| Kalomenidis | I. | M.D. | Department of Critical Care and Pulmonary Medicine, Evangelismos General Hospital |
| Vlachakos | V. | M.D. | Department of Critical Care and Pulmonary Medicine, Evangelismos General Hospital |
| Barmparessou | Z. | M.D. | Department of Critical Care and Pulmonary Medicine, Evangelismos General Hospital |
| Balis | E. | M.D., Ph.D. | Department of Critical Care and Pulmonary Medicine, Evangelismos General Hospital |
| Zakynthinos | S. | M.D. | Department of Critical Care and Pulmonary Medicine, Evangelismos General Hospital |
| Sigala | I. | M.D. | Department of Critical Care and Pulmonary Medicine, Evangelismos General Hospital |
| Gianniou | N. | M.D. | Department of Critical Care and Pulmonary Medicine, Evangelismos General Hospital |
| Dima | E. | M.D. | Department of Critical Care and Pulmonary Medicine, Evangelismos General Hospital |
| Magkouta | S. | M.D. | Department of Critical Care and Pulmonary Medicine, Evangelismos General Hospital |
| Synolaki | E. | M.D. | Department of Critical Care and Pulmonary Medicine, Evangelismos General Hospital |
| Konstanta | S. | M.D. | Department of Critical Care and Pulmonary Medicine, Evangelismos General Hospital |
| Vlachou | M. | M.Sc., Ph.D. | Department of Critical Care and Pulmonary Medicine, Evangelismos General Hospital |
| Stathopoulou | P. | M.Sc. | Department of Critical Care and Pulmonary Medicine, Evangelismos General Hospital |
| Panagopoulos | P. | MD | Democritus University of Thrace |
| Petrakis | V. | MD | Democritus University of Thrace |
| Papazoglou | D. | MD | Democritus University of Thrace |
| Tompaidou | E. | M.Sc. | Democritus University of Thrace |
| Isaakidou | E. |  | Democritus University of Thrace |
| Poulakou | G. | M.D. | 3rd Department of Medicine, Medical School, NKUA |
| Rapti | V. | M.D. | 3rd Department of Medicine, Medical School, NKUA |
| Leontis | K. | M.D. | 3rd Department of Medicine, Medical School, NKUA |
| Nitsotolis | T. | M.D. | 3rd Department of Medicine, Medical School, NKUA |
| Athanasiou | K. | M.D. | 3rd Department of Medicine, Medical School, NKUA |
| Syrigos | K. | M.D. | 3rd Department of Medicine, Medical School, NKUA |
| Argyraki | K. | M.D. | 3rd Department of Medicine, Medical School, NKUA |
| Myrodia | M-D. | M.D. | 3rd Department of Medicine, Medical School, NKUA |
| Kyriakoulis | K. | M.D. | 3rd Department of Medicine, Medical School, NKUA |
| Trontzas | I. | M.D. | 3rd Department of Medicine, Medical School, NKUA |
| Arfara-Melanini | M. | M.D. | 3rd Department of Medicine, Medical School, NKUA |
| Kolonis | V. | M.D. | 3rd Department of Medicine, Medical School, NKUA |
| Kityo | Cissy | M.D. | Uganda SCC, JCRC/MRC/UVRI Uganda Research Unit |
| Mugerwa | Henry | M.D. | Uganda SCC, JCRC/MRC/UVRI Uganda Research Unit |
| Kiweewa | Francis | M.D., M.P.H. | Uganda SCC, JCRC/MRC/UVRI Uganda Research Unit |
| Kimuli | Ivan | M.D. | Uganda SCC, JCRC/MRC/UVRI Uganda Research Unit |
| Lukaakome | Joseph | M.D. | MRC/UVRI & LSHTM Uganda Research Unit |
| Nsereko | Christoher | M.D. | MRC/UVRI & LSHTM Uganda Research Unit |
| Lubega | Gloria | M.D. | MRC/UVRI & LSHTM Uganda Research Unit |
| Kibirige | Moses | M.D. | MRC/UVRI & LSHTM Uganda Research Unit |
| Nakahima | William |  | MRC/UVRI & LSHTM Uganda Research Unit |
| Wangi | Deus |  | MRC/UVRI & LSHTM Uganda Research Unit |
| Aguti | Evelyne | M.D. | MRC/UVRI & LSHTM Uganda Research Unit |
| Generous | Lilian |  | MRC/UVRI & LSHTM Uganda Research Unit |
| Massa | Rosemary |  | MRC/UVRI & LSHTM Uganda Research Unit |
| Nalaki | Margaret |  | MRC/UVRI & LSHTM Uganda Research Unit |
| Magala | Felix | M.D. | MRC/UVRI & LSHTM Uganda Research Unit |
| Nabaggala | Phiona Kaweesi |  | MRC/UVRI & LSHTM Uganda Research Unit |
| Kidega | Robert | M.D. | Gulu Regional Referral Hospital |
| Kityo | Cissy | M.D., Ph.D. | Gulu Regional Referral Hospital |
| Mugerwa | Henry | M.D. | Gulu Regional Referral Hospital |
| Faith | Oryem Daizy | R.N. | Gulu Regional Referral Hospital |
| Florence | Apio | R.N. | Gulu Regional Referral Hospital |
| Emmanuel | Ocung | B.B.L.T. | Gulu Regional Referral Hospital |
| Beacham | Mugoonyi Paul | M.D. | Gulu Regional Referral Hospital |
| Geoffrey | Amone | B.Sc. | Gulu Regional Referral Hospital |
| Nakiboneka | Dridah | B.Stat. | Gulu Regional Referral Hospital |
| Apiyo | Paska | M.D. | Gulu Regional Referral Hospital |
| Kiweewa | Francis | M.D., M.P.H. | Makerere University Lung Institute |
| Kirenga | Bruce | MBChB, NMED Ph.D. | Makerere University Lung Institute |
| Kimuli | Ivan | MBChB, NMED, M.P.H. | Makerere University Lung Institute |
| Atukunda | Angella | MBChB, NMED | Makerere University Lung Institute |
| Muttamba | Winters | MBChB, NMED | Makerere University Lung Institute |
| Remmy | Kyeyume | B.MLT. | Makerere University Lung Institute |
| Segawa | Ivan | B.Pharm. | Makerere University Lung Institute |
| Pheona | Nsubuga | B.Pharm., M.P.H. | Makerere University Lung Institute |
| Kigere | David | D.N. | Makerere University Lung Institute |
| Mbabazi | Queen Lailah | MBChB | Makerere University Lung Institute |
| Boersalino | Ledra | MBChB | Makerere University Lung Institute |
| Nyakoolo | Grace | B.Sc. | Makerere University Lung Institute |
| Kiweewa | Francis | MD., M.P.H. | Lira Regional Referral Hospital |
| Fred | Aniongo | B.MLT. | Lira Regional Referral Hospital |
| Alupo | Alice | R.N. | Lira Regional Referral Hospital |
| Ebong | Doryn | B.S.N. | Lira Regional Referral Hospital |
| Monday | Edson | B.S.N. | Lira Regional Referral Hospital |
| Nalubwama | Ritah Norah | M.D. | Lira Regional Referral Hospital |
| Kainja | Milton | M.D. | Lira Regional Referral Hospital |
| Ambrose | Munu | D.M.L.T. | Lira Regional Referral Hospital |
| Kwehayo | Vanon | R.N. | Lira Regional Referral Hospital |
| Nalubega | Mary Grace | R.N. | Lira Regional Referral Hospital |
| Ongoli | Augustine | MBChB | Lira Regional Referral Hospital |
| Obbo | Stephen | MBChB, NMED, M.P.H. | Lira Regional Referral Hospital |
| Sebudde | Nicholus | MBChB | Lira Regional Referral Hospital |
| Alaba | Jeniffer |  | Lira Regional Referral Hospital |
| Magombe | Geoffrey | B.Pharm. | Lira Regional Referral Hospital |
| Tino | Harriet | B.Pharm., M.Sc.,P.H.S.M. | Lira Regional Referral Hospital |
| Obonya, E.E. | Emmanuel |  | Lira Regional Referral Hospital |
| Lutaakome | Joseph | M.D. | Masaka Regional Referral Hospital |
| Kitonsa | Jonathan | M.D. | Masaka Regional Referral Hospital |
| Onyango | Martin | M.D. | Masaka Regional Referral Hospital |
| Naboth | Tukamwesiga | M.D. | Masaka Regional Referral Hospital |
| Naluyinda | Hadijah |  | Masaka Regional Referral Hospital |
| Nanyunja | Regina |  | Masaka Regional Referral Hospital |
| Irene | Muttiibwa |  | Masaka Regional Referral Hospital |
| Jane | Biira |  | Masaka Regional Referral Hospital |
| Wimfred | Kyobejja |  | Masaka Regional Referral Hospital |
| Leonard | Ssemazzi |  | Masaka Regional Referral Hospital |
| Deus | Tkiinomuhisha |  | Masaka Regional Referral Hospital |
| Babra | Namasaba |  | Masaka Regional Referral Hospital |
| Taire | Paul |  | Masaka Regional Referral Hospital |
| Lutaakome | Joseph | M.D. | St. Francis Hospital, Nsambya |
| Nabankema | Evelyn | M.D. | St. Francis Hospital, Nsambya |
| Ogavu | Joseph | M.D. | St. Francis Hospital, Nsambya |
| Mugerwa | Oscar | M.D. | St. Francis Hospital, Nsambya |
| Okoth | Ivan | M.D. | St. Francis Hospital, Nsambya |
| Mwebaze | Raymond | M.D. | St. Francis Hospital, Nsambya |
| Mugabi | Timothy | M.D. | St. Francis Hospital, Nsambya |
| Makhoba | Anthony | M.D. | St. Francis Hospital, Nsambya |
| Arikiriza | Phiona |  | St. Francis Hospital, Nsambya |
| Theresa | Nabuuma |  | St. Francis Hospital, Nsambya |
| Nakayima | Hope |  | St. Francis Hospital, Nsambya |
| Frank | Kisuule |  | St. Francis Hospital, Nsambya |
| Ramgi | Patrícia | M.D. | CISPOC:  Centro de Investigaçäo e Treino em Saúde da Polana Caniço, Maputo, Mozambique |
| Pereira | Kássia | M.D., and all site team | CISPOC:  Centro de Investigaçäo e Treino em Saúde da Polana Caniço, Maputo, Mozambique |
| Osinusi | Anu | M.D., M.P.H. | Gilead Sciences, Foster City, CA, USA |
| Cao | Huyen | M.D. | Gilead Sciences, Foster City, CA, USA |
| Klekotka | Paul | M.D., Ph.D. | Eli Lilly and Company, Indianapolis, IN |
| Price | Karen | Ph.D. | Eli Lilly and Company, Indianapolis, IN |
| Nirula | Ajay | M.D., Ph.D. | Eli Lilly and Company, Indianapolis, IN |
| Osei | Suzette | M.D., Ph.D. | Vir Biotechnology / GlaxoSmithKline |
| Tipple | Craig | M.B.B.S., M.R.C.P., Ph.D. | Vir Biotechnology / GlaxoSmithKline |
| Wills | Angela | R.N., M.S.N., M.B.A. | Vir Biotechnology / GlaxoSmithKline |
| Peppercorn | Amanda | M.D. | Vir Biotechnology / GlaxoSmithKline |
| Watson | Helen | B.Sc., M.Sc. | Vir Biotechnology / GlaxoSmithKline |
| Gupta | Rajesh | M.D.,M.S., M.P.H. | Vir Biotechnology / GlaxoSmithKline |
| Alexander | Elizabeth | M.D., M.Sc., F.I.D.S.A. | Vir Biotechnology / GlaxoSmithKline |
| Mogalian | Erik | Pharm.D., Ph.D. | Vir Biotechnology / GlaxoSmithKline |
| Lin | Leo | M.D. | Vir Biotechnology / GlaxoSmithKline |
| Ding | Xiao | Ph.D. | Vir Biotechnology / GlaxoSmithKline |
| Margolis | David | M.D., M.P.H. | Brii Biosciences |
| Yan | Li | M.D., Ph.D. | Brii Biosciences |
| Girardet | Jean-Luc | Ph.D. | Brii Biosciences |
| Ma | Ji | Ph.D. | Brii Biosciences |
| Hong | Zhi | Ph.D. | Brii Biosciences |
| Zhu | Quing | Ph.D. | Brii Biosciences |
| Seegobin | Seth | PhD | AstraZeneca |
| Gibbs | Michael | PhD | AstraZeneca |
| Latchman | Mickel | BSc | AstraZeneca |
| Hasior | Katarzyna | MSc | AstraZeneca |
| Bouquet | Jerome | PhD | AstraZeneca |
| Wei | Jianxin | PhD | AstraZeneca |
| Streicher | Katie | PhD | AstraZeneca |
| Schmelzer | Albert | PhD | AstraZeneca |
| Brooks | Dennis | MD, PhD | AstraZeneca |
| Butcher | Jonny | BSc | AstraZeneca |
| Tonev | Dimitar | MD | AstraZeneca |
| Arbetter | Douglas | MPH | AstraZeneca |
| Damstetter | Philippe | MSc | AstraZeneca |
| Legenne | Philippe | M.D | Molecular Partners and Novartis |
| Stumpp | Michael | PhD | Molecular Partners and Novartis |
| Goncalves | Susana | Pharm. D | Molecular Partners and Novartis |
| Ramanathan | Krishnan | Ph.D | Molecular Partners and Novartis |
| Chandra | Richa | M.D. | Molecular Partners and Novartis |
| Baseler | Beth | M.S. | Leidos Biomedical Research, Inc., Frederick, MD, USA |
| Teitelbaum | Marc | M.D. | Leidos Biomedical Research, Inc., Frederick, MD, USA |
| Schechner | Adam | M.D. | Leidos Biomedical Research, Inc., Frederick, MD, USA |
| Holley | H. Preston | M.D. | Leidos Biomedical Research, Inc., Frederick, MD, USA |
| Jankelevich | Shirley | M.D. | Leidos Biomedical Research, Inc., Frederick, MD, USA |
| Adams | Amy | M.S. | Leidos Biomedical Research, Inc., Frederick, MD, USA |
| Becker | Nancy | B.S.N. | Leidos Biomedical Research, Inc., Frederick, MD, USA |
| Dolney | Suzanne | B.S.N. | Leidos Biomedical Research, Inc., Frederick, MD, USA |
| Hissey | Debbie |  | Leidos Biomedical Research, Inc., Frederick, MD, USA |
| Simpson | Shelly | M.S. | Leidos Biomedical Research, Inc., Frederick, MD, USA |
| Kim | Mi Ha | Ph.D. | Leidos Biomedical Research, Inc., Frederick, MD, USA |
| Beeler | Joy | M.P.H. | Leidos Biomedical Research, Inc., Frederick, MD, USA |
| Harmon | Liam | B.A. | Leidos Biomedical Research, Inc., Frederick, MD, USA |
| Asomah | Mabel | M.S.H.S | Leidos Biomedical Research, Inc., Frederick, MD, USA |
| Jato | Yvonne | M.P.H. | Leidos Biomedical Research, Inc., Frederick, MD, USA |
| Stottlemyer | April | A.A. | Leidos Biomedical Research, Inc., Frederick, MD, USA |
| Tang | Olivia | B.S. | Leidos Biomedical Research, Inc., Frederick, MD, USA |
| Vanderpuye | Sharon | B.A. | Leidos Biomedical Research, Inc., Frederick, MD, USA |
| Yeon | Lindsey | B.S. | Leidos Biomedical Research, Inc., Frederick, MD, USA |
| Buehn | Molly | M.S. | Leidos Biomedical Research, Inc., Frederick, MD, USA |
| Eccard-Koons | Vanessa | M.S. | Leidos Biomedical Research, Inc., Frederick, MD, USA |
| Frary | Sadie | M.S. | Leidos Biomedical Research, Inc., Frederick, MD, USA |
| MacDonald | Leah | M.S. | Leidos Biomedical Research, Inc., Frederick, MD, USA |
| Cash | Jennifer | B.S. | Leidos Biomedical Research, Inc., Frederick, MD, USA |
| Hoopengardner | Lisa | M.S. | Leidos Biomedical Research, Inc., Frederick, MD, USA |
| Linton | Jessica | M.S. | Leidos Biomedical Research, Inc., Frederick, MD, USA |
| Schaffhauser | Marylu | B.A. | Leidos Biomedical Research, Inc., Frederick, MD, USA |
| Nelson | Michaela | B.S. | Leidos Biomedical Research, Inc., Frederick, MD, USA |
| Spinelli-Nadzam | Mary | B.S. | Leidos Biomedical Research, Inc., Frederick, MD, USA |
| Proffitt | Calvin | M.A. | Leidos Biomedical Research, Inc., Frederick, MD, USA |
| Lee | Christopher | B.S. | Leidos Biomedical Research, Inc., Frederick, MD, USA |
| Engel | Theresa | M.F.S. | Leidos Biomedical Research, Inc., Frederick, MD, USA |
| Fontaine | Laura | B.S.N. | Leidos Biomedical Research, Inc., Frederick, MD, USA |
| Osborne | C.K. | B.S. | Leidos Biomedical Research, Inc., Frederick, MD, USA |
| Hohn | Matt | M.B.A. | Leidos Biomedical Research, Inc., Frederick, MD, USA |
| Galcik | Michael | M.S. | Leidos Biomedical Research, Inc., Frederick, MD, USA |
| Thompson, | DeeDee | A.A. | Leidos Biomedical Research, Inc., Frederick, MD, USA |
| Kopka | Stacey | M.S. | Leidos Biomedical Research, Inc., Frederick, MD, USA |
| Shelley | Denise M. | M.S. | Leidos Biomedical Research, Inc., Frederick, MD, USA |
| Mendez | Gregg | Ph.D. | Frederick National Laboratory for Cancer Research/Leidos Biomedical Research, Inc., Frederick, MD. |
| Brown | Shawn | M.S. | Frederick National Laboratory for Cancer Research/Leidos Biomedical Research, Inc., Frederick, MD. |
| Albert | Sara | M.P.H. | Leidos Biomedical Research, Inc., Frederick, MD, USA |
| Balde | Abby | M.P.H. | Leidos Biomedical Research, Inc., Frederick, MD, USA |
| Baracz | Michelle | M.S. | Leidos Biomedical Research, Inc., Frederick, MD, USA |
| Bielica | Mona | M.Ed | Leidos Biomedical Research, Inc., Frederick, MD, USA |
| Billouin-Frazier | Shere | M.Sc. | Leidos Biomedical Research, Inc., Frederick, MD, USA |
| Choudary | Jay | M.B.A. | Leidos Biomedical Research, Inc., Frederick, MD, USA |
| Dixon | Mary | A.A. | Leidos Biomedical Research, Inc., Frederick, MD, USA |
| Eyler | Carolyn |  | Leidos Biomedical Research, Inc., Frederick, MD, USA |
| Frye | Leanne | M.A. | Leidos Biomedical Research, Inc., Frederick, MD, USA |
| Gertz | Jensen | M.B.A. | Leidos Biomedical Research, Inc., Frederick, MD, USA |
| Giebeig | Lisa | M.S. | Leidos Biomedical Research, Inc., Frederick, MD, USA |
| Gulati | Neelam | B.S. | Leidos Biomedical Research, Inc., Frederick, MD, USA |
| Hankinson | Liz | B.S. | Leidos Biomedical Research, Inc., Frederick, MD, USA |
| Hogarty | Debi |  | Leidos Biomedical Research, Inc., Frederick, MD, USA |
| Huber | Lynda |  | Leidos Biomedical Research, Inc., Frederick, MD, USA |
| Krauss | Gary | B.S. | Leidos Biomedical Research, Inc., Frederick, MD, USA |
| Lake | Eileen |  | Leidos Biomedical Research, Inc., Frederick, MD, USA |
| Manandhar | Meryan | M.P.H. | Leidos Biomedical Research, Inc., Frederick, MD, USA |
| Rudzinski | Erin | B.S. | Leidos Biomedical Research, Inc., Frederick, MD, USA |
| Sandrus | Jen | A.A. | Leidos Biomedical Research, Inc., Frederick, MD, USA |
| Suders | Connie | M.B.A. | Leidos Biomedical Research, Inc., Frederick, MD, USA |
| Natarajan | Ven | Ph.D. | Frederick National Laboratory for Cancer Research/Leidos Biomedical Research, Inc., Frederick, MD. |
| Rupert | Adam W. | B.S., MT(ASCP) | Frederick National Laboratory for Cancer Research/Leidos Biomedical Research, Inc., Frederick, MD. |
| Baseler | Michael | Ph.D. | Frederick National Laboratory for Cancer Research/Leidos Biomedical Research, Inc., Frederick, MD. |
| Lynam | Danielle | M.S. | Frederick National Laboratory for Cancer Research/Leidos Biomedical Research, Inc., Frederick, MD. |
| Imamichi | Tom | Ph.D. | Frederick National Laboratory for Cancer Research/Leidos Biomedical Research, Inc., Frederick, MD. |
| Laverdure | Sylvain | Ph.D. | Frederick National Laboratory for Cancer Research/Leidos Biomedical Research, Inc., Frederick, MD. |
| McCormack | Ashley | M.P.S. | Frederick National Laboratory for Cancer Research/Leidos Biomedical Research, Inc., Frederick, MD. |
| Paudel | Sharada | Ph.D. | Frederick National Laboratory for Cancer Research/Leidos Biomedical Research, Inc., Frederick, MD. |
| Cook | Kyndal | B.S. | Frederick National Laboratory for Cancer Research/Leidos Biomedical Research, Inc., Frederick, MD. |
| Haupt | Kendra | B.S. | Frederick National Laboratory for Cancer Research/Leidos Biomedical Research, Inc., Frederick, MD. |
| Khan | Ayub | Ph.D. | Frederick National Laboratory for Cancer Research/Leidos Biomedical Research, Inc., Frederick, MD. |
| Hazen | Allison | M.S. | Frederick National Laboratory for Cancer Research/Leidos Biomedical Research, Inc., Frederick, MD. |
| Badralmaa | Yunden | M.S. | Frederick National Laboratory for Cancer Research/Leidos Biomedical Research, Inc., Frederick, MD. |
| Smith | Kenneth |  | Advanced Biomedical Laboratories, LLC., Cinnaminson, NJ, USA |
| Patel | Bhakti |  | Advanced Biomedical Laboratories, LLC., Cinnaminson, NJ, USA |
| Kubernac | Amanda |  | Advanced Biomedical Laboratories, LLC., Cinnaminson, NJ, USA |
| Kubernac | Robert |  | Advanced Biomedical Laboratories, LLC., Cinnaminson, NJ, USA |
| Hoover | Marie L. | Ph.D. | Advanced Biomedical Laboratories, LLC., Cinnaminson, NJ, USA |
| Solomon | Courtney |  | Advanced Biomedical Laboratories, LLC., Cinnaminson, NJ, USA |
| Rashid | Marium |  | Advanced Biomedical Laboratories, LLC., Cinnaminson, NJ, USA |
| Murphy | Joseph |  | Advanced Biomedical Laboratories, LLC., Cinnaminson, NJ, USA |
| Brown | Craig |  | PCI Pharma Services |
| DuChateau | Nadine |  | PCI Pharma Services |
| Ellis | Sadie |  | PCI Pharma Services |
| Flosi | Adam |  | PCI Pharma Services |
| Fox | Lisa |  | PCI Pharma Services |
| Johnson | Les |  | PCI Pharma Services |
| Nelson | Rich |  | PCI Pharma Services |
| Stojanovic | Jelena |  | PCI Pharma Services |
| Treagus | Amy |  | PCI Pharma Services |
| Wenner | Christine |  | PCI Pharma Services |
| Williams | Richard |  | PCI Pharma Services |
